# Discovery of First-in-Class Carbonic Anhydrase/Histone Deacetylase Dual Inhibitors with Antiproliferative Activity in Cancer Cells

**DOI:** 10.1021/acs.jmedchem.5c01788

**Published:** 2025-10-28

**Authors:** Murat Bozdag, Nabil Mroweh, Alessia Raucci, Andrea Angeli, Silvia Peppicelli, Alessio Biagioni, Lido Calorini, Daniela Trisciuoglio, Rino Ragno, Roberta Astolfi, Lidia Giuliani, Clemens Zwergel, Sergio Valente, Elena Andreucci, Fabrizio Carta, Antonello Mai, Claudiu T. Supuran

**Affiliations:** † Università Degli Studi di Firenze, Dipartimento Neurofarba, 9300 Sezione di Scienze Farmaceutiche E Nutraceutiche, Via Ugo Schiff 6, Sesto Fiorentino, Florence I-50019, Italy; ‡ Department of Drug Chemistry & Technologies, 9311 Sapienza University of Rome, P. le a Moro 5, Rome 00185, Italy; § Department of Experimental and Clinical Biomedical Sciences “Mario Serio”, University of Florence, Florence 50134, Italy; ∥ Institute of Molecular Biology and Pathology (IBPM), 196021 National Research Council (CNR), Rome 00185, Italy; ⊥ Pasteur Institute, Cenci-Bolognetti Foundation, Sapienza University of Rome, Piazzale Aldo Moro 5, Rome 00185 Italy

## Abstract

This study reports *in vitro* evidence supporting a new class of compounds capable of independently targeting the tumor-associated human (h) carbonic anhydrase (CA; EC 4.2.1.1) and histone deacetylase (HDAC; EC 3.5.1.98) isoforms as first-in-class agents endowed with enhanced antiproliferative effects and safety profiles when compared to their constitutive counterparts as well as to clinically used drugs. The binding modes of both the CA- and HDAC-directed moieties were investigated through X-ray and molecular modeling experiments, respectively, thus delivering detailed Structure–Activity Relationship (SAR) knowledge.

## Graphical abstract

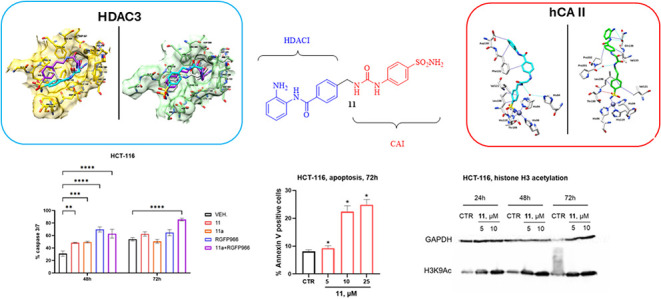

## 1 Introduction

The hypoxia-inducible factor-1 (HIF-1) is a heterodimeric transcription factor composed of the tunable subunit HIF-1α and the constitutively expressed HIF-1β.^1^ Activation of HIF-1 occurs upon specific cellular stimuli, such as reaching intracellular hypoxic thresholds, responding to oncogenic factors, loss of tumor suppressors, or mutations in metabolic enzymes.^2–4^ As a result, a plethora of cellular pathways involved in regulating the genes that encode proteins responsible for pH regulation, cell proliferation, cell adhesion, angiogenesis, and tissue remodeling are activated or modified.^5,6^ Among others, HIF-1 directly regulates the expression of the human (h) Carbonic Anhydrase (CA; EC 4.2.1.1) IX,^7–13^ which is a recently validated tumoral target, with its selective inhibitor **SLC-0111** currently being investigated in Phase II clinical trials in association with **Gemcitabine**.^14^ Large series of investigational CA inhibitors (CAIs) with enhanced selectivity for the tumor-associated hCAs IX (and XII) have been reported in the literature, and a selection^15–19^ is presented in Figure 1.

**Figure 1:**
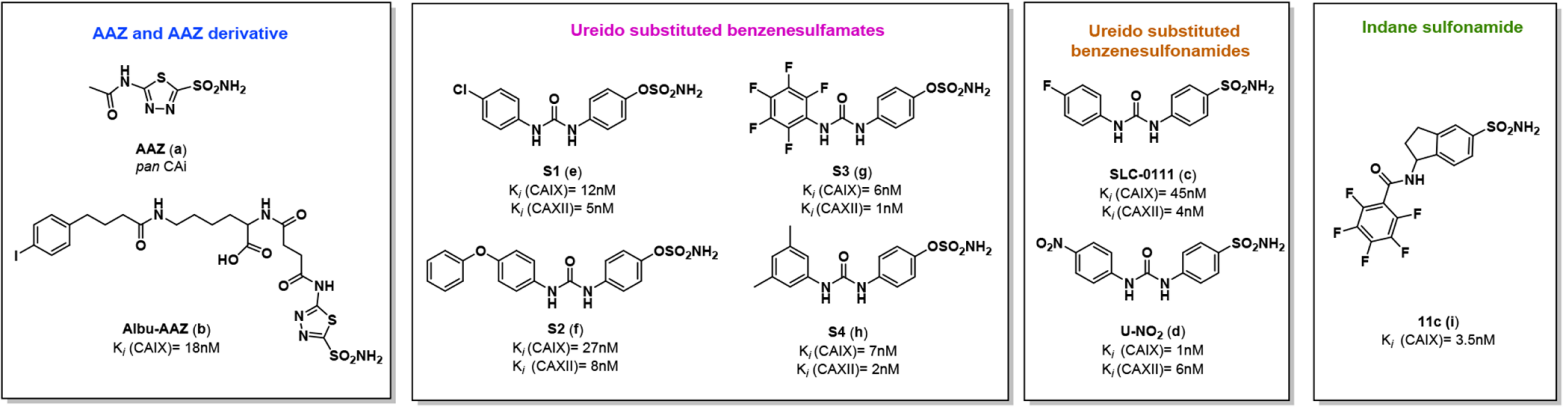
Chemical structures of selected CAIs and their *K*_I_ values on hCA IX and XII isoforms.

Overall, HIF-1 mediates the transcriptional response to hypoxic intracellular stress,^20–22^ which is a feature of solid tumors and/or chronic diseases.^23^ Physiological control of HIF-1α is highly complex, as it requires transcriptional, post-transcriptional, and post-translational modification events. Hydroxylation, ubiquitination, *S*-nitrosation, phosphorylation, and acetylation are the main post-translational modifications that play important roles in regulating the half-life and transcriptional activity of HIF-1α.^24^ Specifically, (de)­acetylation cycles are associated with the stability control of the protein.^25,26^ The deacetylating enzymes, such as histone deacetylase enzymes (HDACs; EC 3.5.1.98), are epigenetic regulatory enzymes that catalyze lysine deacetylation on both histonic and nonhistonic proteins,^27–33^ and among them, the class II isoforms 4 and 6 have HIF-1α as a preferential substrate.^26^ Usually, overexpression of HDACs is a feature of chronic diseases, such as cancer,^27^ and has direct biochemical effects on tumor suppressors,^28–30^ transcription factors,^31,32^ as well as on DNA repair enzymes.^33^ In such a context, the development of HDAC inhibitors (HDACIs) for biomedical purposes began more than 30 years ago and achieved relevant outcomes in the field of epigenetic-related diseases.^27^ To date, several chemical moieties endowed with HDAC inhibition features are known, and they are all based on the ability to chelate the catalytic Zn^2+^ ion^34^ (Figure 2).

**Figure 2:**
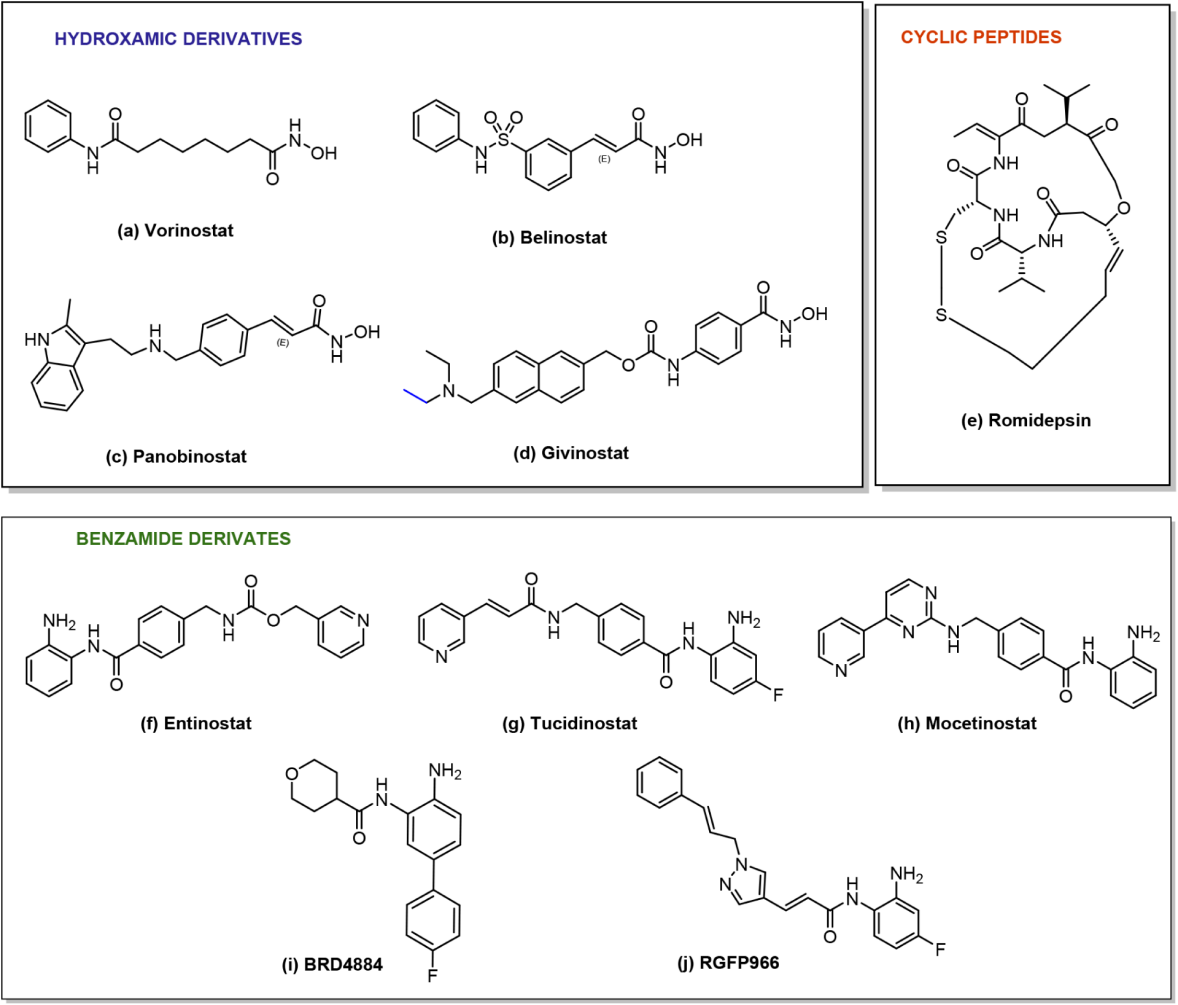
Chemical structures of HDAC inhibitors **a**–**j** approved by the FDA or in clinical trials.

The main group of HDACIs accounts for hydroxamic acid-containing compounds, such as **Vorinostat** (**a**) which was the first HDACI approved by the Food and Drug Administration (FDA) for treating cutaneous refractory T-cell lymphoma (CTCL),^35^ followed by **Belinostat** (**b**) and **Panobinostat** (**c**) for the management of Peripheral T-Cell Lymphoma (PTCL) and Multiple Myeloma (MM), respectively.^36,37^ Recently, the *pan*-HDACI **Givinostat** (**d**) was licensed for Duchenne muscular dystrophy (DMD).^38^ **Romidepsin** (**e**) is a cyclic peptide obtained from *Chromobacterium violaceum* and was approved for CTCL and PTCL.^39^ It acts as a prodrug by reduction of the disulfuric moiety to expose the free sulfides, which act as the metal-chelating species.^39^ Among the 2′-aminoanilide derivatives, there are the class I selective HDACIs **Entinostat** (**f**), currently used in clinical studies for advanced and refractory solid tumors or lymphoma,^40^ and **Tucidinostat** (**g**) approved by the Chinese FDA for PTCL.^41^ In addition, the class I and IV HDACI **Mocetinostat** (**h**) is currently under clinical trials for the treatment of various types of hematological cancers,^42^ such as urothelial carcinoma^43^ or CTCL.^44^ Promising investigational compounds are the HDAC1/2 selective **BRD4884** (**i**)^45,46^ and the HDAC3 selective inhibitor **RGFP966**.^47^

The use of therapeutics in combination is a well-established approach for the treatment of cancers, as it is primarily intended to tackle the biochemical complexity of such diseases.^48–51^ The combination of *pan* or class I-selective HDACIs and CAIs exhibiting synergistic effects is known in the literature.^51,52^ For instance, coadministration of the CAI **Acetazolamide** (**AAZ**) and the HDACI **Entinostat** was reported to induce a stronger decrease in cell viability and migration in neuroblastoma cell lines when compared to the same effects induced by each chemical entity administered singly.^53,54^ Similar results were reported for **Vorinostat** and the Phase II CAI IX **SLC-0111** in HCT116 colorectal carcinoma, MCF7 breast carcinoma, and A375M6 melanoma cell lines.^7^

In this study, we further develop such an approach by reporting first-in-class dual-acting HDACI–CAI compounds bearing the benzamide HDACI moiety linked to the prototypic CAI group of the primary sulfonamide type, and we evaluate their effects on selected cancer cell lines.

## 2 Results and Discussion

### 2.1 Design and Synthesis of Compounds

The chemical strategy adopted in this study was aimed at obtaining HDACI–CAI dual-acting compounds intended for biomedical investigation purposes and bearing chemical moieties specifically directed toward each selected enzyme in order to avoid cross-interactions. In such a context, we designed compounds bearing, at one terminal, the HDAC-directed warhead of the *o*-aminoanilide type, linked by means of a variable spacer to the prototypic CAI pharmacophore of the primary sulfonamide type (Figure 3).

**Figure 3:**
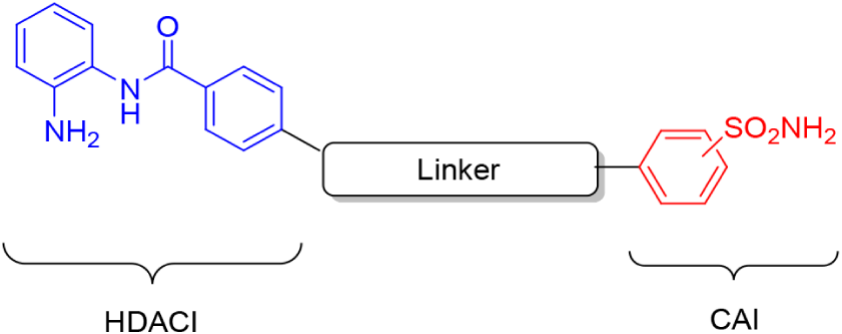
General structure for the HDACI–CAI dual-acting compounds reported in this study.

We began with the synthesis of the minimal HDAC–CA-directed functional unit by straightforward coupling of the commercially available *o-*aminoaniline (**1**) with 4-sulfamoylbenzoic acid (**2**) or 4-chloro-3-sulfamoylbenzoic acid (**3**) to afford the monoamide derivatives **4** and **5**, respectively (Scheme 1).

**Scheme 1:**
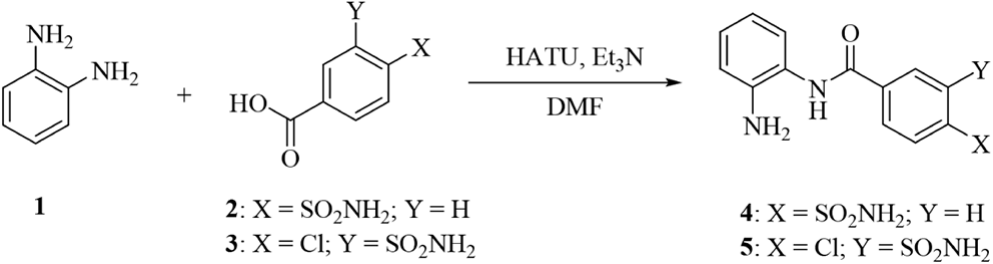
Synthesis of Compounds **4** and **5**

Specifically, the switch of the primary sulfonamide moiety from the 4- to 3-position was intended to exploit the ligand’s warhead fitting within the hCA cavity in order to compensate for its lack of conformational freedom due to the amide group.

Thus, we directed our synthetic work toward the insertion of appropriate spacers between the two warheads. The multistep approach in Scheme 2 was successfully adopted to obtain the key intermediate **6**, which makes use of the *para*-disubstituted phenyl ring to take part in the *o-*aminoanilide tail from the primary sulfonamide head and, in analogy to **4** and **5**, it preserved conformational restraints due to the amide linkages on both section ends.

**Scheme 2:**
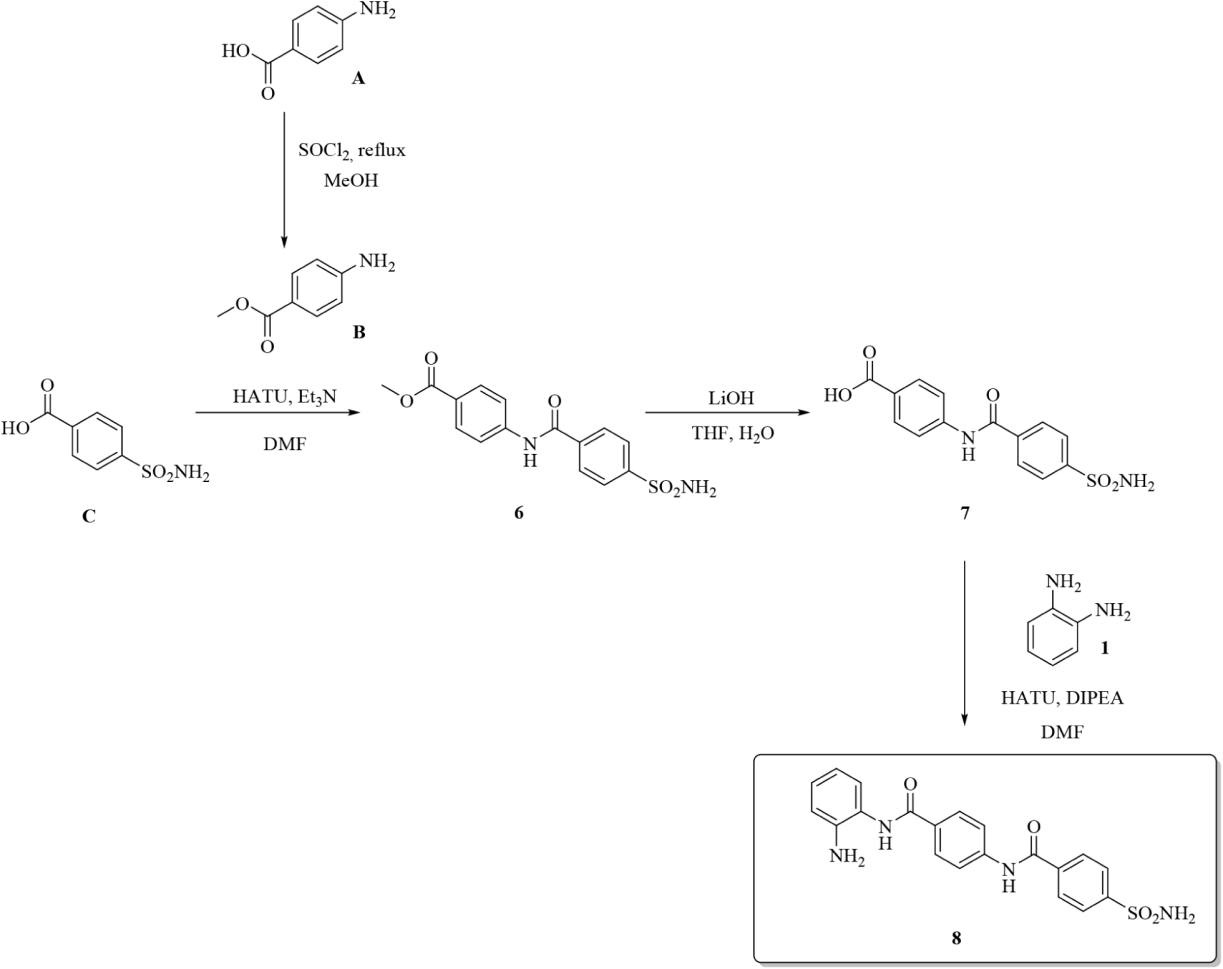
Synthesis of Compound **8**

The highly efficient molecular elongation approach in Scheme 2 was achieved by means of standard amide coupling reactions on the appropriate substrates **C** and **7** and represented the blueprint we further adapted to gain access to a variety of linear and highly flexible derivatives, such as the Entinostat/Tucidinostat-related compounds **11**, **12**, and **14** in Scheme 3.

**Scheme 3:**
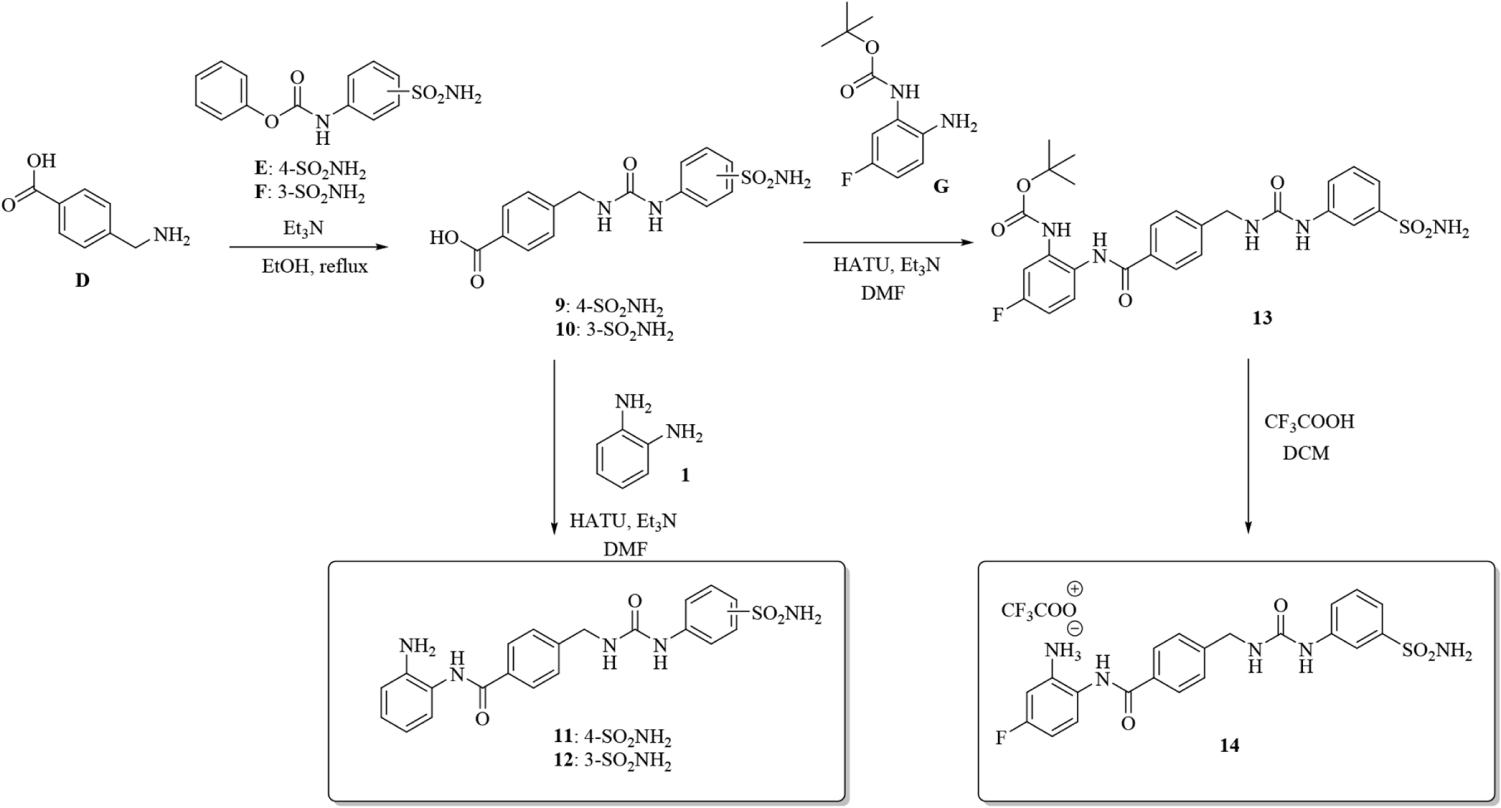
Synthesis of Compounds **11**, **12**, and **14**

The key intermediates **9** and **10** were obtained by reacting the commercially available 4-(aminomethyl)­benzoic acid **D** with the freshly prepared carbamates **E** and **F**, and then coupled with **1** to afford **11** and **12,** respectively, as single reaction products. Since the couplings on **9** and **10** resulted in a statistical mixture of products when the 4-fluorosubstituted *o-*aminoanilide was used instead (data not shown), we employed its monoprotected derivative **G**, which, however, successfully worked only on the **10** intermediate. The desired final compound **14** was obtained upon *N*-Boc cleavage under standard conditions (Scheme 3).

Chemical manipulation, as shown in Scheme 4 allowed us to make use of the three main transformations previously explored (i.e., acyl substitution, hydrolysis, and amide coupling) to migrate the CA-relevant ureido group toward the benzamide moiety.

**Scheme 4:**
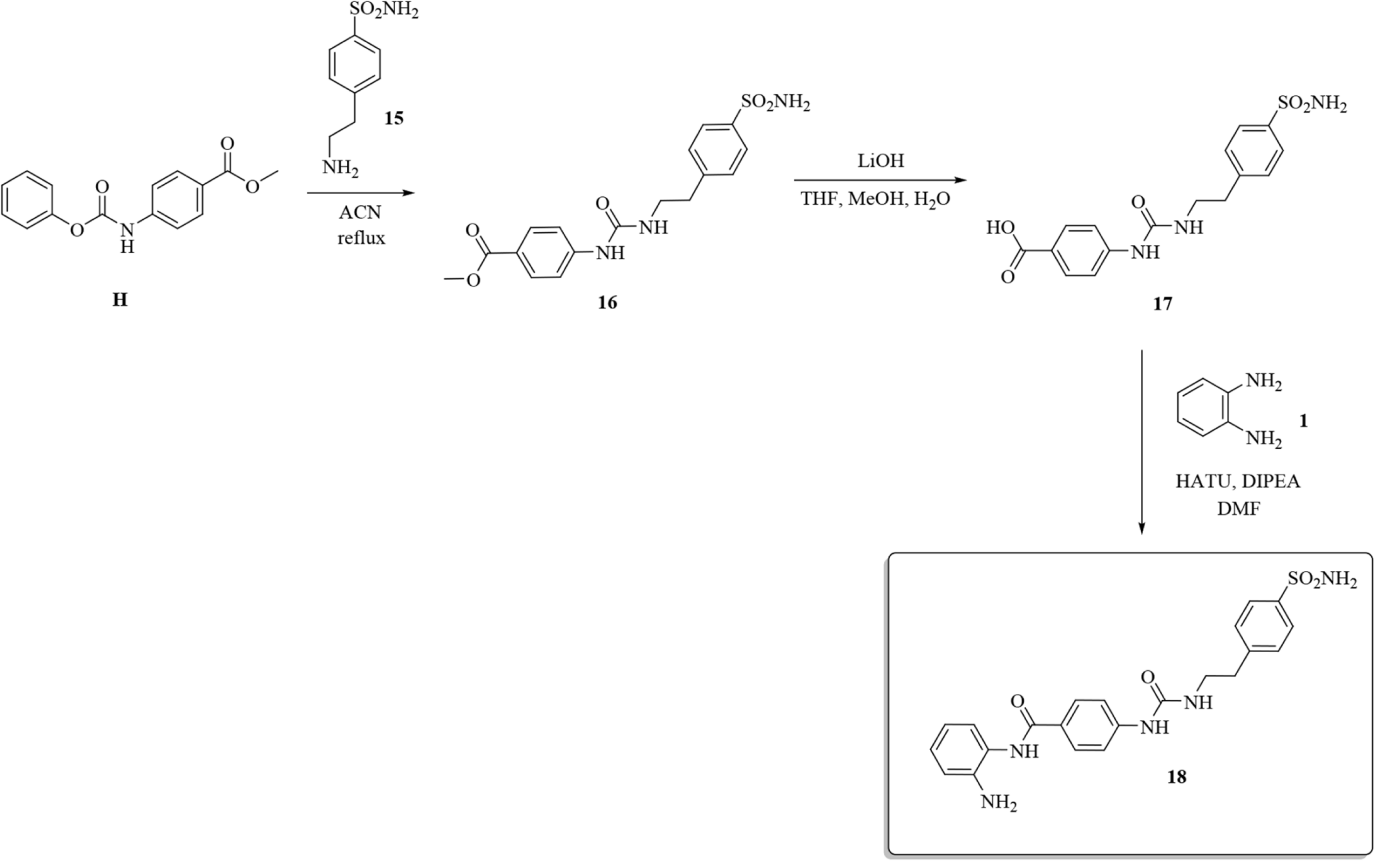
Synthesis of Compound **18**

Specifically, the ureido group was readily installed by treating freshly prepared phenyl carbamate **H** with 4-(2-aminoethyl)­benzenesulfonamide **15** to give ureido intermediate **16**. The synthesis of dual hybrid derivative **18** was terminated by coupling free carboxylic acid **17** with *o-*aminoaniline **1**.

Additional investigations encompassed the isosteric thioureido group, which was readily installed on the commercially available 4-aminobenzoic acid **A** by adding the freshly prepared aryl isothiocyanates **I** and **J** to afford **19** and **20**, respectively (Scheme 5). The aminoanilide moiety, as in **21** and **22**, was obtained in agreement with the coupling procedures reported above.

**Scheme 5:**
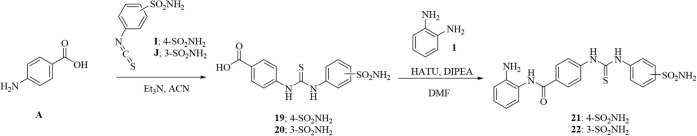
Synthesis of Thioureido-Containing Compounds **21** and **22**

Molecular elongation and conformational enhancement of thioureido derivatives **21** and **22** were accomplished by inserting the aminoanilide group into free acids **27** and **28** to afford **29** and **30**, respectively (Scheme 6).

**Scheme 6:**
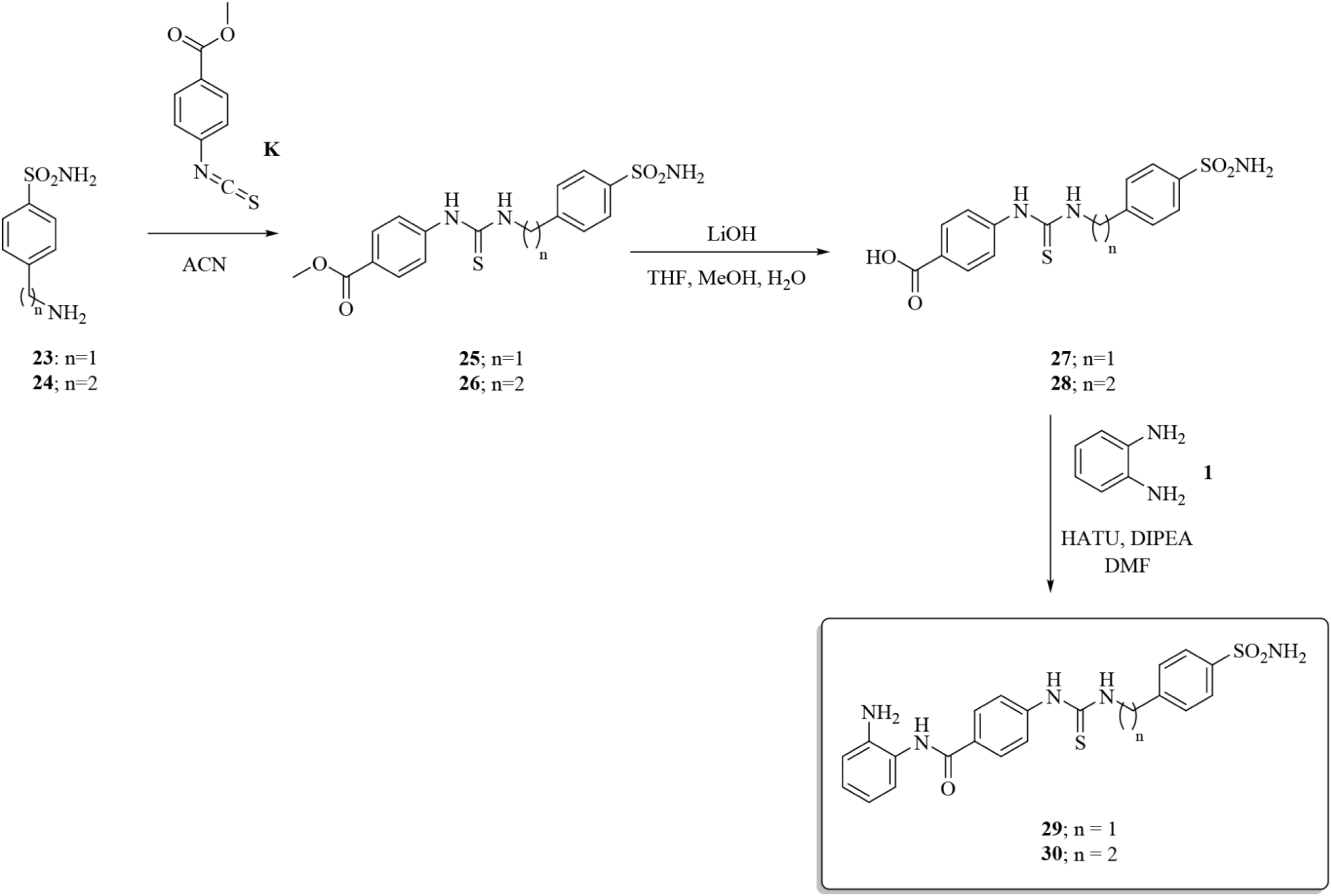
Synthesis of Elongated Thioureido Compounds **29** and **30**

In this case, the key intermediates **27** and **28** were obtained by the addition reaction of 4-aminoalkylbenzenesulfonamides **23** and **24** with the isothiocyanate **K** followed by hydrolysis of the methyl esters **25** and **26**.

Among our molecular elongation approaches, we explored the insertion of natural amino acids, and simple glycine was assumed to be an ideal model for such purposes (Scheme 7).

**Scheme 7:**
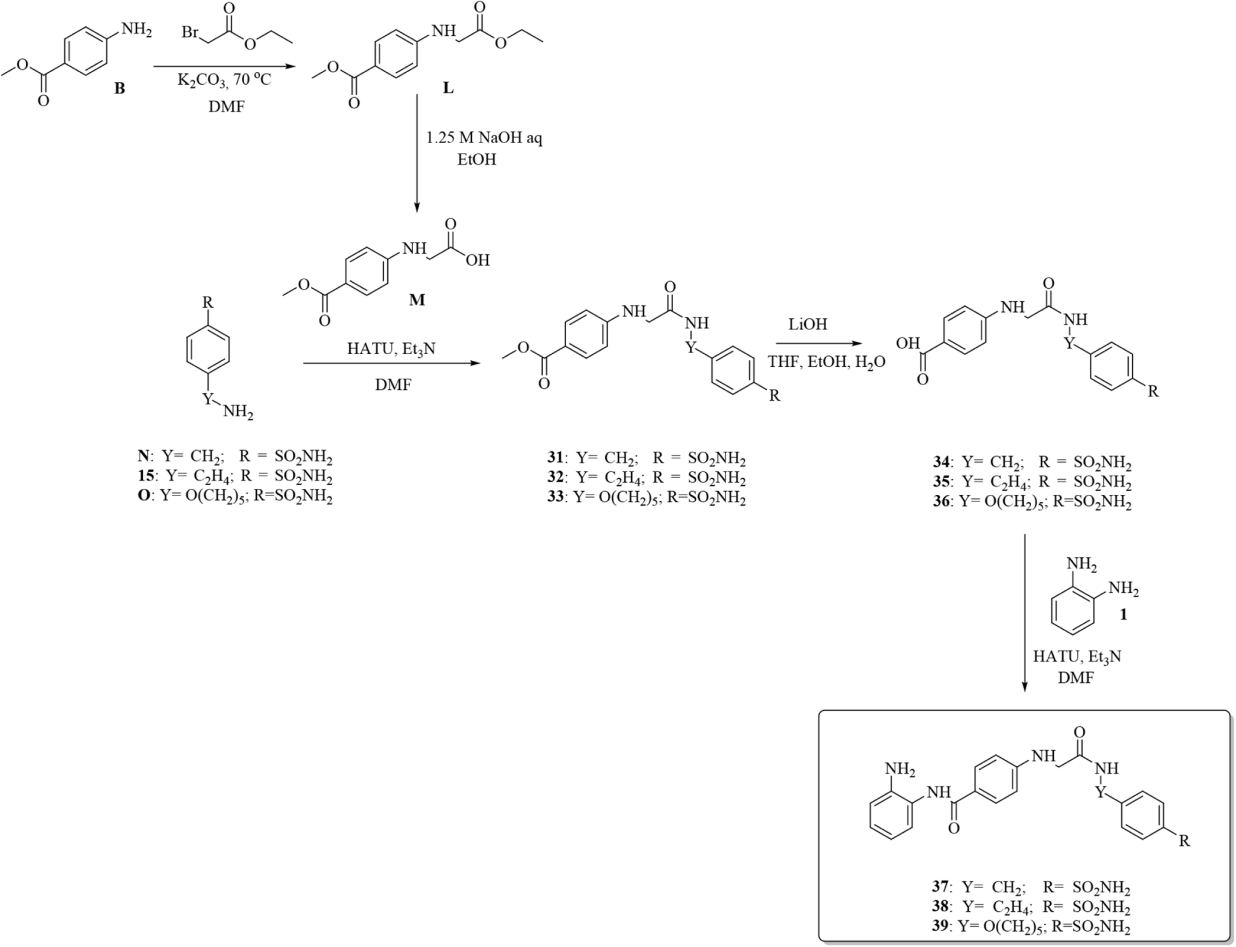
Synthesis of Glycine-Containing Compounds **37**, **38**, and **39**

The glycine key fragment **M** was readily obtained by the reaction of ethyl 2-bromoacetate with methyl 4-aminobenzoate **B**, followed by regioselective hydrolysis of the aliphatic ester in **L** with 1.25 M aqueous NaOH in ethanol. The primary sulfonamide-containing moieties **N** and **O** were coupled to **M** by making use of their free primary amine pendant, thereby affording **31**, **32**, and **33,** respectively. The remaining synthetic steps to obtain compounds **37–39** were carried out according to previously reported procedures (i.e., ester hydrolysis and *o*-aminoaniline coupling).

All the final compounds reported herein were purified by silica gel column chromatography using the appropriate eluting mixtures, followed by trituration or recrystallization as needed (see Section 3). Full characterization was conducted in solution ^1^H-, ^19^F-, and ^13^C-NMR. Elemental analyses confirm a purity grade ≥96%.

### 2.2 *In Vitro* Carbonic Anhydrase Inhibition Assay

The inhibition profiles for the final compounds and the reference CAI drug acetazolamide (**AAZ**) were experimentally determined by means of the stopped-flow CO_2_ hydrase assay^55^ on the physiologically relevant hCAs I, II, IX, and XII, as reported in Table 1.

**Table 1:** Inhibition Data of hCAs I, II, IX, and IX with Compounds **4, 5, 8, 11, 12, 14, 18, 21, 22, 29, 30, 37–39** and the Standard Sulfonamide Inhibitor Acetazolamide (**AAZ**) by a Stopped-Flow CO_2_ Hydrase Assay^55^

*K*_i_ (nM)^a^				
Compound	hCA I	hCA II	hCA IX	hCA XII
**4**	17.7	3.7	29.3	8.4
**5**	2447	248.0	31.4	8.6
**8**	131.4	12.9	27.5	20.2
**11**	144.6	7.4	31.0	7.3
**12**	533.8	7.7	28.2	8.1
**14**	553.6	7.2	30.8	7.5
**18**	79.4	19.9	29.2	6.9
**21**	97.7	9.4	29.0	9.3
**22**	426.0	88.4	28.6	9.2
**29**	59.7	6.7	28.4	61.4
**30**	63.7	9.1	105.9	67.9
**37**	8537	82.3	18.4	12.5
**38**	7235	59.9	15.7	9.0
**39**	53.9	6.2	31.7	74.7
**AAZ**	250.0	12.1	25.8	5.7

a Mean from 3 different assays by a stopped-flow technique (errors were in the range of ±5–10% of the reported values; data not shown).

Overall, the final compounds were effective inhibitors of the enzymatic panel considered and, except for a few cases, were far more potent than the reference **AAZ**.

Based on the inhibition data in Table 1, the following structure–activity relationships (SARs) may be drawn:

i) The linear and shortest HDAC–CA derivative **4** was the most potent inhibitor of hCA I isoform among all the compounds in the series, with a *K*_I_ value of 17.7 nM. Switching of the warhead primary sulfonamide from position 4 to 3 on the phenyl ring, along with the introduction of a chlorine atom, strongly reduced the inhibition potency by up to 138.2-fold (*K*_I_ of 2447 nM for compound **5**). Linear elongation of **4** by inserting a *para*-disubstituted phenyl spacer, as in compound **8**, also diminished the inhibitory potency against hCA I, although the effect was less pronounced (i.e., 17.7 and 131.4 nM for **4** and **8**, respectively). Interestingly, the Entinostat-like tail connected to the CA warhead by means of the ureido moiety (i.e., compound **11**) did not significantly affect the ligand’s inhibition potency when compared to **3** (i.e., *K*_I_ of 144.6 and 131.4 nM for **11** and **8**, respectively). Predictably, a strong regioisomeric effect occurred when the sulfonamide was moved to position 3, resulting in a decrease in the inhibitory potency (i.e., compounds **12** and **14** in Table 1). Both of these *meta*-substituted derivatives exhibited closely matching *K*_I_ values (i.e., 533.8 and 533.6 nM for **12** and **14**, respectively) and were up to 3.7-fold less potent compared to the *para*-disubstituted isomer **11**. Such data also indicated that the contribution of the fluorine atom inserted in **14** was not significant in inducing *in vitro* kinetic changes. Manipulation of **11** by elongating the aliphatic chain by a single carbon unit and translocating the ureido moiety toward the benzamide tail-end restored the inhibition potency to medium-range nanomolar *K*_I_ values (compare **11** and **18** in Table 1).

A strong regioisomeric effect on *in vitro* hCA I kinetics was clearly detected for compounds **21** and **22** bearing the thioureido spacer between the two enzymatically directed functionalities. In this case, the *meta*-substituted analog proved less effective in inhibiting hCA I (i.e., 4.4-fold) when compared to its regioisomeric *para* counterpart (*K*_I_ of 97.7 and 426.0 nM for **21** and **22**, respectively). Elongation of compound **21** by a methylene or ethylene spacer to afford **29** and **30**, respectively, restored the inhibition potency to medium nanomolar values. The hCA I inhibition values for **29** and **30** clearly showed no significant differences between the two ligands. A slight but significant discrepancy was observed for compounds **18** and **30**, which differ in the presence of the ureido and thioureido moieties, respectively.

Insertion of the methylglycine spacer, as in **37**, significantly reduced the hCA I inhibition potency (*K*_I_ = 8537 nM). Interestingly, the elongation of the alkyl tether facing the CA warhead, as in compound **38**, resulted in a 1.18-fold reduction of the *K*_I_ value (i.e., *K*_I_ = 7235 nM), which was further reduced when the ether moiety was introduced, yielding the second most potent hCA I inhibitor **39** within the series (*K*_I_ = 53.9 nM).

ii) As for the housekeeping-abundant hCA II, overall, the compounds showed more potent inhibition features when compared to those of the hCA I isozyme. Again, compound **4** was the most potent in the series, with a *K*_I_ value of 3.7 nM, thus being 3.3-fold more potent than reference **AAZ** (*K*_I_ of 12.1 nM). In analogy to hCA I, in this case, a strong regioisomeric effect was also reported when the primary sulfonamide was moved to the *meta* position (i.e., compare **4** and **5** in Table 1). Enhancement of the *K*_I_ value by up to 3.5-fold was also reported when the phenylacetamido spacer was introduced in **4** to afford compound **8** (*K*_I_ of 12.9 nM). Interestingly, the presence of the methylene ureido moiety, as in **11** and **12**, endowed the ligands with potent inhibition potencies against the hCA II isozyme, with suppression of any regioisomeric effect (*K*_I_ of 7.4 and 7.7 nM, respectively), as previously shown for hCA I. A slight decrease in the *K*_I_ value was obtained when the fluoroanilido tail was introduced instead (*K*_I_ of 7.2 nM for compound **14**). Comparison of the *meta*-regioisomers **12** and **14** *K*_I_ values clearly indicated that no significant contribution to the inhibition potencies could be ascribed to the fluorine atom (i.e., *K*_I_ of 7.7 and 7.2 nM for **12** and **14,** respectively).

The thioureido ethyl linker in compound **30** proved particularly effective in gaining hCA II inhibition potency when compared to its ureido bioisostere **18** (*K*_I_ of 9.1 and 19.9 nM for **18** and **30,** respectively), which, in turn, resulted in 1.4-fold less effective when compared to the shortest methylene-containing derivative **14** (*K*_I_ of 6.7 nM). A marked regioisomeric effect was observed for compounds **21** and **22**, with the *meta*-substituted analogs being 9.4-fold less potent inhibitors when compared to the *para* counterpart (*K*_I_ of 9.4 and 88.4 nm for **21** and **22**, respectively). Derivatives **37–39** showed a kinetic trend for hCA II comparable to that of hCA I. As reported in Table 1, elongation of the methylene glycine up to the insertion of the ether moiety resulted in a progressive enhancement of inhibition potency (*K*_I_ of 82.3, 59.9, and 6.2 nM for **37**, **38**, and **39**, respectively).

**Table 2:** Inhibition Data of HDACs 1, 3, 4, 6, and 8 with Compounds **4, 5, 8, 11, 12, 14, 18, 21, 22, 29, 30, 37–39** Compared to Reference HDAC Inhibitors **Trichostatin A** (**TSA**) and **TMP 269** ^a^ ^b^

	IC_50_ (μM)^a^				
Compound	HDAC1	HDAC3	HDAC4	HDAC6	HDAC8
**4**	>200	1.85	>200	>200	50.5
**5**	>200	32.6	>200	>200	>200
**8**	>200	0.73	>200	>200	64.7
**11**	>200	0.21	>200	>200	3.60
**12**	>200	0.15	>200	>200	3.77
**14**	>200	0.19	>200	>200	27.0
**18**	>200	0.54	>200	>200	7.79
**21**	>200	0.86	>200	>200	14.3
**22**	>200	0.95	>200	>200	17.8
**29**	>200	1.26	>200	>200	10.2
**30**	>200	0.83	>200	>200	12.0
**37**	0.18	1.66	NA	NA	85.9
**38**	0.34	1.44	>200	>200	83.1
**39**	>200	1.81	>200	>200	14.3
**TSA**	0.018	0.024	ND^b^	0.004	0.619
**TMP 269**	ND	ND	0.239	ND	ND

a Mean from 3 different assays.

b ND, not determined.

iii) As a general overview, the *in vitro* kinetic data referring to the tumor-associated hCA IX were all in the medium nanomolar range, with minimal differences between the screened compounds (Table 1). For instance, the significant regioisomeric effect observed for compounds **4** and **5** on the widely expressed hCA I/II was almost suppressed when the hCA IX isoform was considered instead (*K*_I_ of 29.3 and 31.4 nM were observed for **4** and **5**, respectively). The introduction of the phenylacetamide (i.e., compound **8**) or ureido methylene (i.e., compounds **11**, **12**, and **14**) moieties as tethers did not significantly impact the hCA IX inhibition potency of such compounds (i.e., *K*_I_ comprised between 27.5 and 31.0 nM). Noteworthy is the striking detrimental effect on the inhibition potency caused by the thioureido moiety in **30** over its ureido counterpart **18** (i.e., *K*_I_ of 105.9 and 29.2 nM for **30** and **18,** respectively), which was restored to the original value when the methylene alkyl spacer was introduced instead (*K*_I_ of 28.4 for compound **29**). In analogy to compounds **4** and **5**, the regioisomeric effect was also suppressed for derivatives **21** and **22**, which showed closely matching *K*_I_ values (i.e., *K*_I_ of 29.0 and 28.6 nM for **21** and **22**, respectively). Finally, the kinetic trend for the glycine-containing compounds **37–39** on hCA IX was also peculiar and thus not comparable to those of the previously discussed hCAs I and II (see Table 1 and discussion above). Single carbon unit elongation of **37** to afford the derivative **38** induced a 1.2-fold enhancement of the inhibitory potency (*K*_I_ of 18.4 and 15.7 nm for **37** and **38,** respectively). The insertion of the phenylether, as in compound **39**, spoiled the inhibition potency, making it the second least potent hCA IX ligand among the series (*K*_I_ of 31.7 nM).

iv) The set of compounds screened was particularly effective against the second tumor-associated hCA XII isozyme, showing most of the *K*_I_ values in the low nanomolar range (Table 1). In analogy to the hCA IX, also in this case, any regioisomeric effect on the kinetic values for **4**/**5** and **21**/**22** was suppressed (see Table 1). More importantly, both couples retained close *K*_I_ values despite their spacers being structurally different (i.e., *K*_I_ of 8.4 and 8.6 for **4**/**5**; *K*_I_ of 9.3 and 9.2 for **21**/**22**). The elongation of **4** with the phenylacetamido linker drastically reduced the inhibition potency by 2.4-fold (i.e., compare **4** with **8** in Table 1). A disconnection between the inserted tethers and the kinetic values for the HDAC–CA dual hybrids was also observed among the methylene ureido (**11**, **12**, and **14**) and ureidoethyl (**18**) derivatives, as they showed *K*_I_ values spanning between 6.9 and 8.1 nM. In analogy to the hCA IX, the isosteric substitution of the ureido moiety in **18** with the thioureido instead, as in **30**, resulted in a significant increase in the *K*_I_ value (i.e., 9.8-fold), which was only slightly reduced when the alkyl tether was shortened (i.e., *K*_I_ of 67.9 and 61.4 nM for compounds **30** and **29**, respectively). A slightly enhanced hCA IX inhibition potency gain (1.4-fold) was obtained when the *N*-ethyl glycine spacer replaced the *N*-methyl one (see compounds **37** and **38** in Table 1). Again, the introduction of the *O*-ether moiety, as in compound **15**, markedly increased the *K*_I_ value to 74.7 nM.

### 2.3 *In Vitro* HDAC Inhibition Assay

The final compounds were also screened on human recombinant HDACs 1, 3, 4, 6, and 8 to assess their capability to inhibit some representative members of deacetylases. The new compounds were tested against the selected HDAC isoforms in singlet 10-dose mode with 3-fold serial dilution starting from a 200 μM solution. Inhibition values for each tested HDAC isozyme were measured with a homogeneous fluorescence release HDAC assay. Purified recombinant enzymes were incubated with the serially diluted inhibitors at the indicated concentrations. The deacetylase activities of HDACs 1, 3, 4, 6, and 8 in the presence of serial dilutions of the final compounds were determined by assaying enzyme activity using the p53 residues 379–382 (Arg-His-Lys-Lys­(Ac)­AMC) to detect inhibitory activity against HDACs 1, 3, and 6; the diacetylated peptide from p53 residues 379–382 (Arg-His-Lys­(Ac)-Lys­(Ac)­AMC) to detect inhibition against HDAC8; and the fluorogenic class IIa (Boc-Lys­(trifluoroacetyl)-AMC) substrate to detect inhibition against HDAC4.^56^

All of the evaluated compounds exhibited inhibitory activity against HDAC3 and HDAC8, ranging from submicromolar to single/dual-digit micromolar levels. Compound **4**, which was derived from the direct merging of both HDAC and CA-inhibiting pharmacophores, displayed HDAC3-selective inhibition, with a 27-fold selectivity over HDAC8 (IC_50_ = 1.85 μM vs IC_50_ = 50.5 μM). Structural modification of **4**, involving a switch of the sulfonamide group from the *para*- to the *meta*-position, provided regioisomer **5**. This structural modification, combined with the introduction of a 4-chloro substituent on the benzenesulfonamide moiety, resulted in a reduction in HDAC3 inhibitory potency (IC_50_ = 32.6 μM) and a complete loss of HDAC8 inhibitory activity. Subsequently, extending the distance between the two pharmacophores by inserting an additional benzamide moiety led to compound **8**, which exhibited a moderate improvement in HDAC3 inhibition (IC_50_ = 0.73 μM). Notably, when a ureido moiety group was introduced in the linker, an enhancement of the inhibitory potency against HDAC3 was observed, with compound **12** being the most potent in the developed series (**11**, **12**, **14**, **18**), with an IC_50_ value of 0.15 μM and a 24-fold selectivity over HDAC8. The shifting of the sulfonamide group from the *meta*- (**12**) to the *para*-position (**11**) gave a similar potency toward both HDAC3 and HDAC8. Moreover, when a fluorine atom was introduced on the 2’-aminoanilide moiety of **12**, the resulting compound **14** displayed the best selectivity profile among all the tested compounds, exhibiting HDAC3 selectivity of 140-fold over HDAC8, although a slight drop in inhibition potency against HDAC3 was observed (IC_50_ = 0.19 μM, **14** vs 0.15 μM, **12**).

By both inverting and extending the ureidomethylene group to the ureidoethylene one, as in compound **18**, a slight reduction in HDAC3 inhibitory activity (IC_50_ = 0.54 μM) compared to its analogue **11** was observed. The substitution of the ureido group with the thioureido group, as seen in compounds **21**, **22**, **29**, and **30**, also reduced HDAC3 inhibitory potency. Within this subset, variations in sulfonamide regioisomerism (compounds **21** and **22**) and the distance between the thioureido and benzenesulfonamide moieties (compounds **29** and **30**) did not significantly affect HDAC3 inhibition.

As a final exploration, the ureido group was replaced by the glycineamide group (**37–39**), and notably, both compounds **37** and **38** maintained single-digit micromolar HDAC3 inhibition but acquired inhibitory potency toward HDAC1, with compound **37** being the most potent (IC_50_ = 0.18 μM). However, when the distance between the glycineamide and the benzenesulfonamide moieties increased (compound **39**), gain of inhibition potency and selectivity for HDAC3 was observed.

### 2.4 Target Specificity Assessment

In order to assess the specificity of each moiety contained in our compounds toward the enzymatic targets considered in this study (i.e., hCAs and HDACs), we synthesized derivatives of the compound **11** devoid of the *o*-aminoanilide (i.e., **11a**) or the primary sulfonamide (i.e., **11b**) moiety, respectively (see Figure 4, Schemes S2 and S3 in Supporting Information file), and we profiled these compounds *in vitro* on the chosen hCA and HDAC isoforms (Tables 3 and 4). Since compound **11** is highly effective against hCA II, the tumor-associated isoforms IX and XII, and HDAC3, structural modifications leading to derivatives **11a** and **11b** are expected to significantly impact these biochemical targets, and these modifications may help clarify the contribution of each moiety in the inhibition of the selected enzymatic isoforms.

**Figure 4:**
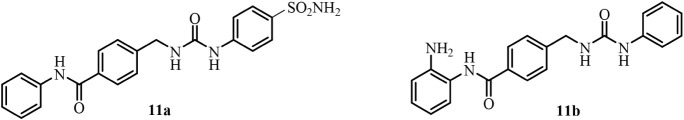
Chemical structures of compounds **11a** and **11b**.

**Table 3:** Inhibition Data of hCA I, II, IX, and XII with Compounds **11a** and **11b**, the Standard Sulfonamide Inhibitor Acetazolamide (**AAZ**) and the Reference Compound **11** by a Stopped-Flow CO_2_ Hydrase Assay^55^

	*K*_r_ (nM)^a^			
Compound	hCA I	hCA II	hCA IX	hCA XII
**11a**	2267.7	11.5	122.2	57.6
**11b**	>100 000	>100 000	>100 000	>100 000
**11**	144.6	7.4	31.0	7.3
**AAZ**	250.0	12.1	25.6	5.7

a Mean from 3 different assays by a stopped-flow technique (errors were in the range of ±5–10% of the reported values; data not shown).

**Table 4:** Inhibition Data of HDACs 1, 3, 4, 6, and 8 with Compounds **11a** and **11b** Compared to Compound **11** and the Reference HDAC Inhibitors **Trichostatin A** (**TSA**) and **TMP 269** ^a^ ^b^

	IC_50_ (μM)^a^				
Compound	HDAC1	HDAC3	HDAC4	HDAC6	HDAC8
**11a**	>200	>200	>200	>200	>200
**11b**	0.05	0.16	>200	>200	24.0
**11**	>200	0.21	>200	>200	3.60
**TSA**	0.018	0.024	ND^b^	0.004	0.619
**TMP 269**	ND	ND	0.239	ND	ND

a Mean from 3 different assays.

b ND, not determined.

As reported in Tables 3 and 4, the presence of only the primary sulfonamide, as in **11a**, determined exclusive inhibition of the hCAs with preferential activity for the secondary tumor-associated hCA XII and the cooperative isoform II (i.e., *K*_I_ of 57.6 and 11.5 nM for hCA XII and II, respectively). In addition, all *K*_I_ associated values for **11a** were far higher when compared to the parent **11**. Removal of the CA-directed scaffold in **11** and insertion of the *o*-aminoanilide, as in **11b**, did not interfere with the hCA tested (i.e., *K*_I_ > 100 000 nM) and resulted in the inhibition of only HDACs 1, 3, and 8, as expected. Specifically, **11b** was particularly effective on HDAC1 (IC_50_ 0.05 μM), followed by the isoforms HDAC3 and HDAC4, with IC_50_ values of 0.16 and 24 μM, respectively.

Data reported above are robust in demonstrating that the two chemical moieties directed toward the HDACs and hCAs are independent of each other, and no cross-activity between them is expected to occur when considered in more advanced studies.

### 2.5 X-ray Crystallography

The main interactions occurring between hCA II and compounds **37** and **38** were determined by X-ray experiments of the corresponding adducts, both at 1.43 Å resolution (Figure 5A,B and Table S1 in the Supporting Information file). Overall inspection of the electron density maps accounted for **37** and **38** deep buried within the enzymatic cleft, with the primary sulfonamides anchored to the zinc metal ion and according to the canonical binding cluster typical for the α-CAs (Figure S1 in Supporting Information file).^57^ In addition, the phenyl rings loaded with the CAI moiety were engaged with Leu198 and Val121 through van der Waals interactions (Figure 5A,B).

**Figure 5:**
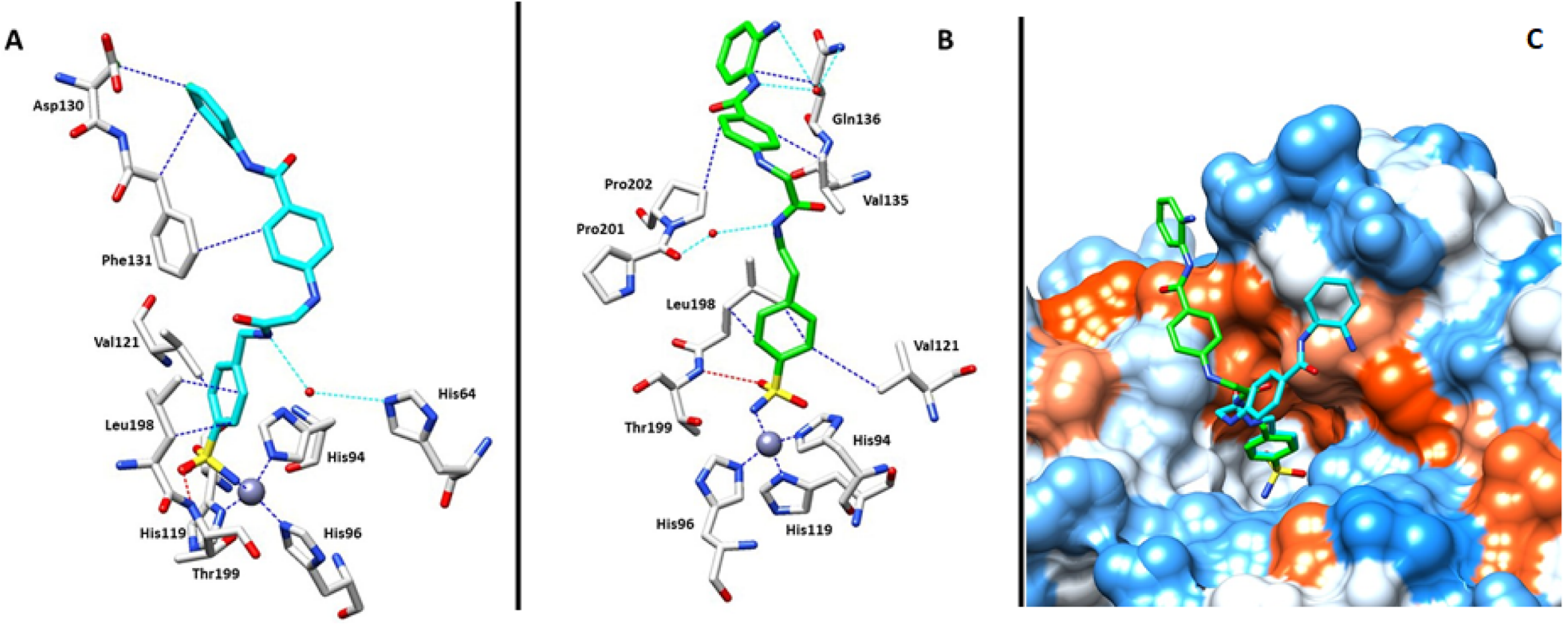
X-ray crystal structures of hCA II bound to **37** (**A** PDB: 7QSE) and **38** (**B** PDB: 7QRK). Residues involved in the binding of inhibitors are also shown: blue represents hydrophobic interaction, red represents hydrogen bond, cyan represents water bridge interaction, and the gray sphere represents the zinc atom in the active site of the proteins; (**C**) **37** (green) and **38** (cyan) overlaid in the active site of hCA II. Hydrophobic (red) and hydrophilic (blue) residues are reported.

Overlay of the hCA II/**37** and hCA II/**38** adducts showed both ligand tails lining the hydrophobic half of the enzyme and projecting their benzamide ends outside the cleft; however, distinct interactions within the enzymatic cleft drove the ligand tails toward divergent orientations (Figure 5C). The hCA II/**37** adduct showed that the CAI moiety’s proximal ureido nitrogen water bridged with the proton shuttle His64 residue. Such an interaction was missing for the longer **38**, as its additional carbon unit in the ethyl spacer pushed upward the corresponding nitrogen, which turned 90° and interacted with the Pro201–Pro202 carbonyl by means of a water molecule as well (Figure 5A–C). A series of hydrophobic interactions between Asp130 and Phe131 with the benzamidic phenyl rings in hCA II/**37** defined the binding mode of such a compound (Figure 5A). As for the tail in **38**, an intricate network of hydrophobic and hydrophilic interactions was established with Pro202, Val135, and Gln136 (Figure 5B).

#### 2.5.1 *In Silico* Binding Mode

Among the highly similar class I HDAC enzymes (i.e., HDAC1, 2, and 3), only the isoform HDAC2 in adduct with an *o*-amino benzamide derivative was characterized by means of X-ray crystallography, specifically with the compound **BRD6929** in Figure 6.^58^

**Figure 6:**
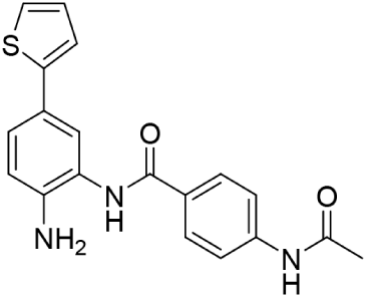
Chemical structure of **BRD6929** cocrystallized with HDAC2 (PDB: 4LY1).

Structural coordinates of HDAC1 (in complex with an acetate ion) and HDAC3 (bound to a corepressor and inositol) were superimposed onto those of HDAC2 using the MatchMaker command in UCSF Chimera.^59^ The modeled complexes **BRD6929**/HDAC1 (i.e., CPLX 1) and **BRD6929**/HDAC3 (CPLX 3), solvated with a 5 Å layer of explicit water molecules, were geometrically optimized through a single-point minimization with 2000 iterations using the UFF force field as implemented in Open Babel.^60^ The same geometry optimization was also applied to the 4LY1 complex. The rationale for **BRD6929** binding mode retention was based on the observation of the well-known inhibitors **Vorinostat** (SAHA) and **Trichostatin A** (TSA), which were complexed with several HDAC isoforms and displayed very similar binding conformations (Figure S2 in the Supporting Information file). The **BRD6929**/HDAC2 (4LY1, CPLX 2) complex was used to select the most suitable molecular docking application among a list of 15 programs (27 program/scoring function combinations, Table S2 in the Supporting Information file) as implemented in the www.3d-qsar.com portal.^61^ Despite many of the programs performing quite well, the combination of glamdock/glamdock_new_energy,^62^ PLANTS/PLP95,^63^ and LeDock^64^ gave the lowest RMSD values when compared to docked conformations generated from either experimental or randomized **BRD6929** conformations to the crystallographic pose of **BRD6929**/HDAC2 (Table S2 and Figure 7). A similar docking assessment was also performed for the modeled complexes CPLX 1 and CPLX 3. Interestingly, although there were slightly different RMSD values, the same three programs were also the best-performing ones (not shown).

**Figure 7:**
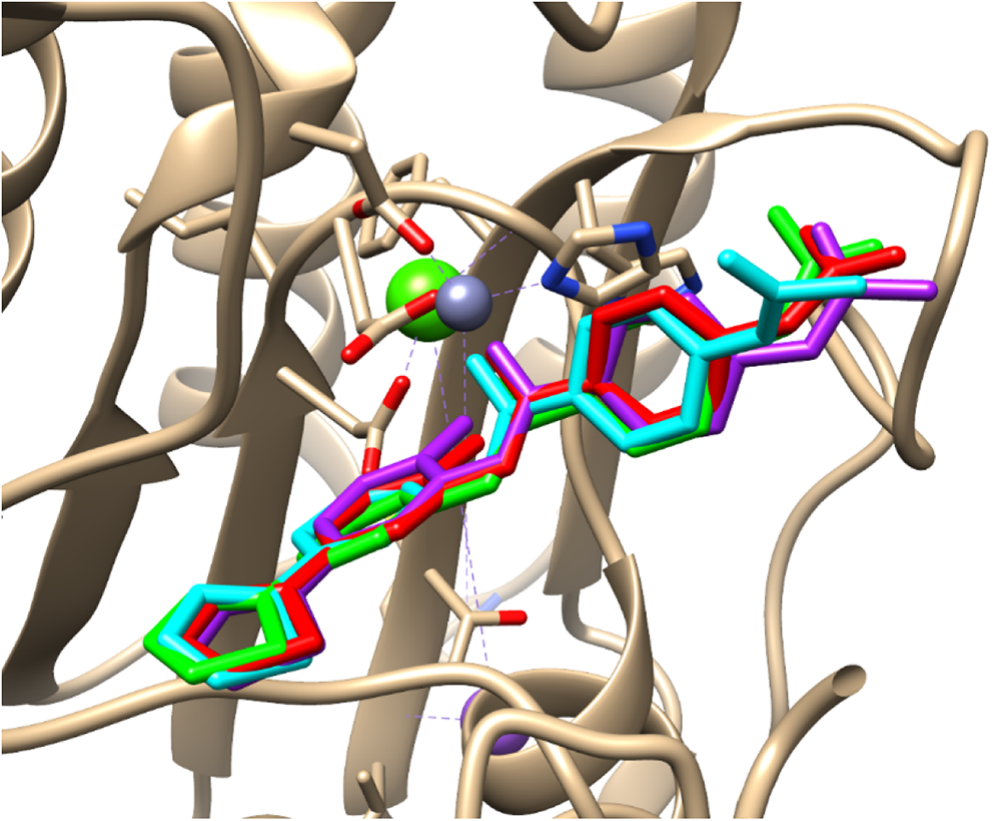
Overlapped redocked conformations of **BRD6929** into HDAC2 as proposed by GlamDock (red), PLANTS (green), and LeDock (cyan) programs. The experimental conformation is also displayed for comparison (purple).

The HDAC-selective compounds **11**, **12**, and **14** in Table 2 were assessed for their binding modes within such an isoform. Furthermore, compounds **37** and **38** in HDAC1 and HDAC3 were also docked to identify possible features differentiating their potency between the two isoenzymes. Visual inspection of the bound conformations proposed by the three selected molecular docking programs revealed comparable results; therefore, to reduce redundancy only the docked conformations proposed by the PLANTS/PLP95 combination were further inspected, and their respective binding modes were analyzed.

Regarding **11**, **12**, and **14** docked conformations into HDAC3, they all share similar geometries and binding interactions, as observed through the pose-view web application in Figure 8.^65^

**Figure 8:**
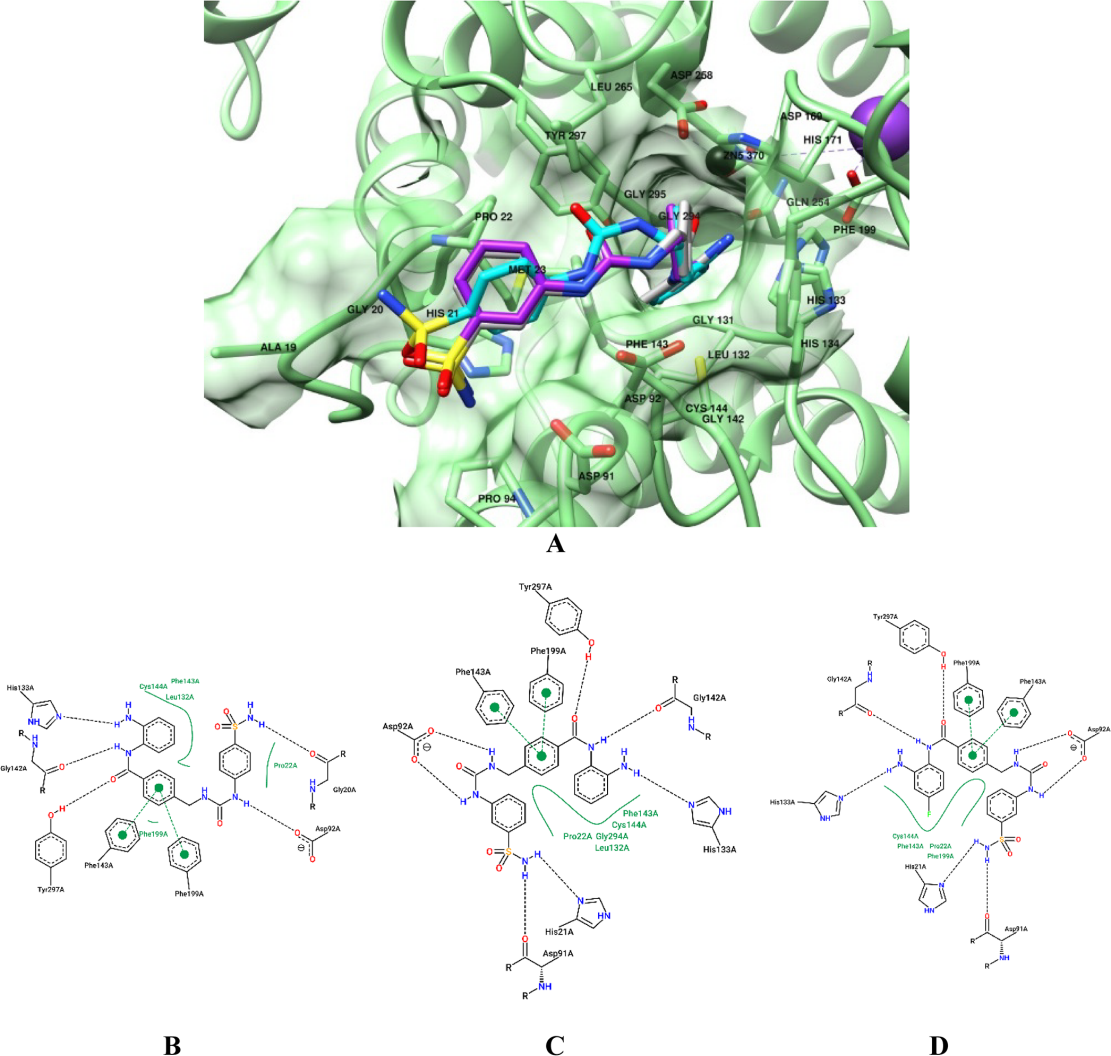
Bound conformations of **11** (cyan), **12** (purple), and **14** (gray) into HDAC3 (**A**) and PoseView diagrams for **11** (**B**), **12** (**C**), and **14** (**D**). Residue numbering could differ from the original 4LY1 complex due to residue renumbering through modeling. HDAC3 is depicted in green, the Zn^2+^ metal is depicted as a brown sphere, and hydrogen atoms are not displayed for the sake of clarity.

Specifically, the selected compounds established van der Waals interactions with the amino acid residues primarily located within the channel region of HDAC3 and include Pro22, Gly294, Phe143, and Cys144. An additional contact with Leu132 was detected only for compound **12** (Figure 8C). The central phenyl ring in **11**, **12**, and **14** established π–π interactions with Phe143 and Phe199. A network of hydrogen bonds was created between the ligands’ anilidic oxygen and Tyr297, the *o*-aniline NH and the ε-nitrogen of His133, and the anilidic NH and the carbonyl oxygen of Gly142. For derivative **11**, the distal NH of the ureido moiety interacted with the Asp92 carboxylic moiety, whereas the same amino acid was engaged in a double interaction with both ureido NHs in **12** and **14**. The *p*-substituted primary sulfonamide in **11** established a hydrogen bond with the Gly20 carbonyl oxygen, whereas its *m*-regioisomers **12** and **14** determined such group to interact with both the δ-nitrogen of His21 and the carbonyl oxygen of Asp91.^66^

Inhibitors **37** and **38** demonstrated selectivity for the HDAC1 isoform, with their associated IC_50_ values 4- and 9-fold lower, respectively, when compared to the second targeted isoform HDAC3 (Table 2). Such differences could, in part, be attributed to distinct binding modes assumed by the ligands within HDAC1 and HDAC3, despite the high sequence similarity shared between these two isoforms.^67^ A direct comparison of bound conformations revealed that **37** assumed two distinct binding modes in HDAC1 and HDAC3 (compare panels **A** and **B** in Figure 9). A deeper inspection revealed that **37** interacted within both isozymes with similar van der Waals contacts, and two additional hydrogen bonds contributed to better stabilizing its interaction with HDAC1 (compare panels **C** and **D** of Figure 9). The same profile was also observed for the investigational compound **40** (i.e., compound **38** devoid of its CA-directed moiety). The synthesis, chemical characterization, and *in vitro* enzymatic assessment on hCAs and HDACs of **40** are reported in the Supporting Information file and showed comparable inhibitory potency for HDAC1 (see Table 2 and **Tables** S3 and S4 in Supporting Information file). The reduced selectivity of compound **38** for HDAC1, compared to that of **37** and **40**, may be attributed to its distinct binding mode within this isoform. Specifically, **38** adopts a conformation that more closely resembles its binding geometry (Figure 9, panels **A** and **B**) and interaction profile (Figure 9, panels **E** and **F**) in HDAC3, potentially leading to greater cross-reactivity.

**Figure 9:**
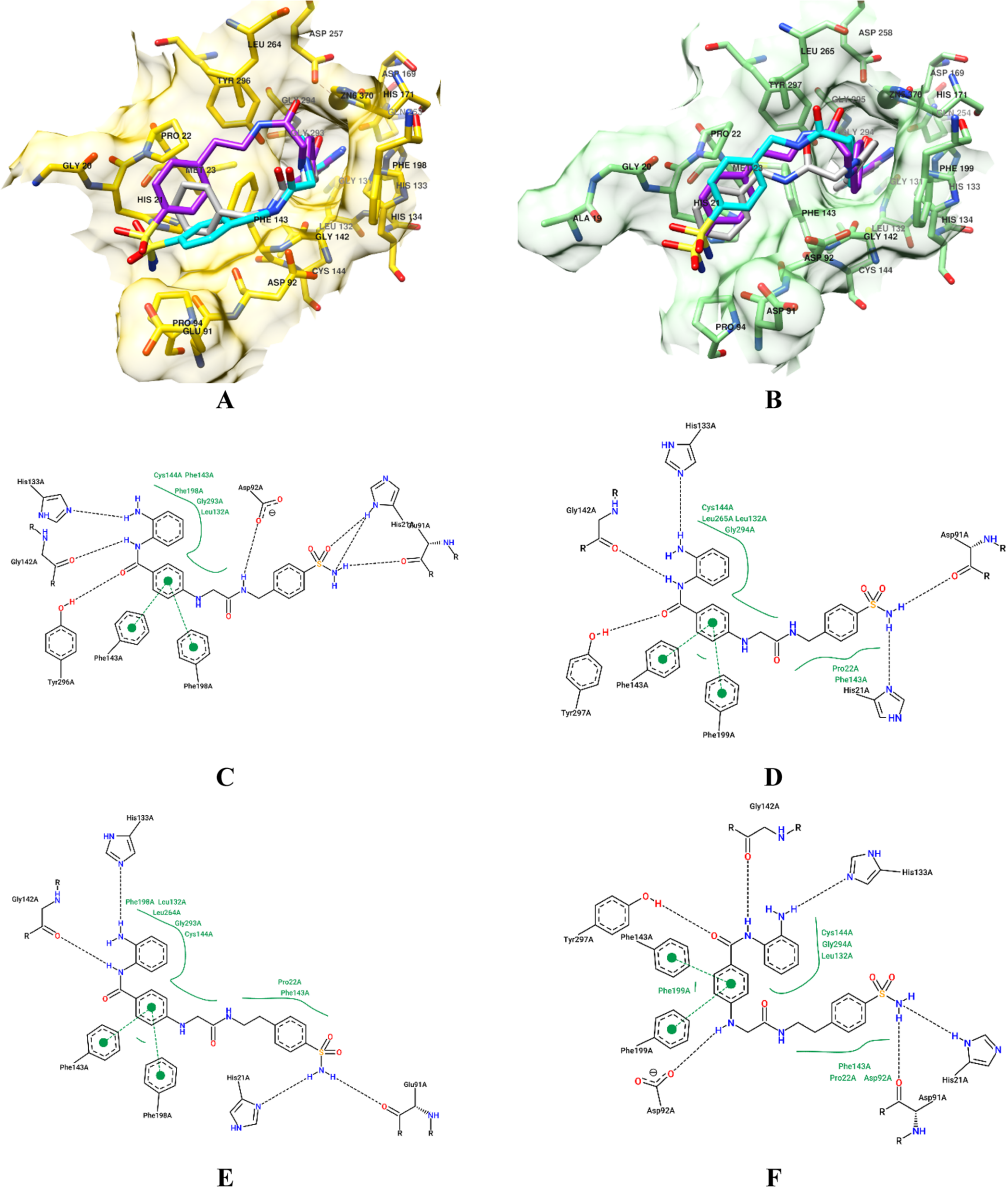
Binding conformations of **37** (cyan) and **38** (purple) into either HDAC1 (**A**) or HDAC3 (**B**). HDAC1 is depicted in yellow, and HDAC3 is depicted in green. Hydrogen atoms are not displayed for the sake of clarity. Compound **40** (gray) as a pure HDACI is also displayed for comparison purposes in panels **A** and **B**. PoseView diagrams for **37**/HDAC1 (**C**), **37**/HDAC3 (**D**), **38**/HDAC1 (**E**), and **38**/HDAC3 (**F**).

#### 2.5.2 *In Vitro* Cellular Assay

The better *in vitro* enzymatic inhibitors for both hCA IX and HDAC3 (i.e., compounds **11** and **14**) were tested *in vitro* for their ability to reduce the growth rates of tumor cells from different histotypes expressing aggressive phenotypes, such as the HCT-8 and HCT-116 colon carcinomas, the MDA-MB-231 and BT-474 mammary carcinomas, and the A375, 501Mel, and Sk-Mel-28 melanoma cells. All cell lines used in this study expressed a consistent level of hCA IX (Figure S5 in the Supporting Information file). Dose–response cell viability data were obtained using the MTT assay after exposing selected cells to increasing concentrations of **11** (Figure S6 in the Supporting Information file) and **14** (Figure S7 in the Supporting Information file) up to 72 h. Specifically, **11** was 2.4-fold more effective in reducing the cell proliferation of HCT-116 compared to HCT-8 colon carcinomas (i.e., IC_50_ values of 15.78 and 38.30 μM, respectively). A similar trend was reported for MDA-MB-231 and BT-474 cells, with associated IC_50_ values of 37.20 and 70.27 μM, respectively. Distinct IC_50_s were reported for melanoma cells, as **11** was barely effective on A375 (IC_50_ of 39.79 μM), whereas a 2.8- and 2.1-fold potency enhancement was observed when screened on 501Mel (IC_50_ 14.13 μM) and Sk-Mel-28 (IC_50_ 18.97 μM) cells (Figure S6 in Supporting Information file). Overall, derivative **14** showed lower performance when compared to **11** (Figure S7 in the Supporting Information file). For instance, the associated IC_50_ values for HCT-8 and HCT-116 colon carcinoma cell lines were 61.89 and 39.70 μM, respectively, which were more than double when compared to those of **11**. In addition, **14** was about 40% less effective than **11** on A375 melanoma cells (IC_50_ values of 62.37 and 39.79 μM for **14** and **11**, respectively). Interestingly, **14** and **11** showed similar antiproliferative effects when tested on mammary carcinoma cells (i.e., IC_50_ values of 43.53 μM in MDA-MB-231 and 61.07 μM in BT-474 cells) as well as on 501Mel and Sk-Mel-28 melanoma cells (i.e., IC_50_ values of 19.50 and 16.38 μM, respectively) (Figures S6 and S7 in Supporting Information file).

Derivatives **11** and **14** were further assessed for their toxicities on normal endothelial colony-forming cells (ECFCs) and gave IC_50_ values of 112.3 μM for **11** and 126.2 μM for **14** (Figure 10).

**Figure 10:**
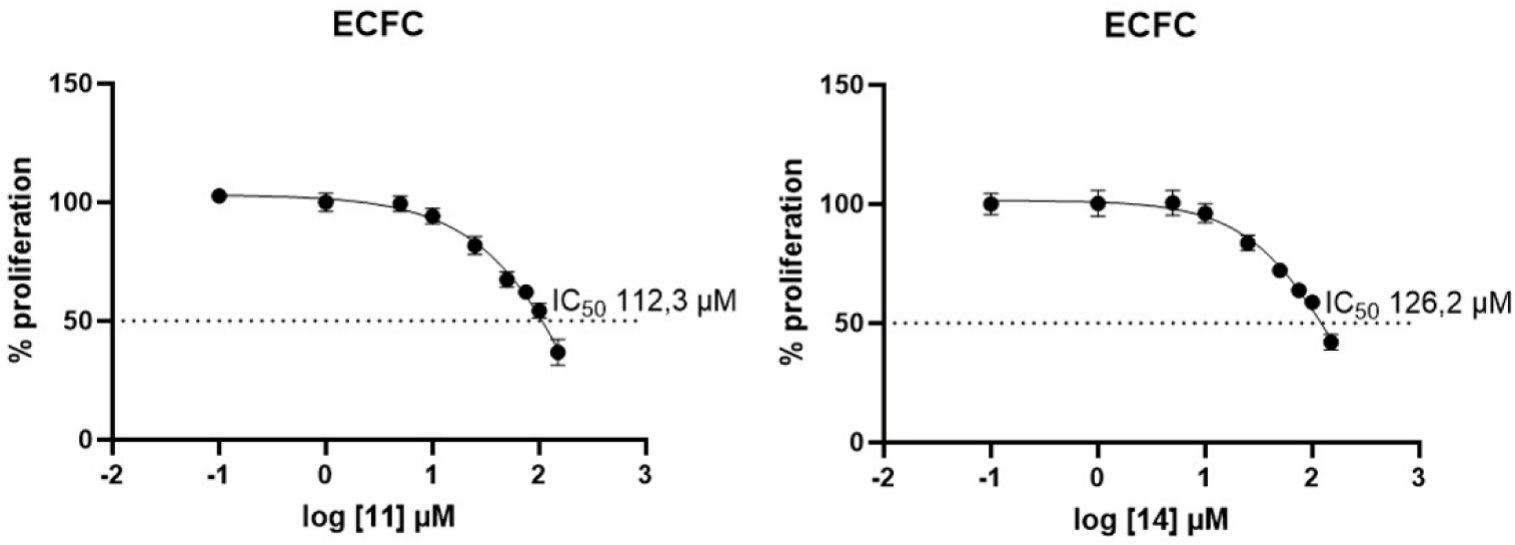
IC_50_ values of **11** and **14** on normal ECFCs. Dose–response curves were fitted using nonlinear regression (log­(inhibitor) vs response – variable slope, four-parameter logistic model) in GraphPad Prism 10.4.1. Data are shown as mean ± SD, *N* = 3.

The ECFC IC_50_ values were up to 5-fold higher than the corresponding tumor lines, thus reflecting the high selectivity of such compounds for tumoral cells over nontumoral ones. Such data represent the first evidence of the elevated translational value of **11** and **14** and were further consolidated when IC_50_ values for the clinically used HDAC inhibitor SAHA, screened on the panel of tumor cells selected in this study, were considered (Figure S8 in the Supporting Information file). Despite all IC_50_ values in Figure S8 being in the low micromolar range, it is relevant to note that **SAHA** was far more toxic than **11** and **14** on ECFCs (i.e., IC_50_ **SAHA** 9.85 μM vs IC_50_ **11** 112.30 μM and IC_50_ **14** 126.20 μM), thus confirming that the compounds reported here possess appreciable *in vitro* safety profiles and position themselves as good candidates for advanced *in vivo* studies.

To assess any significant advantage from hCA IX/HDAC3 dual inhibition, the highly water-soluble compound **11** was considered for its effects on tumor cell proliferation compared to its constitutive single chemical entities, which selectively target the enzymatic sites. The synthesis, chemical characterization, and *in vitro* enzymatic assessment of hCAs and HDACs for **11a** (i.e., compound **11** devoid of its HDAC-directed moiety) are reported in the Supporting Information file, whereas the commercially available compound **RGFP966** was used as a reference inhibitor for HDAC3. For the sake of clarity, the kinetic profile of **11a** on the hCA enzymatic panel matched with that of its progenitor **11**, and, as expected, it was devoid of any inhibitory activity on HDACs. Compound **11** was screened at its IC_50_ concentration on each cell line, and the same concentration was kept for **11a**. **RGFP966** was used at a 10 μM single dose and in combination with **11a**, and the proliferation data were determined by the MTT assay after 72 h of treatment (Figure 11).

**Figure 11:**
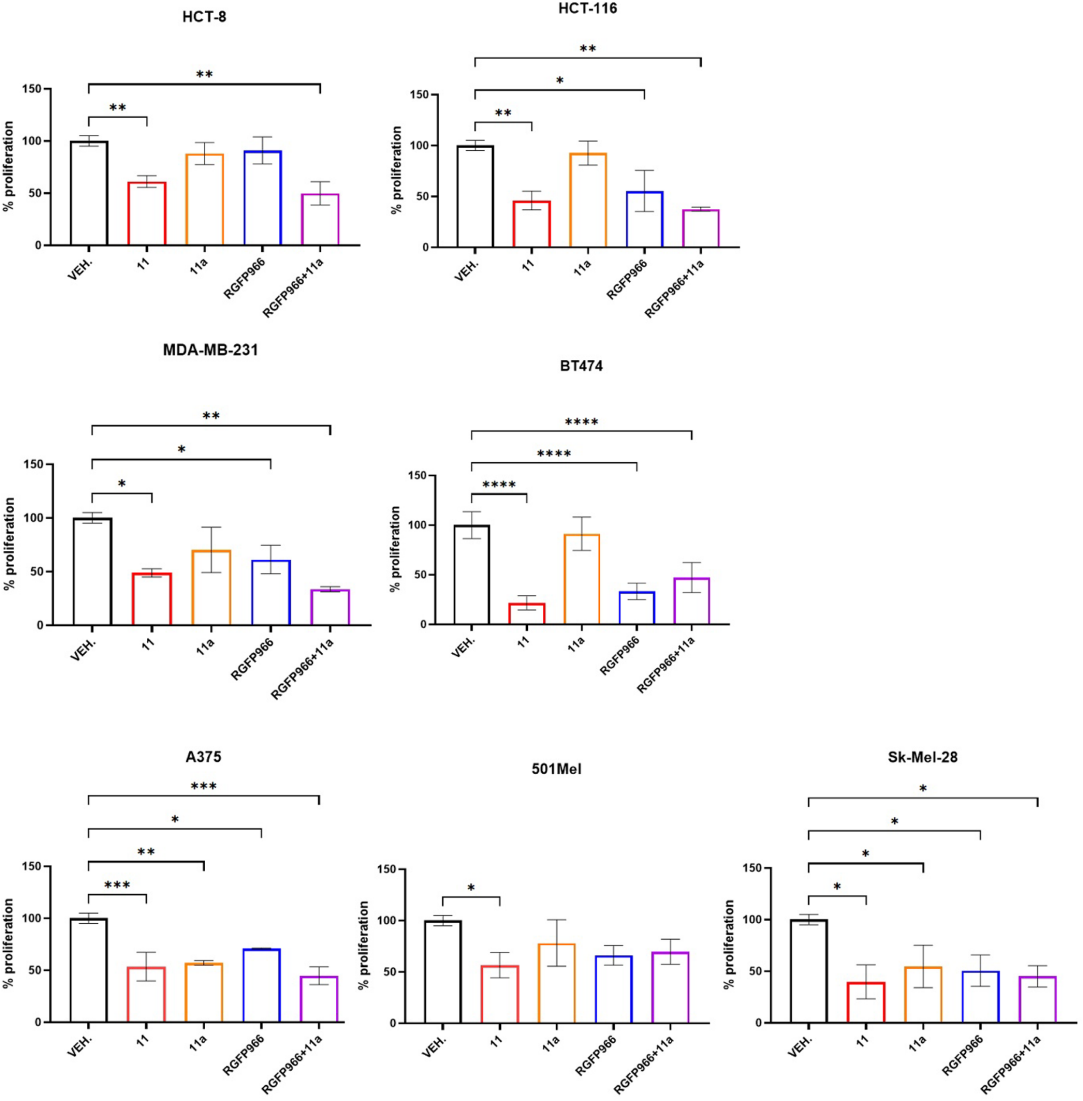
Representative plots of tumor cell proliferation after 72-h treatment with the IC_50_ dose of compound **11** or **11a**, or **RGFP966**. *p < 0.05, **p < 0.01, ***p < 0.001, ****p < 0.0001 vs vehicle (VEH.), *N* = 3. Two-way ANOVA, Dunnett’s multiple comparison test, GraphPad Prism 10.4.1.

A comparable reduction in the level of proliferation in tumor cells by compound **11** was observed when **11a** was combined with **RGFP966**. This was particularly evident in HCT-8 and HCT-116 colon carcinomas, as well as in MDA-MB-231 and BT-474 mammary carcinoma cells, as **11a** alone was not as efficient as **11** in inhibiting cell proliferation. In A375, 501Mel, and Sk-Mel-28 melanoma cells, the inhibition activities shown by **11**, **11a**, and **RGFP966** and the combined **RGFP966**/**40** were similar, with a tendency for **11** and **RGFP966**/**11a** to be more effective than **11a** or **RGFP966** alone. These data support the benefits of the dual inhibitory activity endowed by compound **11** in counteracting tumor cell proliferation.

Furthermore, the HCT-116 cell line was selected as a well-responsive tumor model to compound **11** (i.e., IC_50_ of 15.78 μM) to assess tumor cytotoxicity. As reported in Figure 12, cells were treated with an IC_50_ dose of compound **11** or **11a**, or 10 μM **RGFP966** alone, or in combination with **11a**. At 48 h of treatment, similar apoptotic levels of around 50% were observed with **11** and **11a** compounds, while about 70% cell death was obtained with **RGFP966** alone as well as in combination with **11a**. At 72 h, a higher tendency for cell death was observed upon treatment with compound **11** than **11a** alone, and a comparable apoptotic level was obtained with **RGFP966** treatment alone. The combined **RGFP966**/**11a** treatment was, instead, the most cytotoxic, reaching about 80% cell death within 72 h of treatment.

**Figure 12:**
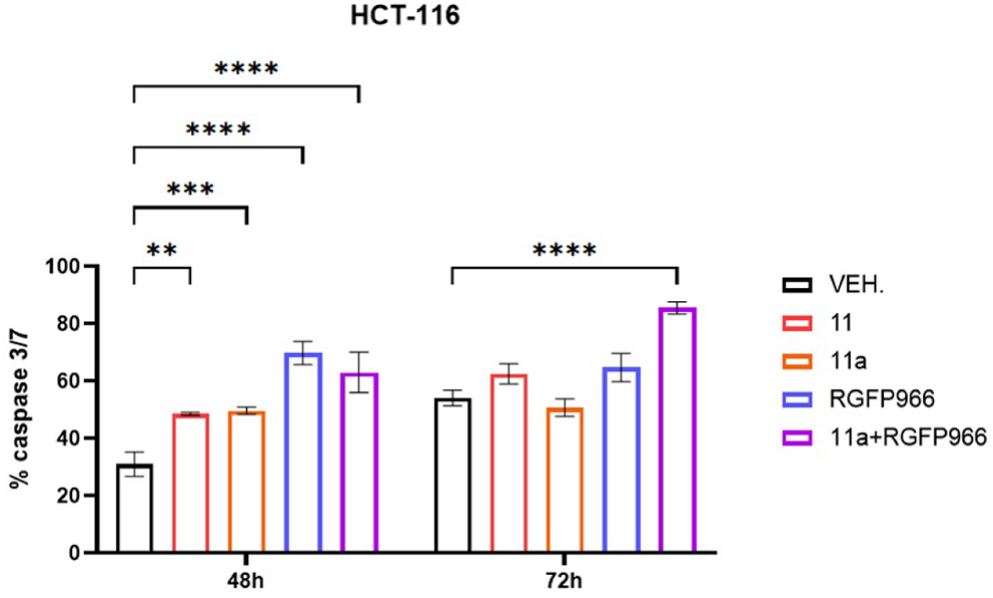
Representative plot of HCT-116 colon carcinoma cell death (active caspase 3/7) after 48-h and 72-h treatment with IC_50_ dose of compound **11** or **11a**, or 10 μM **RGFP966**. **p < 0.01, ***p < 0.001, ****p < 0.0001 vs vehicle (VEH.), *N* = 3. Two-way ANOVA, Dunnett’s multiple comparison test, GraphPad Prism 10.4.1.

The apoptotic HCT-116 cell death mechanism induced by **11** was assessed by the annexin V positive cells through flow cytometric analysis and showed a dose-dependent effect of up to ∼25% at a 25 μM concentration after 72 h of treatment (negligible effects were observed at 48 h) (Figure 13A).

**Figure 13:**
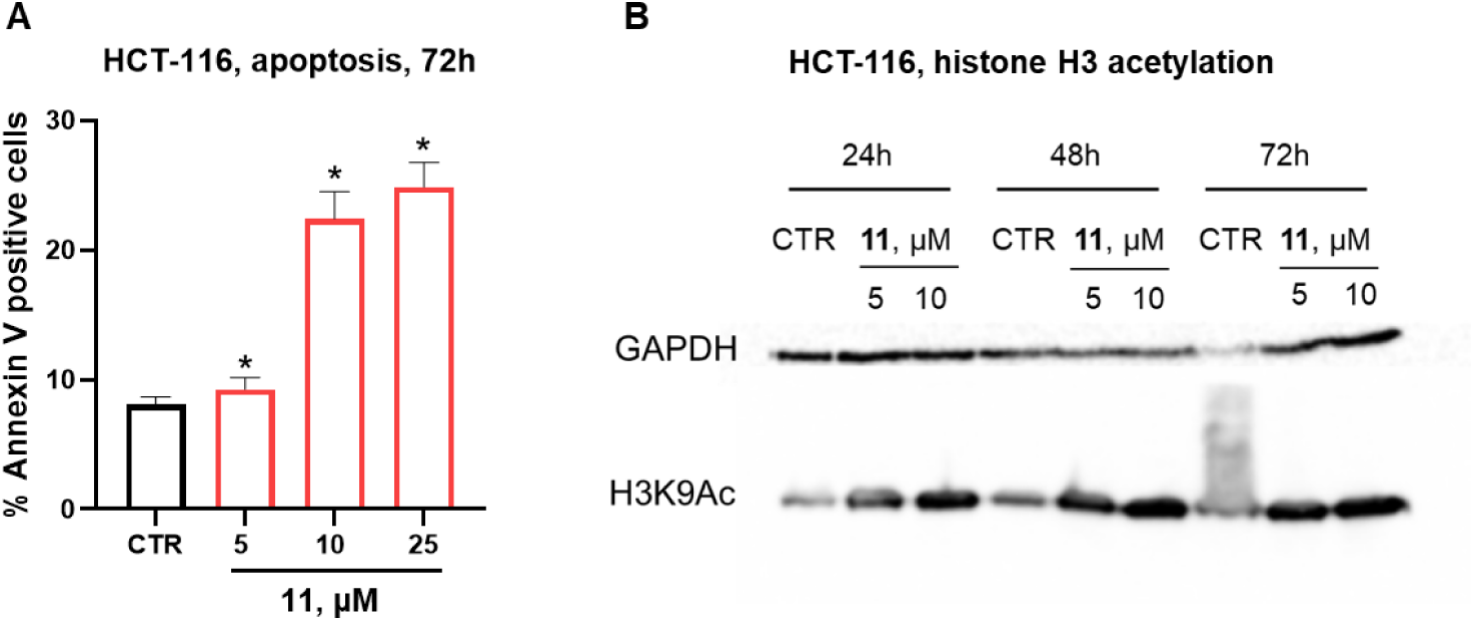
(**A**) Representative plot of HCT-116 colon carcinoma apoptosis (Annexin V) after 72-h treatment with 5, 10, and 10 μM doses of compound **11**; (**B**) Western blot analysis for H3K9 acetylation by compound **11** upon 5 and 10 μM treatment of HCT-116 at 24, 48, and 72 h. Apoptosis experiments were performed in triplicate ± SD (standard deviation). *p*-values were obtained using one-way ANOVA, *p < 0.05 vs control (CTR), GraphPad Prism 10.1.

In addition, the HDAC engagement from **11** in the same cell line was demonstrated by Western blot analysis for the acetylation levels of histone H3 at lysine 9 (Figure 13B). Specifically, compound **11** provided a significant time- and dose-dependent hyperacetylation effect detectable at 24 h of treatment, which is in agreement with the HDAC inhibition effect observed in cancer cells.^27–33^

#### 2.5.3 Conclusions

We designed and synthesized a series of previously non reported compounds consisting of a linear linkage connecting the prototypic CAI of the primary sulfonamide type with the *o*-aminoanilide moiety, which serves as the HDAC-directed warhead. *In vitro* experiments assessed the effectiveness of the obtained compounds in inhibiting the enzymatic activities of the intended targets with associated inhibition values comparable to the corresponding reference compounds and selectivity profiles favoring the tumor-associated isoforms. More importantly, the CAI and HDAC moieties retained target specificity, as no cross-activity between them was detected *in vitro* up to maximal concentrations. The binding modes of each moiety with respect to their corresponding targets were assessed by means of X-ray crystallography (i.e., for hCAs) and molecular modeling techniques (i.e., for HDACs). For instance, the binding of compounds **37** and **38** within the hCA II cavity was undoubtedly assessed at 1.43 Å resolution, and despite the canonical interaction of the primary sulfonamide,^57^ it is worth noting how a single carbon unit elongation dramatically affected the placement of the compound’s tail on the enzymatic rim. Significant data sets were also retrieved for the binding of selected compounds to HDACs 1 and 3. The best *in vitro* performing compounds toward the tumor-related isoforms hCA IX/HDAC3 (i.e., compounds **11** and **14**) showed a high ability to reduce the growth rates of various tumor cell lines *in vitro* and were far less toxic to normal endothelial colony-forming cells (ECFCs) even when compared to the clinically used HDAC inhibitor SAHA. The significant advantage of hCA IX/HDAC3 dual inhibition was assessed through combination experiments involving the highly water-soluble compound **11**, its HDAC-devoid counterpart **11a** and the commercially available HDAC-selective compound **RGFP966** on various tumor cell lines. Finally, the apoptotic effect induced from **11** was assessed on HCT-116, which is considered as the most responsive tumor model.

Overall, the authors are confident that the data reported in this study constitute robust scientific support for the definition of a first-in-class dual-acting hCA/HDAC-directed series of compounds, which have *in vitro* antiproliferative effects and safety profiles, and thus are entitled to further translational investigations.

## 3 Experimental Section

### 3.1 Materials and Methods

#### 3.1.1 Chemistry

Solvents and reagents were purchased from Alfa-Aesar, TCI, and Sigma-Aldrich. Reactions involving air- or moisture-sensitive chemicals were conducted under a nitrogen atmosphere by using oven-dried glassware, and the solutions were transferred via standard syringe techniques. Solution Nuclear Magnetic Resonance (NMR) spectra (i.e., 1H, ^13^C, and ^19^F) were recorded on a Bruker Avance III 400 MHz spectrometer using DMSO-*d*_6_ as the solvent. Chemical shifts (δ) were reported in parts per million (ppm) relative to residual solvent signals (i.e., 2.54 and 39.52 for ^1^H and ^13^C, respectively). Splitting patterns were designated as *s* (singlet), *d* (doublet), *t* (triplet), *m* (multiplet), *brs* (broad singlet), *dd* (doublet of doublets), and the corresponding coupling constants (*J*) were expressed in Hertz (Hz). Exchangeable protons (i.e., −OH and −NH) were identified by the addition of 2 drops of D_2_O to the DMSO-*d*_6_ solution. Thin-layer chromatography (TLC) was run on silica gel 60G plates with the fluorescent indicator F254 (Merck) and examined under UV light at 254 nm. Purification by column flash chromatography was executed with ethyl acetate/*n*-hexane as the eluents on silica gel 60G (230–400 mesh ASTM; Merck) as the stationary phase. High-Resolution Mass Spectrometry (HRMS) was performed on a Thermo Finnigan LTQ Orbitrap mass spectrometer coupled to an Electrospray Ionization Source (ESI). Analyses were carried out in positive ion mode [M + H]^+^ with a dwell acquisition time to achieve 60 000 units of resolution at full width at half maximum (fwhm). Elemental analyses were calculated on the basis of the accurate masses, accepting values with an error <5 ppm and a non-integer RDB (double bond/ring equivalents). Stock solutions of each compound were prepared using acetone as the solvent (1.0 mg/mL) and stored at 4 °C. Working solutions of each analyte were obtained by dilution using mQ H_2_O/ACN 1/1 (v/v) up to a final concentration of 1.0 μg/mL. Analyses were performed by introducing the working solution via syringe pump at a 10 μL/min flow.

#### 3.1.2 Synthesis of *N*-(2-Aminophenyl)-4-sulfamoylbenzamide (4) and *N*-(2-Aminophenyl)-4-chloro-3-sulfamoylbenzamide (5)

To a solution of *o*-aminoaniline **1** (1.0 equiv), 4-sulfamoylbenzoic acid (**2**) or 4-chloro-3-sulfamoylbenzoic acid (**3**) (1.0 equiv), and HATU (1.3 equiv) in dry DMF (3 mL/0.5 mmol), DIPEA or Et_3_N (2.0 equiv) was added under an inert atmosphere. The reaction mixture turned yellow and was stirred at room temperature overnight. The reaction mixture was then quenched with cold saturated NH_4_Cl solution and stirred for an additional 15 min, leading to the formation of a precipitate. The precipitated product was collected by vacuum filtration, washed with water, and triturated with diethyl ether (Et_2_O). The crude product was further purified by silica gel column chromatography using ethyl acetate (EtOAc) as the eluent, yielding the title compounds as a white powder.

#### 3.1.3 *N*-(2-Aminophenyl)-4-sulfamoylbenzamide (4)

Obtained according to the above procedure using *o*-aminoaniline (**1**) and 4-sulfamoylbenzoic acid (**2**). 78% yield; δ_H_ (400 MHz, DMSO-*d*_6_) 5.00 (2H, s, exchange with D_2_O, H*N*_2_), 6.64 (1H, t, *J* 7.2), 6.82 (1H, d, *J* 7.2), 7.02 (1H, t, *J* 7.2), 7.21 (1H, d, *J* 7.2), 7.56 (2H, s, exchange with D_2_O, SO_2_N*H*_2_), 7.97 (2H, d, *J* 8.2), 8.17 (2H, d, *J* 8.2), 9.85 (1H, s, exchange with D_2_O, N*H*); δ_C_ (100 MHz, DMSO-*d*_6_) 117.0, 117.1, 123.7, 126.5, 127.7, 127.7, 129.4, 138.6, 144.2, 147.3, 165.3; *m*/*z* (ESI negative) 289.9 [M-H]^−^. Elemental analysis calculated (%): C 53.60, H 4.50, N 14.42; found: C 53.65, H 4.53, N 14.39.

#### 3.1.4 *N*-(2-Aminophenyl)-4-chloro-3-sulfamoylbenzamide (5)

Obtained according to the above procedure using *o*-aminoaniline (**1**) and 4-chloro-3-sulfamoylbenzoic acid (**3**). 42% yield; δ_H_ (400 MHz, DMSO-*d*_6_) 5.00 (2H, s, exchange with D_2_O), 6.63 (1H, t, *J* 7.2), 6.81 (1H, d, *J* 7.2), 7.03 (1H, t, *J* 7.2), 7.17 (1H, d, *J* 7.2), 7.78 (2H, s, exchange with D_2_O, *SO*_2_ *NH*_2_), 7.85 (1H, d, *J* 8.2), 8.25 (1H, dd, *J* 1.9, 8.2), 8.58 (1H, d, *J* 2), 9.97 (1H, s, exchange with D_2_O, N*H*); δ_C_ (100 MHz, DMSO-*d*_6_) 117.0, 117.1, 123.6, 127.8, 127.9, 129.6, 132.5, 133.0, 134.2, 134.6, 142.0, 144.3, 164.4; *m*/*z* (ESI positive) 325.9 [M + H]^+^. Elemental analysis calculated (%): C 47.93, H 3.71, N 12.90; found: C 4.95, H 3.73, N 12.86.

#### 3.1.5 Synthesis of *N*-(2-Aminophenyl)-4-(4-sulfamoylbenzamido) Benzamide (8)

Obtained according to the above procedure using *o*-aminoaniline (**1**) and 4-(4-sulfamoylbenzamido) benzoic acid (**7**). 93% yield; δ_H_ (400 MHz, DMSO-*d*_6_) 4.95 (2H, brs, exchange with D_2_O, NH_2_), 6.64 (1H, t, *J* 7.8), 6.82 (1H, d, *J* 7.8), 7.01 (1H, t, *J* 7.8), 7.21 (1H, d, *J* 7.8), 7.58 (2H, s, exchange with D_2_O, SO_2_NH_2_), 7.95 (2H, d, *J* 8.6), 7.99–8.07 (4H, m), 8.17 (2H, d, *J* 8.4), 9.65 (1H, s, exchange with D_2_O, NH), 10.70 (1H, s, exchange with D_2_O, NH); δ_C_ (100 MHz, DMSO-*d*_6_) 117.1, 117.2, 120.4, 124.4, 126.7, 127.4, 127.6, 129.4, 129.6, 130.7, 138.5, 142.6, 144.1, 147.7, 165.7, 165.8; *m*/*z* (ESI negative) 409.0 [M-H]^−^. Elemental analysis calculated (%): C 58.53, H 4.42, N 13.65; found: C 58.56, H 4.45, N 16.67.

#### 3.1.6 Synthesis of *N*-(2-Aminophenyl)-4-((3-(4-sulfamoylphenyl) Ureido) Methyl)­benzamide (11)

Obtained according to the above procedure using *o*-aminoaniline (**1**) and 4-((3-(4-sulfamoylphenyl) ureido)­methyl) benzoic acid (**9**). 67% yield; δ_H_ (400 MHz, DMSO-*d*_6_) 4.43 (2H, d, *J* 5.8), 4.96 (2H, brs, exchange with D_2_O, N*H*_2_), (1H, dt, *J* 1.2, 7.6), 6.82 (1H, dd, *J* 1.3, 6.7), 6.90 (1H, t, *J* 6), 7.00 (1H, dt, *J* 1.4, 7.8), 7.18–7.23 (3H, m, exchange with D_2_O, SO_2_N*H*_2_, N*H*), 7.46 (2H, d, *J* 8.2), 7.60 (2H, d, *J* 8.9), 7.71 (2H, d, *J* 8.9), 7.98 (2H, d, *J* 8.2), 9.07 (1H, s, exchange with D_2_O, N*H*), 9.65 (1H, s, exchange with D_2_O, N*H*); δ_C_ (100 MHz, DMSO-*d*_6_) 44.5, 114.3, 118.3, 118.9, 122.5, 125.5, 127.3, 128.4, 129.4, 132.2, 136.7, 141.9, 142.1, 149.6, 154.8, 170.3; *m*/*z* (ESI positive) 440.1 [M + H]^+^. Elemental analysis calculated (%): C 57.39, H 4.82, N 15.94; found: C 57.42, H 4.79, N 15.92.

#### 3.1.7 Synthesis of *N*-(2-Aminophenyl)-4-((3-(3-sulfamoylphenyl) Ureido) Methyl)­benzamide (12)

Obtained according to the above procedure using *o*-aminoaniline (**1**) and 4-((3-(3-sulfamoylphenyl) ureido) methyl) benzoic acid (**10**). 12% yield; δ_H_ (400 MHz, DMSO-*d*_6_) 4.42 (2H, d, *J* 5.9), 4.92 (2H, brs, exchange with D_2_O, N*H*_2_), 6.63 (1H, td, *J* 1.5, 7.6), 6.81 (1H, dd, *J* 7.6), 6.93 (1H, brs, exchange with D_2_O, N*H*), 7.00 (1H, td, *J* 1.5, 7.6), 7.20 (1H, d, *J* 8.2), 7.33 (2H, s, SO_2_NH_2_), 7.88 (1H, dt, J 1.5, 7.6), 7.42–7.48 (3H, m), 7.57 (1H, m), 7.98 (2H, d, *J* 8.2), 8.07 (1H, t, *J* 1.8), 9.12 (1H, brs, exchange with D_2_O, N*H*), 9.66 (1H, s, exchange with D_2_O, N*H*); δ_C_ (100 MHz, DMSO-*d*_6_) 43.5, 115.6, 117.1, 117.2, 119.1, 121.5, 124.3, 127.4, 127.6, 127.8, 128.8, 130.2, 134.1, 141.8, 144.0, 144.8, 145.5, 156.0, 166.1; *m*/*z* (ESI negative) 438.1 [M-H]^−^. Elemental analysis calculated (%): C 57.39, H 4.82, N 15.94; found: C 57.41, H 4.79, N 16.01.

#### 3.1.8 Synthesis of 5-Fluoro-2-(4-((3-(3-sulfamoylphenyl) Ureido) Methyl) Benzamido) Benzenaminium (14)

*tert*-Butyl (5-fluoro-2-(4-((3-(3-sulfamoylphenyl) ureido) methyl) benzamido) phenyl) carbamate (**13**) (100 mg, 0.21 mmol) was treated with TFA-DCM (3.5 mL, 4 mL). The reaction mixture was stirred for 3 h at room temperature and then evaporated to dryness; the addition of Et_2_O (10 mL) under vigorous stirring yielded white solids that were filtered, to afford the tittle compound 14 as a white powder (75 mg, 74%). δ_H_ (400 MHz, DMSO-*d*_6_) 4.42 (2H, d, *J* 5.2), 6.42 (1H, t, *J* 8), 6.59 (1H, m), 6.85 (1H, t, *J* 5.6, exchange with D_2_O, N*H*), 7.15 (1H, m), 7.34–7.47 (5H, m, 2H exchange with D_2_O, SO_2_NH_2_), 7.57 (1H, d, 7.8), 7.98 (1H, d, *J* 7.8), 8.06 (1H, s, exchange with D_2_O, N*H*), 9.05 (1H, s, exchange with D_2_O, NH), 9.62 (1H, m, 2H exchange with D_2_O, N*H*); δ_C_ (100 MHz, DMSO-*d*_6_) 43.5, 103.0 (d, *J*_C–F_ 25), 103.7 (d, *J*_C–F_ 22), 115.6, 119.2, 120.7, 121.5, 127.8, 128.8, 129.5 (d, *J*_C–F_ 10.5), 130.2, 133.9, 141.8, 144.9, 145.4, 145.5, 156.0, 161.9 (d, *J*_C–F_ 239), 166.4; δ_f_ (376 MHz, DMSO-*d*_6_) −74.5, −116.5; *m*/*z* (ESI positive) 458.0 [M + H]^+^. Elemental analysis calculated (%): C 48.34, H 3.70, N 12.25; found: C 48.37, H 3.69, N 12.27.

#### 3.1.9 Synthesis of *N*-(2-Aminophenyl)-4-(3-(4-sulfamoylphenethyl) Ureido) Benzamide (18)

Obtained according to the above procedure using *o*-aminoaniline (**1**) and 4-(3-(4-sulfamoylphenethyl) ureido) benzoic acid (**17**). 64% yield; δ_H_ (400 MHz, DMSO-*d*_6_) 2.89 (2H, t, *J* 7), 3.43 (2H, q, *J* 7), 4.90 (2H, s, exchange with D_2_O, NH_2_), 6.41 (1H, t, *J* 7), 6.63 (1H, t, *J* 7.6), 6.81 (1H, d, *J* 7.6), 7.00 (1H, t, *J* 7.6), 7.19 (1H, d, *J* 7.6), 7.35 (2H, s, exchange with D_2_O, SO_2_NH_2_), 7.48 (2H, d, *J* 8.3), 7.54 (2H, d, *J* 8.8), 7.80 (2H, d, *J* 8.3), 7.91 (2H, d, *J* 8.8), 8.94 (1H, s, exchange with D2O, N*H*), 9.53 (1H, s, exchange with D_2_O, N*H*); δ_C_ (100 MHz, DMSO-*d*_6_) 36.4, 41.2, 117.1, 117.2, 117.4, 124.6, 126.7, 127.2, 127.5, 127.6, 129.7, 130.1, 143.0, 144.0, 144.5, 144.7, 155.8, 165.8; *m*/*z* (ESI negative) 452.1 [M-H]^−^. Elemental analysis calculated (%): C 58.27, H 5.11, N 15.44; found: C 58.30, H 5.14, N 15.47.

#### 3.1.10 Synthesis of *N*-(2-Aminophenyl)-4-(3-(4-sulfamoylphenyl) Thioureido) Benzamide (21)

Obtained according to the above procedure using *o*-aminoaniline (**1**) and 4-(3-(4-sulfamoylphenyl) ureido) benzoic acid (**19**). 75% yield; δ_H_ (400 MHz, DMSO-*d*_6_) 4.93 (2H, s, exchange with D_2_O, N*H*_2_), 6.64 (1H, t, *J* 7.6), 6.83 (1H, d, *J* 7.6), 7.01 (1H, t, *J* 7.8), 7.21 (1H, d, *J* 7.6), 7.34 (1H, s, exchange with D_2_O, SO_2_N*H*_2_), 7.69–7.75 (4H, m), 7.82 (2H, d, *J* 8.8), 8.00 (2H, d, *J* 8.6), 9.64 (1H, s, exchange with D_2_O, N*H*); δ_C_ (100 MHz, DMSO-*d*_6_) 116.4, 116.8, 122.1, 122.9, 123.5, 125.0, 127.6, 127.9, 128.2, 129.4, 130.1, 131.4, 141.3, 143.6, 144.2, 145.4, 168.7, 181.1; *m*/*z* (ESI negative) 440.0 [M-H]^−^. Elemental analysis calculated (%): C 54.41, H 4.34, N 15.86; found: C 54.43, H 4.36, N 15.88.

#### 3.1.11 Synthesis of *N*-(2-Aminophenyl)-4-(3-(3-sulfamoylphenyl) Thioureido) Benzamide (22)

Obtained according to the above procedure using *o*-aminoaniline (**1**) and 4-(3-(3-sulfamoylphenyl) ureido) benzoic acid (**20**). 78% yield; δ_H_ (400 MHz, DMSO-*d*_6_) 4.93 (2H, s, exchange with D_2_O, N*H*_2_), 6.64 (1H, t, *J* 7.8), 6.82 (1H, d, *J* 7.8), 7.01 (1H, t, *J* 7.8), 7.21 (1H, d, *J* 7.8), 7.43 (2H, s, exchange with D_2_O, SO_2_N*H*_2_), 7.57 (1H, t, *J* 7.8), 7.62 (1H, d, *J* 7.8), 7.70 (2H, d, *J* 8.6), 7.77 (1H, d, *J* 7.8), 8.00 (2H, d, *J* 7.6), 8.04 (1H, s), 9.64 (1H, s, exchange with D_2_O, N*H*), 10.22 (2H, m, exchange with D_2_O, N*H*); δ_C_ (100 MHz, DMSO-*d*_6_) 117.0, 117.2, 121.4, 122.4, 123.1, 124.4, 127.3, 127.5, 127.6, 129.1, 129.9, 131.0, 140.8, 143.1, 143.9, 145.2, 165.6, 180.6; *m*/*z* (ESI negative) 440.1 [M-H]^−^. Elemental analysis calculated (%): C 54.41, H 4.34; N, 15.86; found: C 54.43, H 4.36, N 15.84.

#### 3.1.12 Synthesis of *N*-(2-Aminophenyl)-4-(3-(4-sulfamoylbenzyl) Thioureido)­benzamide (29)

Obtained according to the above procedure using *o*-aminoaniline (**1**) and 4-(3-(4-sulfamoylbenzyl) thioureido)­benzoic acid (**27**). 6% yield; δ_H_ (400 MHz, DMSO-*d*_6_) 4.88 (4H, m, 2H exchange with D_2_O, N*H*_2_), 7.64 (1H, t, *J* 7.6), 6.82 (1H, d, *J* 7.6), 7.00 (1H, t, *J* 7.6), 7.20 (1H, d, *J* 7.6), 7.35 (2H, s, exchange with D_2_O, SO_2_N*H*_2_), 7.54 (2H, d, *J* 8.2), 7.66 (2H, d, *J* 8.5), 7.83 (2H, d, *J* 8.2), 7.99 (2H, m), 8.50 (1H, m), 9.61 (1H, m), 9.99 (1H, m); δ_C_ (100 MHz, DMSO-*d*_6_) 47.6, 117.1, 117.2, 122.7, 124.4, 126.6, 127.4, 127.6, 128.6, 129.3, 130.6, 143.2, 143.6, 144.0, 144.0, 165.8, 181.9; *m*/*z* (ESI negative) 454.1 [M-H]^−^. Elemental analysis calculated (%): C 55.37, H 4.65, N 15.37; found: C 55.39, H 4.62, N 15.40.

#### 3.1.13 Synthesis of *N*-(2-Aminophenyl)-4-(3-(4-sulfamoylphenethyl) Thioureido) Benzamide (30)

Obtained according to the above procedure using *o*-aminoaniline (**1**) and 4-(3-(4-sulfamoylphenethyl) thioureido) benzoic acid (**28**). 35% yield; δ_H_ (400 MHz, DMSO-*d*_6_) 3.02 (2H, t, *J* 6.4), 3.80 (2H, m), 4.90 (2H, s, exchange with D_2_O, N*H*_2_), 6.63 (1H, t, *J* 7.4), 6.82 (1H, d, *J* 7.4), 7.00 (1H, t, *J* 7.4), 7.20 (1H, d, *J* 7.4), 7.34 (2H, s, exchange with D_2_O, SO_2_N*H*_2_), 7.50 (2H, d, *J* 8), 7.60 (2H, d, *J* 8.2), 7.82 (2H, d, *J* 8), 7.97 (3H, m, 1H exchange with D_2_O, N*H*), 9.60 (1H, s, exchange with D_2_O, N*H*); δ_C_ (100 MHz, DMSO-*d*_6_) δ_C_ (100 MHz, DMSO-*d*_6_) 35.7, 47.9, 117.5, 117.6, 122.9, 124.8, 127.0, 127.7, 127.9, 128.9, 129.5, 130.8, 143.5, 143.8, 144.5, 166.1, 181.7; *m*/*z* (ESI negative) 468.1 [M-H]^−^. Elemental analysis calculated (%): C 56.27, H 4.94, N 14.91; found: C 55.23, H 4.92, N 15.87.

#### 3.1.14 Synthesis of *N*-(2-Aminophenyl)-4-((2-oxo-2-((4-sulfamoylbenzyl) Amino) Ethyl) Amino)­benzamide (37)

Obtained according to the above procedure using *o*-aminoaniline (**1**) and 4-((2-oxo-2-((4-sulfamoylbenzyl) amino) ethyl) amino) benzoic acid (**34**). 43% yield; δ_H_ (400 MHz, DMSO-*d*_6_) 3.85 (2H, d, *J* 5.88), 4.41 (2H, d, *J* 5.92), 4.86 (2H, s, N*H*_2_), 6.61–6.67 (4H, m), 6.81 (1H, d, *J* 7.9), 6.98 (1H, t, *J* 7.9), 7.18 (1H, d, *J* 7.9), 7.34 (2H, s, SO_2_N*H*_2_), 7.44 (2H, d, *J* 8.2), 7.80 (2H, d, *J* 8.2), 7.83 (2H, d, *J* 8.7), 8.62 (1H, t, *J* 5.92, N*H*), 9.36 (1H, s, N*H*); δ_C_ (100 MHz, DMSO-*d*_6_) 42.2, 54.7, 113.4, 115.6, 119.0, 123.2, 123.8, 126.2, 127.4, 128.1, 129.3, 131.3, 140.6, 141.4, 149.1, 151.7, 171.2, 173.1; *m*/*z* (ESI negative) 452.1 [M-H]^−^. Elemental analysis calculated (%): C 58.27, H 5.11, N 15.44; found: C 58.24, H 5.13, N 15.48.

#### 3.1.15 Synthesis of *N*-(2-Aminophenyl)-4-((2-oxo-2-((4- sulfamoylphenethyl) Amino) Ethyl) Amino) Benzamide (38)

Obtained according to the above procedure using *o*-aminoaniline (**1**) and 4-((2-oxo-2-((4-sulfamoylphenethyl) amino) ethyl) amino) benzoic acid (**35**). 36% yield; purified by 10% MeOH in DCM; δ_H_ (400 MHz, DMSO-*d*_6_) 2.83 (2H, t, *J* 7.1), 3.39 (2H, m), 3.72 (2H, d, *J* 6), 4.85 (2H, brs, exchange with D_2_O, N*H*_2_), 6.55–6.65 (4H, m, 1H exchange with D_2_O, N*H*), 6.81 (1H, dd, *J* 1.4, 7.6), 6.98 (1H, td, *J* 1.4, 7.6), 7.18 (1H, dd, *J* 1.4, 7.6), 7.33 (2H, s, exchange with D_2_O, SO_2_N*H*_2_), 7.40 (2H, d, *J* 8.4), 7.76 (2H, d, *J* 8.4), 7.81 (2H, d, *J* 8.8), 8.09 (1H, t, *J* 6, exchange with D_2_O, N*H*), 9.35 (1H, s, exchange with D_2_O, N*H*); δ_C_ (100 MHz, DMSO-*d*_6_) 35.8, 40.7, 47.2, 112.1, 117.1, 117.3, 122.7, 125.0, 126.6, 126.9, 127.4, 130.0, 130.1, 143.0, 143.9, 144.5, 152.0, 166.0, 170.6; *m*/*z* (ESI negative) 466.2 [M-H]^−^. Elemental analysis calculated (%): C 59.09, H 5.39, N 14.98; found: C 59.11, H 5.39, N 15.04.

#### 3.1.16 Synthesis of *N*-(2-Aminophenyl)-4-((2-oxo-2-((5-(4-sulfamoylphenoxy) Pentyl) Amino) Ethyl) Amino) Benzamide (39)

Obtained according to the above procedure using *o*-aminoaniline (**1**) and 4-((2-oxo-2-((5-(4-sulfamoylphenoxy) pentyl) amino) ethyl) amino) benzoic acid (**36**). 35% yield; δ_H_ (400 MHz, DMSO-*d*_6_) 1.38–1.54 (4H, m), 1.75 (2H, pent, *J* 7.1), 3.15 (2H, q, *J* 6.1), 3.74 (2H, d, *J* 5.8), 4.05 (2H, t, *J* 6.4), 4.85 (2H, s, brs, exchange with D_2_O, N*H*_2_), 6.55 (1H, t, exchange with D_2_O, N*H*), 6.60–6.64 (3H, m), 6.80 (1H, d, *J* 8), 6.98 (1H, d, *J* 7.6), 7.09 (2H, d, *J* 8.9), 7.17 (1H, d, *J* 8), 7.21 (2H, s, exchange with D_2_O, SO_2_N*H*_2_), 7.76 (2H, d, *J* 8.9), 7.82 (2H, d, *J* 8.7), 7.98 (1H, t, *J* 5.8, exchange with D_2_O, N*H*), 9.34 (1H, s, exchange with D_2_O, N*H*); δ_C_ (100 MHz, DMSO-*d*_6_) δ_C_ (100 MHz, DMSO-*d*_6_) 23.3, 29.1, 29.5, 38.4, 56.2, 78.1, 111.6, 114.3, 115.4, 119.7, 123.5, 126.4, 127.8, 130.1, 135.6, 150.0, 152.3, 163.5, 172.2, 174.6; *m*/*z* (ESI negative) 524.2 [M-H]^−^. Elemental analysis calculated (%): C 59.41, H 5.94, N 13.32; found: C 59.48, H 5.87, N 13.25.

### 3.2 *In Vitro* Carbonic Anhydrase Inhibition Assay

An Applied Photophysics stopped-flow instrument was used to assay the CA-catalyzed CO_2_ hydration activity.^55^ Phenol red (at a concentration of 0.2 mM) was used as an indicator, working at the absorbance of 557 nm, with 20 mM Hepes (pH 7.4) as a buffer, and 20 mM Na_2_SO_4_ (to maintain constant ionic strength), following the initial rates of the CA-catalyzed CO_2_ hydration reaction for a period of 10–100 s. The CO_2_ concentrations ranged from 1.7 to 17 mM for the determination of the kinetic parameters and inhibition constants. Enzyme concentrations ranged between 5 and 12 nM. For each inhibitor, at least six traces of the initial 5–10% of the reaction were used to determine the initial velocity. The uncatalyzed rates were determined in the same manner and subtracted from the total observed rates. Stock solutions of the inhibitor (0.1 mM) were prepared in distilled–deionized water and dilutions up to 0.01 nM were done thereafter with the assay buffer. Inhibitor and enzyme solutions were preincubated together for 15 min at room temperature prior to the assay to allow for the formation of the E–I complex. The inhibition constants were obtained by nonlinear least-squares methods using PRISM 3 and the Cheng-Prusoff equation, as reported earlier^68^ and represent the mean from at least three different determinations. All hCA isoforms were recombinant proteins obtained in-house, as reported earlier.^69^

### 3.3 *In Vitro* HDAC Inhibition Assay

Biochemical evaluation of the compounds against human recombinant HDACs 1, 3, 4, 6, and 8 was performed in a 10-dose mode with 3-fold serial dilution starting from a 100 μM solution. Inhibition values for each compound tested toward the HDAC isozyme were measured using the homogeneous fluorescence release HDAC assay. Purified recombinant enzymes were incubated with serially diluted inhibitors at the indicated concentration. The deacetylase activities of HDACs 1, 3, 4, 6, and 8 in the presence of serial dilution of each compound were determined by assaying enzyme activity using the p53 residues 379–382 (Arg-His-Lys-Lys­(Ac)­AMC) to detect inhibitory activity against HDACs 1, 3, and 6, the diacetylated peptide from p53 residues 379–382 (Arg-His-Lys­(Ac)-Lys­(Ac)­AMC) to detect inhibition against HDAC8, and the fluorogenic class IIa (Boc-Lys­(trifluoroacetyl)-AMC) substrate to detect inhibition against HDAC4.^56^ Deacetylated/detrifluoroacetylated AMC substrates were sensitive to lysine peptidase, and free fluorogenic 4-methylcoumarin-7-amide was generated, which can be excited at 355 nm and observed at 460 nm (Reaction Biology Corporation, MD, USA). Data were analyzed on a plate-to-plate basis in relation to the control and imported into analytical software (GraphPad Prism, CA, USA).

### 3.4 X-ray Crystallography

#### 3.4.1 Crystallization and X-ray Data Collection

Crystals of hCA II were obtained using the hanging drop vapor diffusion method using a 24-well Linbro plate. Two μL of a 10 mg/mL solution of hCA II in Tris-HCl 20 mM, pH 8.0, were mixed with 2 μL of a solution containing 1.5 M sodium citrate and 0.1 M Tris, pH 8.0, and equilibrated against the same solution at 296 K. The complexes were prepared by soaking the native crystals in the mother liquor solution containing the inhibitors at a concentration of 10 mM for 2 days. All crystals were flash-frozen at 100 K using a solution obtained by adding 15% (v/v) glycerol to the mother liquor solution as a cryoprotectant. Data on hCA II/**39** and hCA II/**40** crystals of the complexes were collected by using synchrotron radiation at the XRD2 beamline at Elettra Synchrotron (Trieste, Italy) with a wavelength of 1.000 Å and a DECTRIS Pilatus 6M detector. Data were integrated and scaled using the program XDS.^70^ Data processing statistics are shown in Table S1 in the Supporting Information file.

#### 3.4.2 Structural Determination

The crystal structure of hCA II (PDB accession code: 4FIK) without solvent molecules and other heteroatoms was used to obtain initial phases using Refmac5.^71^ 5% of the unique reflections were selected randomly and excluded from the refinement data set for the purpose of Rfree calculations. The initial |Fo – Fc| difference electron density maps unambiguously showed the inhibitor molecules. The inhibitor was introduced in the model with a 1.0 occupancy. Refinements proceeded using normal protocols of positional and isotropic atomic displacement parameters, alternating with manual building of the models using COOT.^72^ The quality of the final models was assessed with COOT and RAMPAGE.^73^ Crystal parameters and refinement data are summarized in the Supporting Information file. Atomic coordinates were deposited in the Protein Data Bank (PDB accession codes: 7QSE and 7QRK). Graphical representations were generated with UCSF Chimera.^74^

### 3.5 Molecular Modeling

All molecular modeling calculations were performed on the www.3d-qsar.com portal.^75^ The HDAC complexes of SAHA and TSA were directly imported through the Py-PDB module, and all undesired molecules were removed. The Zn ions were kept, and hydrogen was added via the embedded Reduce Python module. The cleaned complexes were then solvated with a layer of explicit water (5 Å) molecules and subjected to a single-point minimization of 2000 iterations using the UFF force field^76^ as implemented in Open Babel.^77^ The minimized complexes were separated into protein (lock) and ligand (key) pairs. Through the Py-Docking module, a docking assessment procedure was then run to select the docking program best able to reproduce the experimental binding conformation. A list of 27 docking program/scoring function pairs was used in the redocking experiments, using either the experimental binding conformation (ECRD) or a randomized one (RCRD). The best-performing docking program was applied to the title derivatives to investigate the putative binding conformation in either HDAC1 or HDAC3. The docked conformations were visually inspected with UCSF Chimera^74^ and the PoseView web application.^65^

### 3.6 Biology

#### 3.6.1 Cell Lines

Primary A375,^78^ Sk-Mel-28, and metastatic 501Mel^79^ melanoma cells; primary HCT8 (American Type Culture Collection, ATCC) and HCT116 ^80^ colon carcinoma cells; and triple-negative MDAMB231 (ATCC) and invasive ductal BT-474 (ATCC) breast cancer cells were grown in DMEM with 4.5 g/L glucose, supplemented with 2 mM/L glutamine and 10% FBS. Endothelial colony-forming cells (ECFC)^81^ were used as normal cell lines. ECFCs were grown in an EGM-2 EC growth medium-2 Bullet Kit (Lonza, Basel, Switzerland) on Attachment Factor Solution (Sigma-Aldrich, Milan, Italy)-coated dishes. All cell lines were maintained at 37 °C under a humidified atmosphere.

#### 3.6.2 MTT Assay

Cell viability was assessed using the MTT (3-(4,5-dimethylthiazol-2-yl)-2,5-diphenyltetrazolium bromide) tetrazolium reduction assay (Sigma-Aldrich). Cells were plated in 96-well plates in complete medium, and compounds **11** and **14** were added to the culture medium for 72 h. The culture medium was then replaced with MTT reagent according to the manufacturer’s instructions, and plates were incubated at 37 °C for 2 h. MTT was removed, and the blue MTT–formazan product was solubilized with Dimethyl sulfoxide (DMSO) (Sigma-Aldrich). The absorbance of the formazan solution was read at 595 nm using the microplate reader (Bio-Rad, Milan, Italy). IC_50_ values were interpolated by GraphPad Prism 10.1 software.

#### 3.6.3 Flow Cytometry

To evaluate the expression level of CA IX on the cell membrane, tumor cells were seeded at 70% confluency. The day after, cells were gently detached from the tissue culture dishes with a scraper, collected in flow cytometer tubes, washed in PBS, and centrifuged at 300 g for 5 min. Cells were stained with 200 μL of 1:1000 anti-CA IX (66243-1, Proteitech) PBS solution for 1 h at room temperature and for 30 min in the dark at room temperature with a 1:1000 goat antimouse IgG AF-647 (A21235, Invitrogen) PBS solution. Cells were washed in PBS and analyzed with BD FACSCanto II (BD Biosciences) using irrelevant IgG to set the gating. To quantify cell death, the CellEvent Caspase-3/7 Detection kit (C10423, Invitrogen) was used as previously described.^82^

#### 3.6.4 Apoptosis Assay

Apoptosis was evaluated using the Annexin V-FITC/Propidium Iodide (PI) staining method with an Annexin V-FITC apoptosis detection kit (Enzo Life Science). Briefly, after treatment, 2 × 10^5^ cells were harvested, washed with PBS, and resuspended in 100 μL of 1× binding buffer and incubated with 5 μL of Annexin V-FITC at room temperature for 15 min in the dark. After incubation, 600 μL of 1× binding buffer containing 5 μL of PI was added to each sample, and the cells were analyzed by flow cytometry using a FACSCalibur flow cytometer (BD Biosciences). For each sample, at least 10 000 events were collected. The percentages of viable cells (Annexin V^–^/PI^–^), early apoptotic cells (Annexin V^+^/PI^–^), late apoptotic cells (Annexin V^+^/PI^+^), and necrotic cells (Annexin V^–^/PI^+^) were determined using Kaluza software (Beckman Coulter).

#### 3.6.5 Western Blot

Cells were lysed, and total extracts were fractionated by SDS-PAGE, transferred to a nitrocellulose filter, and subjected to an immunoblot assay, as previously described.^83^ Between 15 and 30 μg of total proteins were resolved under reducing conditions by SDS-PAGE in 15% gels, transferred to a nitrocellulose filter (Bio-Rad Laboratories, 1,620,112), blocked with 5% albumin bovine serum (BSA) (Sigma-Aldrich, A4503) or 5% low-fat dry milk, and subjected to immunoblot assay.

The following antibodies were used: Acetyl-Histone H3 (Lys9) Antibody #9671 (Cell Signaling), anti-GAPDH (Santa Cruz Biotechnology, sc-32233). The following secondary antibodies were used: antimouse (Bio-Rad Laboratories, 1,706,515) or antirabbit (Bio-Rad Laboratories, 1,706,516) IgG- horseradish peroxidase-conjugated antibodies. Signals were detected by enhanced chemiluminescence (Cyanagen, Westar Sun XLS142 and Westar Antares XLS063). Densitometric evaluation of band intensity was performed using Image Lab or ImageJ software, and values were normalized to loading controls.

#### 3.6.6 Statistical Analysis

All experiments were performed in triplicate (*N* = 3), and data are presented as mean ± standard deviation (SD). Statistical significance was determined using one/two-way analysis of variance (ANOVA), followed by Dunnett’s multiple comparison test to compare multiple conditions to the control group. Differences were considered statistically significant at *p < 0.05, **p < 0.01, ***p < 0.001, ****p < 0.0001. GraphPad Prism 10.4.1 software (GraphPad Software, San Diego, CA, USA) was used for all statistical analyses and graph generation.

## Supplementary Material





## Notes

The Supporting Information file is available free of charge on the The Supporting Information is available free of charge at https://pubs.acs.org/doi/10.1021/acs.jmedchem.5c01788.

SMILES representation for compounds (CSV)
Synthetic procedures for compounds **B, 6, 7, E, F, 9, 10, 13, G, Q, R, H, 16, 17, I, J, 19, 20, K, 25, 26, 27, 28, L, M, 31, 32, 33, 34, 35, 36, 11a, 11b, 42, 43, 40**; ^1^H, ^13^C, and ^19^F NMR spectra of compounds **4, 5, 8, 11, 11a, 11b, 12, 14, 18, 21, 22, 29, 30, 37, 38, 39, 40**; Summary of data collection and atomic model refinement statistics for hCA II/38 and hCA II/37; Electron density of inhibitors **38** (**A**) and **37** (**B**); SAHA- and TSA-bound conformations in different HDACs; List of programs used; Inhibition data of hCAs and HDACs with compound **40**; Flow cytometry of hCA IX basal expression in MDA-MB-231, BT-474, A375, 501Mel, and Sk-Mel-28; IC_50_ values of **11** on HCT-8, HCT-116, MDA-MB-231, BT-474, A375, 501Mel, and Sk-Mel-28; IC_50_ values of **14** on HCT-8, HCT-116, MDA-MB-231, BT-474, A375, 501Mel, and Sk-Mel-28; IC_50_s of SAHA in HCT-8, HCT-116, MDA-MB-231, BT-474, A375, 501Mel, Sk-Mel-28, and normal endothelial colony-forming cells (ECFC) (PDF)

## Abbreviations

CA: carbonic anhydrase
CAI: carbonic anhydrase inhibitor
HDAC: histone deacetylase
HDACI: histone deacetylase inhibitor

## References

[1] Wang G. L., Jiang B. H., Rue E. A., Semenza G. L. (1995). Hypoxia-inducible
factor 1 is a basic-helix-loop-helix-PAS heterodimer regulated by
cellular O2 tension. Proc. Natl. Acad. Sci.
U. S. A..

[2] Gatenby R. A., Gillies R. J. (2004). Why do cancers have high aerobic glycolysis?. Nat. Rev. Cancer.

[3] Vaupel P., Mayer A. (2014). Hypoxia in tumors: Pathogenesis-related classification, characterization
of hypoxia subtypes, and associated biological and clinical implications. Adv. Exp. Med. Biol..

[4] Warburg O., Wind F., Negelein E. (1927). The Metabolism of Tumors
in the Body. J. Gen. Physiol..

[5] Wykoff C. C., Beasley N. J., Watson P. H., Turner K. J., Pastorek J., Sibtain A., Wilson G. D., Turley H., Talks K. L., Maxwell P. H. (2000). Hypoxia-inducible
expression of tumor-associated
carbonic anhydrases. Cancer Res..

[6] Keith B., Johnson R. S., Simon M. C. (2012). HIF1alpha
and HIF2alpha: Sibling
rivalry in hypoxic tumour growth and progression. Nat. Rev. Cancer.

[7] Ruzzolini J., Laurenzana A., Andreucci E., Peppicelli S., Bianchini F., Carta F., Supuran C. T., Romanelli M. N., Nediani C., Calorini L. (2020). A potentiated cooperation of carbonic
anhydrase IX and histone deacetylase inhibitors against cancer. J. Enzyme Inhib. Med. Chem..

[8] Ledaki I., McIntyre A., Wigfield S., Buffa F., McGowan S., Baban D., Li J. L., Harris A. L. (2015). Carbonic anhydrase
IX induction defines a heterogeneous cancer cell response to hypoxia
and mediates stem cell-like properties and sensitivity to HDAC inhibition. Oncotarget.

[9] Chen Z., Ai L., Mboge M. Y., Tu C., McKenna R., Brown K. D., Heldermon C. D., Frost S. C. (2018). Differential expression and function
of CAIX and CAXII in breast cancer: A comparison between tumorgraft
models and cells. PLoS One.

[10] Krasavin M., Shetnev A., Baykov S., Kalinin S., Nocentini A., Sharoyko V., Poli G., Tuccinardi T., Korsakov M., Tennikova T. B. (2019). Pyridazinone-substituted
benzenesulfonamides display potent inhibition of membrane-bound human
carbonic anhydrase IX and promising antiproliferative activity against
cancer cell lines. Eur. J. Med. Chem..

[11] Pastorekova S., Gillies R. J. (2019). The role of carbonic
anhydrase IX in cancer development:
Links to hypoxia, acidosis, and beyond. Cancer
Metastasis Rev..

[12] Liao S. -Y., Lerman M. I., Stanbridge E. J. (2009). Expression
of transmembrane carbonic
anhydrases, CAIX and CAXII, in human development. BMC Dev. Biol..

[13] Lock F. E., McDonald P. C., Lou Y., Serrano I., Chafe S. C., Ostlund C., Aparicio S., Winum J. Y., Supuran C. T., Dedhar S. (2013). Targeting carbonic
anhydrase IX depletes breast cancer
stem cells within the hypoxic niche. Oncogene.

[14] McDonald P. C., Chia S., Bedard P. L., Chu Q., Lyle M., Tang L., Singh M., Zhang Z., Supuran C. T., Renouf D. J. (2020). A Phase 1 Study of SLC-0111,
a Novel Inhibitor
of Carbonic Anhydrase IX, in Patients With Advanced Solid Tumors. Am J Clin Oncol..

[15] Winum J. Y., Carta F., Ward C., Mullen P., Harrison D., Langdon S. P., Cecchi A., Scozzafava A., Kunkler I., Supuran C. T. (2012). Ureido-substituted sulfamates show
potent carbonic anhydrase IX inhibitory and antiproliferative activities
against breast cancer cell lines. Bioorg. Med.
Chem. Lett..

[16] Gieling R. G., Babur M., Mamnani L., Burrows N., Telfer B. A., Carta F., Winum J. Y., Scozzafava A., Supuran C. T., Williams K. J. (2012). Antimetastatic effect of sulfamate
carbonic anhydrase IX inhibitors in breast carcinoma xenografts. J. Med. Chem..

[17] Hektoen H. H., Ree A. H., Redalen K. R., Flatmark K. (2016). Sulfamate inhibitor
S4 influences carbonic anhydrase IX ectodomain shedding in colorectal
carcinoma cells. J. Enzyme Inhib. Med. Chem..

[18] Cui J., Xu H., Shi J., Fang K., Liu J., Liu F., Chen Y., Liang H., Zhang Y., Piao H. (2023). Carbonic anhydrase
IX inhibitor S4 triggers release of DAMPs related to immunogenic cell
death in glioma cells via endoplasmic reticulum stress pathway. Cell Commun. Signaling.

[19] Dubois L., Peeters S., Lieuwes N. G., Geusens N., Thiry A., Wigfield S., Carta F., McIntyre A., Scozzafava A., Dogne J. M., Supuran C. T., Harris L. A., Masereel B., Lambin B. (2011). Specific inhibition
of carbonic anhydrase IX activity
enhances the in vivo therapeutic effect of tumor irradiation. Radiother Oncol..

[20] Sadri N., Zhang P. J. (2013). Hypoxia-inducible factors: Mediators
of cancer progression;
prognostic and therapeutic targets in soft tissue sarcomas. Cancers.

[21] Maxwell P. H., Wiesener M. S., Chang G. W., Clifford S. C., Vaux E. C., Cockman M. E., Wykoff C. C., Pugh C. W., Maher E. R., Ratcliffe P. J. (1999). The tumour suppressor protein VHL targets hypoxia-inducible
factors for oxygen-dependent proteolysis. Nature.

[22] Sedlakova O., Svastova E., Takacova M., Kopacek J., Pastorek J., Pastorekova S. (2014). Carbonic anhydrase
IX, a hypoxia-induced catalytic
component of the pH regulating machinery in tumors. Front. Physiol..

[23] Vaupel P., Schlenger K., Knoop C., Hockel M. (1991). Oxygenation of human
tumors: Evaluation of tissue oxygen distribution in breast cancers
by computerized O2 tension measurements. Cancer
Res.

[24] Brahimi-Horn C., Mazure N., Pouyssegur J. (2005). Signalling via the hypoxia-inducible
factor-1alpha requires multiple posttranslational modifications. Cell. Signalling.

[25] Jeong J. W., Bae M. K., Ahn M. Y., Kim S. H., Sohn T. K., Bae M. H., Yoo M. A., Song E. J., Lee K. J., Kim K. W. (2002). Regulation and destabilization of
HIF-1alpha by ARD1-mediated
acetylation. Cell.

[26] Qian D. Z., Kachhap S. K., Collis S. J., Verheul H. M., Carducci M. A., Atadja P., Pili R. (2006). Class II histone
deacetylases are
associated with VHL-independent regulation of hypoxia-inducible factor
1 alpha. Cancer Res..

[27] Marks P., Rifkind R. A., Richon V. M., Breslow R., Miller T., Kelly W. K. (2001). Histone deacetylases
and cancer: Causes and therapies. Nat. Rev.
Cancer.

[28] Gu W., Roeder R. G. (1997). Activation of p53
sequence-specific DNA binding by
acetylation of the p53 C-terminal domain. Cell.

[29] Ito A., Kawaguchi Y., Lai C. H., Kovacs J. J., Higashimoto Y., Appella E., Yao T. P. (2002). MDM2-HDAC1-mediated deacetylation
of p53 is required for its degradation. EMBO
J..

[30] Luo J., Li M., Tang Y., Laszkowska M., Roeder R. G., Gu W. (2004). Acetylation
of p53 augments its site-specific DNA binding both in vitro and in
vivo. Proc. Natl. Acad. Sci. U. S. A..

[31] Martinez-Balbas M. A., Bauer U. M., Nielsen S. J., Brehm A., Kouzarides T. (2000). Regulation
of E2F1 activity by acetylation. EMBO J..

[32] Marzio G., Wagener C., Gutierrez M. I., Cartwright P., Helin K., Giacca M. (2000). E2F family members
are differentially
regulated by reversible acetylation. J. Biol.
Chem..

[33] Cohen H. Y., Lavu S., Bitterman K. J., Hekking B., Imahiyerobo T. A., Miller C., Frye R., Ploegh H., Kessler B. M., Sinclair D. A. (2004). Acetylation of the C terminus of Ku70 by CBP and PCAF
controls Bax-mediated apoptosis. Mol. Cell.

[34] Lombardi P. M., Cole K. E., Dowling D. P., Christianson D. W. (2011). Structure,
mechanism, and inhibition of histone deacetylases and related metalloenzymes. Curr. Opin. Struct. Biol..

[35] Richon V. M., Emiliani S., Verdin E., Webb Y., Breslow R., Rifkind R. A., Marks P. A. (1998). A class
of hybrid polar inducers
of transformed cell differentiation inhibits histone deacetylases. Proc. Natl. Acad. Sci. U. S. A..

[36] Suraweera A., O’Byrne K. J., Richard D. J. (2018). Combination Therapy With Histone
Deacetylase Inhibitors (HDACi) for the Treatment of Cancer: Achieving
the Full Therapeutic Potential of HDACi. Front.
Oncol..

[37] Li W., Sun Z. (2019). Mechanism of Action for HDAC Inhibitors-Insights from Omics Approaches. Int. J. Mol. Sci.

[38] Mullard A. (2024). FDA approvals. Nat. Rev. Drug Discovery.

[39] Ueda H., Manda T., Matsumoto S., Mukumoto S., Nishigaki F., Kawamura I., Shimomura K. (1994). FR901228,
a novel antitumor bicyclic
depsipeptide produced by Chromobacterium violaceum No. 968. III. Antitumor
activities on experimental tumors in mice. J.
Antibiot..

[40] Ryan Q. C., Headlee D., Acharya M., Sparreboom A., Trepel J. B., Ye J., Figg W. D., Hwang K., Chung E. J., Murgo A. (2005). Phase
I and pharmacokinetic
study of MS-275, a histone deacetylase inhibitor, in patients with
advanced and refractory solid tumors or lymphoma. J. Clin. Oncol..

[41] Sun Y., Hong J. H., Ning Z., Pan D., Fu X., Lu X., Tan J. (2022). Therapeutic potential of tucidinostat, a subtype-selective
HDAC inhibitor, in cancer treatment. Front.
Pharmacol..

[42] Boumber Y., Younes A., Garcia-Manero G. (2011). Mocetinostat
(MGCD0103): A review
of an isotype-specific histone deacetylase inhibitor. Expert Opin. Invest. Drugs.

[43] https://clinicaltrials.gov/study/NCT02236195 (access 26 July 2025).

[44] https://clinicaltrials.gov/study/NCT02282358 (access 26 July 2025).

[45] Methot J. L., Chakravarty P. K., Chenard M., Close J., Cruz J. C., Dahlberg W. K., Fleming J., Hamblett C. L., Hamill J. E., Harrington P. (2008). Exploration of the internal cavity of histone
deacetylase (HDAC) with selective HDAC1/HDAC2 inhibitors (SHI-1: 2). Bioorg. Med. Chem. Lett..

[46] Methot J. L., Hoffman D. M., Witter D. J., Stanton M. G., Harrington P., Hamblett C., Siliphaivanh P., Wilson K., Hubbs J., Heidebrecht R. (2014). Delayed and Prolonged Histone Hyperacetylation
with a Selective HDAC1/HDAC2 Inhibitor. ACS
Med. Chem. Lett..

[47] Liu J., Yu Y., Kelly J., Sha D., Alhassan A. B., Yu W., Maletic M. M., Duffy J. L., Klein D. J., Holloway M. K. (2020). Discovery of Highly Selective and Potent HDAC3 Inhibitors Based on
a 2-Substituted Benzamide Zinc Binding Group. ACS Med. Chem. Lett..

[48] Xiao Y., Yu D. (2021). Tumor microenvironment
as a therapeutic target in cancer. Pharmacol.
Ther..

[49] Zhang R. X., Wong H. L., Xue H. Y., Eoh J. Y., Wu X. Y. (2016). Nanomedicine
of synergistic drug combinations for cancer therapy - Strategies and
perspectives. J. Controlled Release.

[50] Fan W., Yung B., Huang P., Chen X. (2017). Nanotechnology for
Multimodal Synergistic Cancer Therapy. Chem.
Rev..

[51] Tomaselli D., Lucidi A., Rotili D., Mai A. (2020). Epigenetic polypharmacology:
A new frontierfor epi-drug discovery. Med. Res.
Rev..

[52] Supuran C. T. (2020). An update
on drug interaction considerations in the therapeutic use of carbonic
anhydrase inhibitors. Expert Opin. Drug Metab.
Toxicol..

[53] Kalinin S., Malkova A., Sharonova T., Sharoyko V., Bunev A., Supuran C. T., Krasavin M. (2021). Carbonic Anhydrase IX Inhibitors
as Candidates for Combination Therapy of Solid Tumors. Int. J. Mol. Sci..

[54] Bayat
Mokhtari R., Baluch N., Ka Hon Tsui M., Kumar S., Homayouni T. S., Aitken K., Das B., Baruchel S., Yeger H. (2017). Acetazolamide potentiates the anti-tumor
potential of HDACi, MS-275, in neuroblastoma. BMC Cancer.

[55] Khalifah R. G. (1971). The carbon
dioxide hydration activity of carbonic anhydrase. I. Stop-flow kinetic
studies on the native human isoenzymes B and C. J. Biol. Chem..

[56] Lahm A., Paolini C., Pallaoro M., Nardi M. C., Jones P., Neddermann P., Sambucini S., Bottomley M. J., Lo Surdo P., Carfí A., Koch U., De Francesco R., Steinkühler C., Gallinari P. (2007). Unraveling the hidden catalytic activity
of vertebrate class IIa histone deacetylases. Proc. Natl. Acad. Sci. U. S. A..

[57] D’Ambrosio K., Di Fiore A., Alterio V., Langella E., Monti S. M., Supuran C. T., De Simone G. (2025). Multiple Binding
Modes of Inhibitors
to Human Carbonic Anhydrases: An Update on the Design of Isoform-Specific
Modulators of Activity. Chem. Rev..

[58] Lauffer B. E. L., Mintzer R., Fong R., Mukund S., Tam C., Zilberleyb I., Flicke B., Ritscher A., Fedorowicz G., Vallero R., Ortwine D. F., Gunzner J., Modrusan Z., Neumann L., Koth C. M., Lupardus P. J., Kaminker J. S., Heise C. E., Steiner P. (2013). Histone Deacetylase (HDAC) Inhibitor
Kinetic Rate Constants Correlate with Cellular Histone Acetylation
but Not Transcription and Cell Viability. J.
Biol. Chem..

[59] https://www.cgl.ucsf.edu/chimera/.

[60] O’Boyle N. M., Morley C., Hutchison G. R. (2008). Pybel:
A Python wrapper for the OpenBabel
cheminformatics toolkit. Chem. Cent. J..

[61] Ragno R. (2019). www.3d-qsar.com:
A web portal that brings 3-D QSAR to all electronic devices-the Py-CoMFA
web application as tool to build models from pre-aligned datasets. J. Comput.-Aided Mol. Des..

[62] Tietze S., Apostolakis J. (2007). GlamDock:
Development and validation of a new docking
tool on several thousand protein-ligand complexes. J. Chem. Inf. Model..

[63] Korb O., Stutzle T., Exner T. E. (2009). Empirical scoring functions for advanced
protein-ligand docking with PLANTS. J. Chem.
Inf. Model..

[64] Liu N., Xu Z. B. (2019). Using LeDock as
a docking tool for computational drug design. Iop C Ser. Earth Env..

[65] Stierand K., Rarey M. (2010). Drawing the PDB: Protein-Ligand
Complexes in Two Dimensions. ACS Med. Chem.
Lett..

[66] Eberhardt J., Santos-Martins D., Tillack A. F., Forli S. (2021). AutoDock Vina 1.2.0:
New Docking Methods, Expanded Force Field, and Python Bindings. J. Chem. Inf. Model..

[67] Yang W. M., Tsai S. C., Wen Y. D., Fejer G., Seto E. (2002). Functional
domains of histone deacetylase-3. J. Biol. Chem..

[68] Berrino E., Michelet B., Vitse K., Nocentini A., Bartolucci G., Martin-Mingot A., Gratteri P., Carta F., Supuran C. T., Thibaudeau S. (2024). Superacid-Synthesized
Fluorinated
Diamines Act as Selective hCA IV Inhibitors. J. Med. Chem..

[69] Baroni C., D’Agostino I., Renzi G., Kilbile J. T., Tamboli Y., Ferraroni M., Carradori S., Capasso C., Carta F., Supuran C. T. (2024). Lasamide, a Potent
Human Carbonic Anhydrase Inhibitor
from the Market: Inhibition Profiling and Crystallographic Studies. ACS Med. Chem. Lett..

[70] Leslie, A. G. W. ; Powell, H. R. Processing diffraction data with mosflm.Evolving methods for macromolecular crystallography. Read, R. J. ; Sussman, J. L. ; Springer: Dordrecht, 2007; Vol. 245, pp. 41–51.

[71] Murshudov G. N., Vagin A. A., Dodson E. J. (1997). Refinement of macromolecular
structures
by the maximum-likelihood method. Acta Crystallogr.,
Sect. D: biol. Crystallogr..

[72] Emsley P., Lohkamp B., Scott W., Cowtan K. (2010). Features and
development
of Coot. Acta Crystallogr., Sect. D: Biol. Crystallogr..

[73] Lovell S. C., Davis I. W., Arendall W. B., de
Bakker P. I. W., Word J. M., Prisant M. G., Richardson J. S., Richardson D. C. (2003). Structure validation by Cα geometry: ϕ,ψ
and Cβ deviation. Proteins.

[74] Pettersen E. F., Goddard T. D., Huang C. C., Couch G. S., Greenblatt D. M., Meng E. C., Ferrin T. E. (2004). UCSF Chimera—a
visualization
system for exploratory research and analysis. J. Comput. Chem..

[75] Ragno R. (2019). www.3d-qsar.com:
A web portal that brings 3-D QSAR to all electronic devices-the Py-CoMFA
web application as tool to build models from pre-aligned datasets. J. Comput.-Aided Mol. Des..

[76] Rappe A. K., Casewit C. J., Colwell K. S., Goddard W. A. I., Skiff W. M. (1992). UFF, a
full periodic table force field for molecular mechanics and molecular
dynamics simulations. J. Am. Chem. Soc..

[77] O’Boyle N. M., Banck M., James C. A., Morley C., Vandermeersch T., Hutchison G. R. (2011). Open Babel: An open chemical toolbox. J. Chem. Inform..

[78] Laurenzana A., Margheri F., Biagioni A., Chillà A., Pimpinelli N., Ruzzolini J., Peppicelli S., Andreucci E., Calorini L., Serratì S., Del Rosso M., Fibbi G. (2019). EGFR/uPAR interaction as druggable
target to overcome vemurafenib acquired resistance in melanoma cells. E Bio Med..

[79] Andreucci E., Pietrobono S., Peppicelli S., Ruzzolini J., Bianchini F., Biagioni A., Stecca B., Calorini L. (2018). SOX2 as a
novel contributor of oxidative metabolism in melanoma cells. Cell Commun. Signaling.

[80] Andreucci E., Ruzzolini J., Peppicelli S., Bianchini F., Laurenzana A., Carta F., Supuran C. T., Calorini L. (2019). The carbonic
anhydrase IX inhibitor SLC-0111 sensitises cancer cells to conventional
chemotherapy. J. Enzyme Inhib. Med. Chem..

[81] Peri S., Biagioni A., Versienti G., Andreucci E., Staderini F., Barbato G., Giovannelli L., Coratti F., Schiavone N., Cianchi F., Papucci L., Magnelli L. (2021). Enhanced Vasculogenic Capacity Induced by 5-Fluorouracil
Chemoresistance in a Gastric Cancer Cell Line. Int. J. Mol. Sci..

[82] Andreucci E., Biagioni A., Peri S., Versienti G., Cianchi F., Staderini F., Antonuzzo L., Supuran C. T., Olivo E., Pasqualini E., Messerini L., Massi D., Lulli M., Ruzzolini J., Peppicelli S., Bianchini F., Schiavone N., Calorini L., Magnelli L., Papucci L. (2023). The CAIX inhibitor
SLC-0111 exerts anti-cancer activity on gastric cancer cell lines
and resensitizes resistant cells to 5-Fluorouracil, taxane-derived,
and platinum-based drugs. Cancer Lett..

[83] Trisciuoglio D., Ragazzoni Y., Pelosi A., Desideri M., Carradori S., Gabellini C., Maresca G., Nescatelli R., Secci D., Bolasco A. (2012). CPTH6, a thiazole derivative,
induces histone hypoacetylation and apoptosis in human leukemia cells. Clin. Cancer Res..

