# Supplementary material for: Discovery of First-in-Class Carbonic Anhydrase/Histone Deacetylase Dual Inhibitors with Antiproliferative Activity in Cancer Cells
Source: J Med Chem. 2025 Oct 28;68(21):22874–95. doi: 10.1021/acs.jmedchem.5c01788 (PMC12621190; doi:10.1021/acs.jmedchem.5c01788)
Supplement: Supplementary file 2 [file jm5c01788_si_002.pdf]

Supporting Information

Discovery of First-in-Class Carbonic Anhydrase/Histone Deacetylase Dual Inhibitors with Antiproliferative Activity in Cancer Cells

Murat Bozdag[1#], Nabil Mroweh[1#], Alessia Raucci[2#], Andrea Angeli[1], Silvia Peppicelli[3], Alessio Biagioni[3], Lido Calorini[3], Daniela Trisciuoglio[4], Rino Ragno[2], Roberta Astolfi[2], Lidia Giuliani[2], Clemens Zwergel[2], Sergio Valente[2*], Elena Andreucci[3*], Fabrizio Carta[1*] Antonello Mai[2,5¶] and Claudiu T. Supuran[1¶]

[1] Università degli Studi di Firenze, Dipartimento Neurofarba, Sezione di Scienze Farmaceutiche e Nutraceutiche, Via Ugo Schiff 6, I-50019 Sesto Fiorentino (Florence), Italy.

[2] Department of Drug Chemistry & Technologies, Sapienza University of Rome, P. le A Moro 5, Rome 00185, Italy.

[3] Department of Experimental and Clinical Biomedical Sciences "Mario Serio", University of Florence, 50134, Florence, Italy.

[4] Institute of Molecular Biology and Pathology (IBPM), National Research Council (CNR), Rome, 00185, Italy.

[5] Pasteur Institute, Cenci-Bolognetti Foundation, Sapienza University of Rome, Piazzale Aldo Moro 5, 00185 Rome, Italy.

[#] These Authors contributed equally

[¶] These Authors share co-last position

Corresponding Authors

Sergio Valente (S.V.); e-mail: sergio.valente@uniroma1.it

Elena Andreucci (E.A.); e-mail: e.andreucci@unifi.it

Fabrizio Carta (F.C.); e-mail: fabrizio.carta@unifi.it

Index

Synthesis of methyl 4-aminobenzoate (**B**) S5

Synthesis of methyl 4-(4-sulfamoylbenzamido) benzoate (**6**) S5

Synthesis of 4-(4-sulfamoylbenzamido) benzoic acid (**7**) S6

Synthesis of phenyl (4-sulfamoylphenyl)carbamate (**E**) S6

Synthesis of phenyl (3-sulfamoylphenyl)carbamate (**F**) S6-S7

Synthesis of 4-((3-(3-sulfamoylphenyl) ureido) methyl) benzoic acid (**9**) S7

Synthesis of 4-((3-(4-sulfamoylphenyl) ureido) methyl) benzoic acid (**10**) S7

Synthesis of *tert*-butyl (5-fluoro-2-(4-((3-(3-sulfamoylphenyl) ureido) methyl) benzamido) phenyl) carbamate (**13**) S7-S8

Synthesis of t*ert*-butyl (2-amino-5-fluorophenyl)carbamate (**G**) (**Scheme S1**) S8

Synthesis of bis-*tert*-butyl (5-fluoro-2-nitrophenyl)carbamate (**Q**) S8

Synthesis of *tert*-butyl (5-fluoro-2-nitrophenyl)carbamate (**R**) S9

Synthesis of t*ert*-Butyl (2-amino-5-fluorophenyl)carbamate (**G)** S9

Synthesis of methyl 4-((phenoxycarbonyl)amino)benzoate (**H**) S9-S10

Synthesis of methyl 4-(3-(4-sulfamoylphenethyl) ureido) benzoate (**16**) S10

Synthesis of 4-(3-(4-sulfamoylphenethyl) ureido) benzoic acid (**17**) S10

Synthesis of 4-isothiocyanatobenzenesulfonamide (**I**) S10-S11

Synthesis of 3-isothiocyanatobenzenesulfonamide (**J**) S11

Synthesis of 4-(3-(4-sulfamoylphenyl) ureido) benzoic acid (**19**) S11

Synthesis of 4-(3-(3-sulfamoylphenyl) ureido) benzoic acid (**20**) S11-S12

Synthesis of methyl 4-isothiocyanatobenzoate (**K**) S12

Synthesis of methyl 4-(3-(4-sulfamoylbenzyl) thioureido) benzoate (**25**) S12

Synthesis of methyl 4-(3-(4-sulfamoylphenethyl) thioureido) benzoate (**26**) S13

Synthesis of 4-(3-(4-sulfamoylbenzyl) thioureido) benzoic acid (**27**) S13

Synthesis of 4-(3-(4-sulfamoylphenethyl) thioureido) benzoic acid (**28**) S13

Synthesis of methyl 4-((2-ethoxy-2-oxoethyl) amino) benzoate (**L**) S14

Synthesis of (4-(methoxycarbonyl)phenyl) glycine (**M**) S14

Synthesis of methyl 4-((2-oxo-2-((4-sulfamoylbenzyl) amino) ethyl) amino) benzoate (**31**) S14-S15

Synthesis of methyl 4-((2-oxo-2-((4-sulfamoylphenethyl) amino) ethyl) amino) benzoate (**32**) S15

Synthesis of methyl 4-((2-oxo-2-((5-(4-sulfamoylphenyl) pentyl) amino) ethyl) amino) benzoate (**33**) S15

Synthesis of 4-((2-oxo-2-((4-sulfamoylbenzyl) amino) ethyl) amino) benzoic acid (**34**) S15-S16

Synthesis of 4-((2-oxo-2-((4-sulfamoylphenethyl) amino) ethyl) amino) benzoic acid (**35**) S16

Synthesis of 4-((2-oxo-2-((5-(4-sulfamoylphenoxy) pentyl) amino) ethyl) amino) benzoic acid (**36**) S16

Synthesis of *N*-phenyl-4-((3-(4-sulfamoylphenyl)ureido)methyl)benzamide (**11a**) (**Scheme S2**) S17

Synthesis of *N*-phenyl-4-((3-(4-sulfamoylphenyl)ureido)methyl)benzamide (**11a**) S17

Synthesis of *N*-(2-aminophenyl)-4-((3-phenylureido)methyl)benzamide (**11b**) (**Scheme S3**) S17

Synthesis of 4-((3-phenylureido)methyl)benzoic acid (**41**) S17-S18

Synthesis of *N*-(2-aminophenyl)-4-((3-phenylureido)methyl)benzamide (**11b**) S18

Synthesis of *N*-(2-aminophenyl)-4-((2-oxo-2-(phenethylamino)ethyl)amino)benzamide (**40**) (**Scheme S4**) S18

Synthesis of methyl 4-((2-oxo-2-(phenethylamino)ethyl)amino)benzoate (**42**) S18

Synthesis of 4-((2-Oxo-2-(phenethylamino)ethyl)amino)benzoic acid (**43**) S19

Synthesis of *N*-(2-aminophenyl)-4-((2-oxo-2-(phenethylamino)ethyl)amino)benzamide (**40**) S19

^{1}H-, ^{13}C- and ^{19}F-NMR spectra of compounds **, 5, 8, 11, 11a, 11b, 12, 14, 18, 21, 22, 29, 30, 37, 38, 39, 40** S20-S37

Summary of data collection and atomic model refinement statistics for hCA II (**Table S1**) S38

Electron density maps of **37** and **38** within the hCA II active site (**Figure S1**) S39

SAHA and TSA bound conformations in different HDACs (**Figure S2**) S40

List of programs used (**Table S2**) S41-S42

In vitro hCA and HDAC inhibition data of compound **40** (**Table S3** and **Table S4**) S43

Flow cytometry of hCA IX basal expression in HCT-8, HCT-116, MDA-MB-231, BT-474, A375, 501Mel, and Sk-Mel-28 cell lines (**Figure S3**) S44

IC_{50} values of **11** on colon carcinoma cell lines (**A**), mammary carcinoma cell lines (**B**), and melanoma cell lines (**C**) (**Figure S4**) S45

IC_{50} values of **14** on colon carcinoma cell lines (**A**), mammary carcinoma cell lines (**B**), and melanoma cell lines (**C**) (**Figure S5**) S46

Representative plots with relative IC_{50} values of SAHA in HCT-8 and HCT-116 colon carcinoma cell lines, MDA-MB-231 and BT-474 mammary carcinoma cell lines, A375, 501Mel, and Sk-Mel-28 melanoma cell lines, and normal endothelial colony-forming cells (ECFC). (**Figure S6**) S47

Representative dot plots (FL1-H *vs* FL3-H) of HCT116 cells treated for 72h with compound **11** at 5, 10 and 25 μM (**Figure S7**) S48

Representative dot plots (FL1-H *vs* FL3-H) of HCT-116 cells treated for 72h with compound **14** at 5, 10 and 25 μM (**Figure S8**) S49

References S50-S52

Synthesis of methyl 4-aminobenzoate (B)

A solution of 4-aminobenzoic acid (**A**) (3.0 g, 1.0 eq.) in MeOH (30 mL) was cooled to 0°C then treated dropwise with thionyl chloride (5.0 eq.). The mixture was warmed to 65°C for 3h (TLC monitoring), the solvents were removed under reduced pressure and the obtained residue was diluted with H_2O (20 mL), treated with saturated $NaHCO_3$ aqueous solution (40 mL) and extracted with EtOAc (3 x 20 mL). The combined organic layers were washed with H_2O (3 x 20 mL), dried over Na_2CO_3, and evaporated under reduced pressure to obtain the desired compound as a pale-yellow solid. 75% yield; δ_H (400 MHz, DMSO-d_6) 3.77 (3H, s), 5.99 (2H, s, exchange with D_2O, NH_2),6.59 (2H, d, *J* 8.6), 7.66 (2H, d, *J* 8.6); δ_C (100 MHz, DMSO-d_6) 52.0, 113.6, 116.6, 132.0, 154.4, 167.2; *m/z* (ESI positive) 152.09 $[M+H]^+$.

Synthesis of methyl 4-(4-sulfamoylbenzamido) benzoate (6)

To a solution of methyl 4-aminobenzoate (**B**) (1.0 eq.), 4-sulfamoylbenzoic acid (**2**) (1.0 eq.) and HATU (1.3 eq.) in dry DMF (3 mL/0.5 mmol), Et_3N (2.0 eq.) were added under an inert atmosphere. The reaction mixture was stirred at room temperature overnight, then quenched with cold saturated aqueous NH_4Cl solution and stirred for an additional 15 minutes, leading to the formation of a precipitate. The precipitated product was collected by vacuum filtration, washed with water, and triturated with diethyl ether (Et_2O). The crude product was further purified by silica gel column chromatography using ethyl acetate (EtOAc) as the eluent to afford the titled compound as a pale-yellow powder. 70 % yield; δ_H (400 MHz, DMSO-d_6) 3.88 (3H, s), 7.59 (2H, s, exchange with D_2O, SO_2NH_2), 7.97-8.04 (6H, m), 8.15 (2H, d, *J* 8.3), 10.77 (1H, s, exchange with D_2O, N*H*) ; δ_C (100 MHz, DMSO-d_6) 52.9, 120.6, 125.6, 126.7, 129.5, 131.1, 138.4, 144.3, 147.7, 166.0, 166.8. *m/z* (ESI positive) 335.1 $[M+H]^+$.

Synthesis of 4-(4-sulfamoylbenzamido) benzoic acid (7)

Methyl 4-(4-sulfamoylbenzamido) benzoate (**6**) (424 mg, 0.117 mmol, 1.0 eq.) was added to a solution of THF/H_2O/MeOH (3 ml/1.5 ml/3 mL), at room temperature LiOH was added (5.8 mmol, 5.0 eq.). The mixture was stirred overnight and then quenched with 3 N aqueous HCl. After 15 min a precipitate was formed, which was collected by vacuum filtration and washed with water. The obtained solid was triturated with Et_2O to afford compound **7**. 92% yield. δ_H (400 MHz, DMSO-d_6) 7.58 (2H, s, SO_2NH_2), 7.94-8.02 (6H, m), 8.15 (2H, d, *J* 8.3), 10.74 (1H, s, N*H*), 12.82 (1H, brs, COO*H*); δ_C (100 MHz, DMSO-d_6) 120.5, 126.6, 126.8, 129.4, 131.2, 138.4, 143.9, 147.7, 165.9, 167.8. *m/z* (ESI positive) 321.06 $[M+H]^+$

Synthesis of phenyl (4-sulfamoylphenyl)carbamate (E)

Phenylchloroformate (1.5 equiv) was added dropwise at 0 °C to a suspension of 4-sulfamoylaniline (0.5 g, 1 equiv) and K_2CO_3 (1.5 equiv) in acetone (30 mL), and the reaction mixture was stirred for 3 h at RT. The solvent was removed under vacuum, and the residue treated with slush and HCl 6M. The suspension was filtered to obtain **E** as white powder. Yield 95%. δ_H (400 MHz, DMSO-d_6): 10.66 (s, 1H, exchange with D_2O, CONH), 7.80 (d, *J* = 8.3 Hz, 2H, Ar-H), 7.68 (d, *J* = 8.3 Hz, 2H, Ar-H), 7.47 (t, *J* = 8 Hz, 2H Ar-H), 7.29 (m, 5H, exchange with D_2O, SO_2NH_2 + Ar-H), δ_C (100 MHz, DMSO-d_6): 118.0, 121.6, 125.5, 129.1, 129.4, 136.5, 141.2, 150.0, 151.3. Experimental data in agreement with data in literature. [1]

Synthesis of phenyl (3-sulfamoylphenyl)carbamate (F)

Same synthetic pathway of compound (**E**). δ_H(400 MHz, DMSO-d_6): 10.60 (s, 1H, exchange with D2O, CONH), 8.13 (s, 1H, Ar-H), 7.38 (m, 8H, Ar-H), 7.31 (s, 2H, exchange with D_2O, SO_2NH_2, overlapped with signal at 7.38). δ_C (100 MHz, DMSO-d_6): 118.2, 121.4, 125.3, 129.5, 129.8, 136.6, 140.9, 150.5, 151.1. Experimental data in agreement with data in literature. [2]

Synthesis of 4-((3-(3-sulfamoylphenyl) ureido) methyl) benzoic acid (9)

4-(Aminomethyl) benzoic acid (**D**) (248 mg, 1.64 mmol) in ethanol (10 ml) was treated with triethylamine (0.571 ml, 2.5 eq.), followed by *m*-sulfonyl amine carbamate (**F**) (506 mg, 1.85 mmol) in ethanol and reflux at 80 °C for 1 night controlling by TLC the consumption of the starting compounds. Once finished, evaporate the solvent and add water and HCL (1 M) where white precipitate starts to be formed. Filter the white solid and wash with water and then diethyl ether. 45 % yield; δ_H (400 MHz, DMSO-d_6) 4.42 (2H, d, *J* 6), 6.84 (1H, t, *J* 6, exchange with D_2O, N*H*), 7.33 (2H, s, exchange with D_2O, SO_2NH_2), 7.38 (1H, t, *J* 7.8), 7.44 (3H, m), 7.57 (1H, d, *J* 7.8), 7.94 (2H, d, *J* 8.2), 8.05 (1H, s), 9.03 (1H, s, exchange with D_2O, N*H*), 12.93 (1H, brs, exchange with D_2O, COO*H*); δ_C (100 MHz, DMSO-d_6) 43.5, 115.6, 119.1, 121.5, 127.9, 130.1, 130.3, 141.7, 145.5, 146.5, 155.9, 168.1. *m/z* (ESI positive) 350.1 $[M+H]^+$

Synthesis of 4-((3-(4-sulfamoylphenyl) ureido) methyl) benzoic acid (10)

Obtained according to the synthetic procedure for 4-((3-(3-sulfamoylphenyl) ureido) methyl) benzoic acid (**9**) and using 4-(aminomethyl) benzoic acid (**D**)and *p*-sulfonyl amine carbamate (**E**). 51% yield; δ_H (400 MHz, DMSO-d_6) 4.42 (2H, d, *J* 5.9), 6.92 (1H, t, *J* 5.9, exchange with D_2O, N*H*), 7.20 (2H, s, exchange with D_2O, SO_2NH_2), 7.45 (2H, d, *J* 8.1), 7.59 (2H, d, *J* 8.8), 7.71 (2H, d, *J* 8.8), 7.95 (2H, d, *J* 8.1), 9.10 (1H, s, exchange with D_2O, N*H*), 12.90 (1H, brs, exchange with D_2O, COO*H*); δ_C (100 MHz, DMSO-d_6) Aliphatic carbon is beside DMSO signal120.8, 126.9, 127.0, 129.7, 131.5, 138.7, 144.1, 147.8, 166.2, 186.1. *m/z* (ESI positive) 350.1 $[M+H]^+$

Synthesis of *tert*-butyl (5-fluoro-2-(4-((3-(3-sulfamoylphenyl) ureido) methyl) benzamido) phenyl) carbamate (13)

Obtained according to the synthetic procedure for methyl 4-(4-sulfamoylbenzamido) benzoate (**6**) and using *tert*-butyl (2-amino-5-fluorophenyl)carbamate (**G**) and 4-((3-(4-sulfamoylphenyl) ureido) methyl) benzoic acid (**10**). 78% yield; δ_H (400 MHz, DMSO-d_6) 1.48 (9H, s), 4.44 (2H, d, *J* 5.9), 6.86

(1H, t, *J* 5.9, exchange with D_2O, N*H*), 7.00 (1H, m), 7.34 (2H, s, exchange with D_2O, SO_2NH_2), 7.38-7.58 (7H, m), 7.97 (2H, d, *J* 8.1), 8.06 (1H, t, *J* 1.9), 8.80 (1H, s, exchange with D_2O, N*H*), 9.06 (1H, s, exchange with D_2O, N*H*), 9.80 (1H, s, exchange with D_2O, N*H*); δ_C (100 MHz, DMSO-d_6) 28.9, 31.6, 43.5, 81.0, 110.0 (d, $J_{C\text{-}F}$ 25), 111.0 (d, $J_{C\text{-}F}$ 21.1), 115.6, 119.1, 121.5, 125.9, 125.9 (d, $J_{C\text{-}F}$ 3), 127.9, 128.7, 129.2 (d, $J_{C\text{-}F}$ 9), 130.2, 133.5, 135.1 (d, $J_{C\text{-}F}$ 10), 141.8, 145.4, 145.5, 153.9, 156.0, 159.2, 166.5; δ_F (376 MHz, DMSO-d_6) -115.6. *m/z* (ESI positive) 558.1 $[M+H]^+$.

Synthesis of t*ert*-butyl (2-amino-5-fluorophenyl)carbamate (**G**)

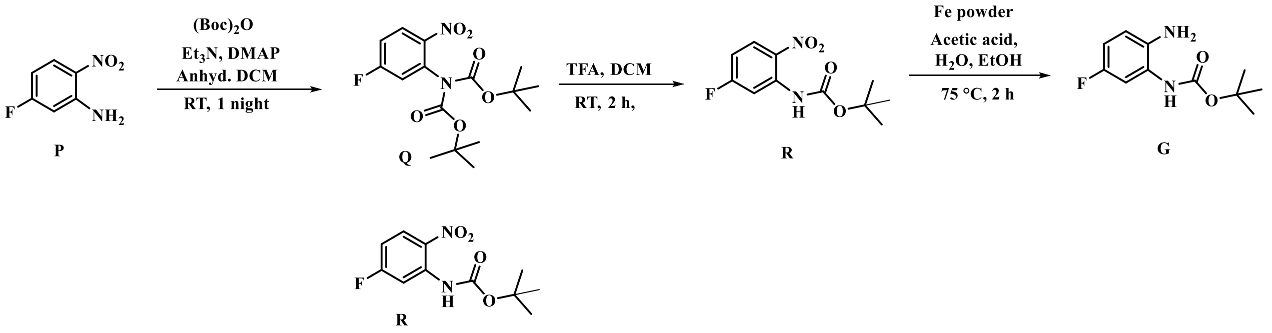

Scheme S1. Multistep synthesis of t*ert*-butyl (2-amino-5-fluorophenyl)carbamate (**G**)

Synthesis of bis-*tert*-butyl (5-fluoro-2-nitrophenyl)carbamate (Q)

At room temperature, to a solution of fluoro-nitroaniline (**P**) (252 mg, 1.684 mmol) in anhydrous CH_2Cl_2 (10 mL), Et_3N (510 mg, 5.06 mmol) was added followed by $(Boc)_2O$ (734 mg, 3.368 mmol) and DMAP (4-dimethylaminopyridine) (2 mg, 0.0168 mmol). After stirring the mixture at room temperature for 1 night, the solution was evaporated under vacuum to get the crude product, which was then subjected to purification by column chromatography (n-Hexane/ethyl acetate: 9/1). First fraction: 150 mg referred to the compound **R**, and the second fraction (400 mg) referred to the compound **Q**. Yield 67 %. δ_H (400 MHz, $CDCl_3$) 1.42 (18H, s), 6.67 (1H, m), 7.20 (1H, m), 8.40 (1H, m*)*. δ_C (100 MHz, $CDCl_3$) 29.5, 78.6, 110.1, 112.5, 118.9, 123.1, 144.3, 155.7.

Synthesis of *tert*-butyl (5-fluoro-2-nitrophenyl)carbamate (R)

A solution of bis-*tert*-butyl (5-fluoro-2-nitrophenyl)carbamate (**Q**) (400 mg, 0.870 mmol), and TFA in DCM was stirred at room temperature for 2 hours. The solution was diluted with ethyl acetate (20 ml), washed with and 10% *v/v* aqueous $NaHSO_4$, dried over Na_2SO_4 concentrated *in vacuo* to give (0.275 g, 96%) as a yellow solid. δ_H (400 MHz, $CDCl_3$) 1.50 (9H, s), 6.74 (1H, m), 8.24 (1H, m), 8.38 (1H, m), 9.87 (1H, s, *NH*). δ_C (100 MHz, $CDCl_3$) 28.4, 80.2, 111.2, 113.5, 126.9, 133.4, 138.3, 153.7, 171.7.

Synthesis of t*ert*-Butyl (2-amino-5-fluorophenyl)carbamate (G)

A solution of *tert*-butyl (5-fluoro-2-nitrophenyl)carbamate (**R**) (275 mg, 1.07 mmol), Iron powder (300 mg, 5.35 mmol) and acetic acid (171 mg, 0.17 ml, 3.21 mmol) in a solvent mixture of water and ethanol (3ml/3ml) was refluxed at 75 °C for 2 hours. Then the reaction mixture was fitered, the organic layer was washed with saturated $NaHCO_3$ solution, dried over Na_2SO_4, and the solvent was evaporated affording the product (204 mg, 84%) as a beige solid. δ_H (400 MHz, DMSO-d_6) 1.46 (9H, s), 4.76 (2H, d, , NH_2), 6.67 (2H, d), 7.20 (1H, d), 8.40 (1H, s, *NH).* δ_C (100 MHz, DMSO-d_6) 29.5, 78.6, 110.1, 112.5, 118.9, 123.1, 144.3, 155.7. Experimental data in agreement with data in literature.[3]

Synthesis of methyl 4-((phenoxycarbonyl)amino)benzoate (H)

To a solution of **10a** (0.45 mmol) in anhydrous DCM (2 mL) under argon. The reaction was cooled to 0 ºC then phenyl chloroformate (0.54 mmol), and pyridine (0.54 mmol) were slowly added. The reaction was allowed to rise to room temperature and was stirred for 1.5 h. After being quenched by the addition of 1 N HCl and water, the aqueous layer was extracted with EtOAc (2 × 15 mL). The combined organic extracts were washed with brine, dried over anhydrous Na_2SO_4, filtered, and concentrated. The residue purified by column chromatography on silica gel (EtOAc/hexane, 10:90) to give **H** (96%) as a white solid; ^{1}H NMR ($CDCl_3$, 500 MHz) 7.47 (1 H, s), 7.40–7.33 (3 H, m),

7.29–7.22 (2 H, m), 7.18 (2 H, d, J = 7.4 Hz), 7.13 (1 H, br), 7.06 (1 H, d, J = 7.4 Hz), 3.65 (3 H, s), 1.57 (6 H, s); δ_C (CDCl3, 125 MHz) 177.1, 151.6, 150.4, 145.8, 137.5, 129.4, 129.1, 125.7, 121.6, 121.2, 117.1, 116.1, 52.3, 46.5, 26.4. Experimental data in agreement with data in literature. [4]

Synthesis of methyl 4-(3-(4-sulfamoylphenethyl) ureido) benzoate (16)

A mixture of methyl 4-((phenoxy carbonyl)amino)benzoate (**H**) (1.0 eq.) and 4-(aminoethyl) phenylsulfonyl amine (**15**) (1.0 eq.) in acetonitrile (ACN) (10 mL) was stirred at reflux overnight. Then water was added, and the precipitate was filtered off and dried under vacuum as white powder of 82% yield; δ_H (400 MHz, DMSO-d_6) 2.88 (2H, t, *J* 7), 3.42 (2H, q, *J* 7), 3.83 (3H, s), 6.35 (1H, t, *J* 7, exchange with D_2O, N*H*), 7.33 (2H, s, exchange with D_2O, SO_2NH_2), 7.46 (2H, d, *J* 8.4), 7.55 (2H, d, *J* 8.8), 7.81 (2H, d, *J* 8.4), 7.86 (2H, d, *J* 8.8), 8.96 (1H, s, exchange with D_2O, N*H*); δ_C (100 MHz, DMSO-d_6) 36.3, 41.2, 52.6, 117.6, 122.6, 126.7, 130.1, 131.3, 143.0, 144.6, 146.0, 155.6, 166.9. *m/z* (ESI positive) 378.07 $[M+H]^+$

Synthesis of 4-(3-(4-sulfamoylphenethyl) ureido) benzoic acid (17)

Obtained according to the synthetic procedure for 4-(4-sulfamoylbenzamido) benzoic acid (**7**) using 4-(3-(4-sulfamoylphenethyl) ureido) benzoate (**16**). 78% yield; δ_H (400 MHz, DMSO-d_6) 2.84 (2H, t, *J* 7), 3.39 (2H, q, *J* 7), 6.32 (1H, t, *J* 7, exchange with D_2O, N*H*), 7.31 (2H, s, exchange with D_2O, SO_2NH_2), 7.41 (2H, d, *J* 8.4), 7.52 (2H, d, *J* 8.8), 7.77 (2H, d, *J* 8.4), 7.83 (2H, d, *J* 8.8), 8.92 (1H, s, exchange with D_2O, N*H*), 12.75 (1H, s, exchange with D_2O, COO*H*); δ_C (100 MHz, DMSO-d_6) 36.4, 41.2, 117.5, 123.7, 126.7, 130.1, 131.4, 143.0, 144.6, 145.7, 155.7, 168.0. *m/z* (ESI positive) 364.13 $[M+H]^+$

Synthesis of 4-isothiocyanatobenzenesulfonamide (I)

Thiophosgene (4.3 mmol) was added dropwise at 0 °C to a solution of sulfanilamide (4.3 mmol) in HCl 1M (5 mL), and the mixture was stirred at r.t. for 3h. After that, the suspension was quenched

with slush and HCl 1M, and the resulting precipitate was filtered and washed with H_2O to give **I** in high yield and purity as a yellow powder. Yield 94%; silica gel TLC Rf 0.83 (MeOH/DCM 10% *v*/*v*); δ_H (400 MHz, DMSO-d6): 7.88 (d, *J* = 8.3 Hz, 2H, Ar-*H*), 7.63 (d, J = 8.3 Hz, 2H, Ar-*H*), 7.52 (s, 2H, exchange with D_2O, SO_2NH_2); δ_C (100 MHz, DMSO-d6): 142.7, 136.9, 134.7, 128.6, 124.6. Experimental data in agreement with data in literature. [1]

Synthesis of 3-isothiocyanatobenzenesulfonamide (J)

Same procedure as compound **I**. 81% yield; silica gel TLC R_f 0.46 (MeOH/DCM 10% *v/v*); δ_H (400 MHz, DMSO-*d*6) 7.48 (2H, s, exchange with D_2O, SO_2NH_2), 7.63 (1H, d, *J* 8.4, Ar-H), 7.68 (1H, dd, *J* 8.4, Ar-H), 7.89 (1H, d, *J* 8.4, Ar-H), 7.92(1H,s,ArH); δ_C (100 MHz,DMSO-*d*6) 123.0, 124.2, 129.7, 129.9, 130.1, 132.0, 142.0. Experimental data in agreement with data in literature. [5]

Synthesis of 4-(3-(4-sulfamoylphenyl) ureido) benzoic acid (19)

A mixture of 4-isothiocyanatobenzenesulfonamide (**I**) (1.0 eq.), 4-aminobenzoic acid (**A**) (1.0 eq.) and triethylamine (2.5 eq.) in acetonitrile (ACN) (10 mL) was stirred at reflux overnight. Then water was added, and the precipitate was filtered off and dried under vacuum as a white powder 79 % yield; δ_H (400 MHz, DMSO-*d*6) 7.34 (2H, s, exchange with D_2O, SO_2NH_2), 7.70-7.82 (6H, m), 7.95 (2H, d, *J* 8.6), 10.56 (2H, m, exchange with D_2O, 2 x N*H*), 12.63 (1H, brs, exchange with D_2O, COO*H*); δ_C (100 MHz, DMSO-*d*6) 113.6, 123.0, 123.7, 127.2, 130.9, 140.2, 143.5, 144.4, 168.3, 180.3. *m/z* (ESI positive) 352.09 $[M+H]^+$

Synthesis of 4-(3-(3-sulfamoylphenyl) ureido) benzoic acid (20)

Obtained according to the synthetic procedure for 4-(3-(4-sulfamoylphenyl) ureido) benzoic acid (**19**) by using 3-isothiocyanatobenzenesulfonamide (**J**) (1.0 eq.) and 4-aminobenzoic acid (**A**) (1.0 eq.). 72 % yield; δ_H (400 MHz, DMSO-*d*6) 7.43 (2H, s, exchange with D_2O, SO_2NH_2), 7.57 (1H, t, *J* 7.8), 7.63 (1H, d, *J* 7.8), 7.68 (2H, d, *J* 8.7), 7.77 (1H, d, *J* 7.8), 7.95 (2H, d, J 8.7), 8.02 (1H, s); δ_C (100

MHz, DMSO-d_6) 121.8, 122.8, 123.3, 127.2, 128.1, 130.2, 131.1, 140.9, 144.6, 145.4, 168.1, 180.8. *m/z* (ESI positive) 352.1 $[M+H]^+$

Synthesis of methyl 4-isothiocyanatobenzoate (K)

A 50 mL round-bottomed flask was charged with methyl 4-aminobenzoate (5.5 mmol), THF (5 mL), and sodium hydride (60% in mineral oil; 16.5 mmol) in an ice bath under N_2 atmosphere. CS_2 (16.5 mmol) was added via syringe pump as the reaction was brought up to room temperature over 1 h. The mixture was then refluxed at 75 °C for 20 h. The mixture was then cooled on an ice bath, and TsCl (6.1 mmol) and Et_3N (13 mmol) were added. The mixture was stirred at room temperature for 0.5 h. After 0.5 h, 1 N HCl (5 mL) added to the mixture. The compound was extracted by EtOAc (10 mL). The organic layers were then combined, dried over Na_2SO_4, and filtered. The filtrate was concentrated in vacuo to obtain an oil, which was passed through a silica column with 100% hexane as eluent. Yield 89%. δ_H (400 MHz, DMSO-d_6) δ 7.99 (d, J = 8.2 Hz, 2H) 7.54 (d, J = 8.2 Hz, 2H) 3.85 (s, 3H). δ_C (100 MHz, DMSO-d_6) δ 165.0 135.9 134.4 130.6 128.4 126.1 52.3. Experimental data in agreement with data in literature. [6]

Synthesis of methyl 4-(3-(4-sulfamoylbenzyl) thioureido) benzoate (25)

Obtained according to the synthetic procedure for methyl 4-(3-(4-sulfamoylphenethyl) ureido) benzoate (**16**) using **23** and **K**. 88% yield; δ_H (400 MHz, DMSO-d_6) 3.82 (3H, s), 4.83 (2H, d, *J* 5.7), 7.31 (2H, s, exchange with D_2O, SO_2NH_2), 7.51 (2H, d, *J* 8.4), 7.65 (2H, d, *J* 8.7), 7.80 (2H, d, *J* 8.4), 7.88 (2H, d, *J* 8.7), 8.53 (1H, t, *J* 5.7, exchange with D_2O, N*H*), 10.01 (1H, s, exchange with D_2O, N*H*); δ_C (100 MHz, DMSO-d_6) 47.6, 52.8, 122.2, 125.2, 126.6, 128.5, 130.8, 143.6, 143.8, 145.0, 166.7, 181.7. *m/z* (ESI positive) 380.2 $[M+H]^+$

Synthesis of methyl 4-(3-(4-sulfamoylphenethyl) thioureido) benzoate (26)

Obtained according to the synthetic procedure for methyl 4-(3-(4-sulfamoylphenethyl) ureido) benzoate (**16**) using **24** and **K**. 75% yield; δ_H (400 MHz, DMSO-d_6) 3.02 (2H, t, *J* 7.2), 3.80 (2H, q, *J* 7.2), 3.86 (3H, s), 7.33 (2H, s, exchange with D_2O, SO_2NH_2), 7.50 (2H, d, *J* 8.3), 7.64 (2H, d, *J* 8.8), 7.81 (2H, d, *J* 8.3), 7.90 (2H, d, *J* 8.8), 8.12 (1H, brs, exchange with D_2O, N*H*), 9.92 (1H, brs, exchange with D_2O, N*H*); δ_C (100 MHz, DMSO-d_6) 34.8, 45.7, 52.8, 121.8, 126.7, 130.1, 130.8, 143.1, 144.3, 145.0, 166.7, 181.1. *m/z* (ESI positive) 394.1 $[M+H]^+$

Synthesis of 4-(3-(4-sulfamoylbenzyl) thioureido) benzoic acid (27)

Obtained according to the synthetic procedure for 4-(4-sulfamoylbenzamido) benzoic acid (**7**) using 4-(3-(4-sulfamoylbenzyl) thioureido) benzoate (**25**). 98% yield; δ_H (400 MHz, DMSO-d_6) 4.86 (2H, d, *J* 5.7), 7.35 (2H, s, exchange with D_2O, SO_2NH_2), 7.54 (2H, d, *J* 8.4), 7.68 (2H, d, *J* 8.7), 7.83 (2H, d, *J* 8.4), 7.92 (2H, d, *J* 8.7), 8.59 (1H, t, *J* 5.7, exchange with D_2O, N*H*), 10.08 (1H, s, exchange with D_2O, N*H*), 12.75 (1H, brs, exchange with D_2O, COO*H*); δ_C (100 MHz, DMSO-d_6) 47.6, 122.3, 126.5, 126.6, 128.5, 130.9, 143.6, 143.8, 144.5, 167.8, 181.8. *m/z* (ESI positive) 366.08 $[M+H]^+$

Synthesis of 4-(3-(4-sulfamoylphenethyl) thioureido) benzoic acid (28)

Obtained according to the synthetic procedure for 4-(4-sulfamoylbenzamido) benzoic acid (**7**) using 4-(3-(4-sulfamoylphenethyl) thioureido) benzoate (**26**). 63% yield; δ_H (400 MHz, DMSO-d_6) 3.02 (2H, t, *J* 7), 3.79 (2H, q, *J* 7), 7.33 (2H, s, exchange with D_2O, SO_2NH_2), 7.50 (2H, d, *J* 8.4), 7.62 (2H, d, *J* 8.7), 7.81 (2H, d, J 8.4), 7.90 (2H, d, *J* 8.7), 8.18 (1H, t, *J* 7, exchange with D_2O, N*H*), 10.01 (1H, s, exchange with D_2O, N*H*), 12.72 (1H, s, exchange with D_2O, COO*H*); δ_C (100 MHz, DMSO-d_6) 34.9, 45.7, 121.8, 126.1, 126.7, 130.1, 130.9, 143.1, 144.4, 144.7, 167.9, 181.2. *m/z* (ESI positive) 380.1 $[M+H]^+$

Synthesis of methyl 4-((2-ethoxy-2-oxoethyl) amino) benzoate (L)

A solution of methyl 4-aminobenzoate (**B**) (2.45g, 1.0 eq.) in dry DMF was treated with K_2CO_3 (2.0 eq.), then ethyl iodoacetate (1.1 eq.) was added to the reaction mixture and heated to 100˚C for one night. After the consumption of the starting materials (TLC monitoring), the reaction was cooled to r.t. and quenched with H_2O, neutralized with a 1M HCl aqueous solution to obtain a precipitate which was filtered off, washed with H_2O and dried under vacuum to afford the titled compound as pale-yellow solid. 75% yield; δ_H (400 MHz, DMSO-*d*6) 1.23 (3H, t, *J* 7.1), 3.78 (3H, s), 4.01 (2H, d, *J* 6.3), 4.16 (2H, q, *J* 7.1), 6.6 (2H, d, *J* 8.88), 6.90 (1H, t, *J* 6.3, exchange with D_2O, N*H*), 7.73 (2H, d, *J* 8.88); δ_C (100 MHz, DMSO-*d*6) 15.0, 45.0, 52.2, 61.4, 112.2, 117.6, 131.8, 153.2, 167.2, 171.5. *m/z* (ESI positive) 238.14 $[M+H]^+$

Synthesis of (4-(methoxycarbonyl)phenyl) glycine (M)

A solution of methyl 4-((2-ethoxy-2-oxoethyl)amino)benzoate (**L**) in EtOH was treated with 5% NaOH in H_2O, the reaction continued until the consumption of the starting materials (TLC monitoring) then quenched with 1M HCl aqueous solution. Obtained precipitate was filtered off and washed with H_2O (3 x 5 mL) and dried under vacuum to obtain desired compound as a white solid. 69% yield; δ_H (400 MHz, DMSO-*d*6) 3.78 (3H, s), 3.92 (2H, m), 6.62 (2H, d, *J* 8.8), 6.80 (1H, t, *J* 5.5, exchange with D_2O, N*H*), 7.73 (2H, d, *J* 8.88), 12.71 (1H, brs, exchange with D_2O, COO*H*); δ_C (100 MHz, DMSO-*d*6) 45.0, 52.1, 112.1, 117.4, 131.7, 153.3, 167.2, 172.8. *m/z* (ESI positive) 209.9 $[M+H]^+$

Synthesis of methyl 4-((2-oxo-2-((4-sulfamoylbenzyl) amino) ethyl) amino) benzoate (31)

Obtained according to the synthetic procedure for methyl 4-(4-sulfamoylbenzamido) benzoate (**6**) using of (4-(methoxycarbonyl)phenyl) glycine (**M**) and 4-(aminomethyl)benzenesulfonamide (**N**). 80% yield; δ_H (400 MHz, DMSO-*d*6) 3.79 (3H, s), 3.85 (2H, d, *J* 6), 4.39 (2H, d, *J* 5.9), 6.64 (2H, d,

J 8.8), 6.89 (1H, t, *J* 5.9), 7.34 (2H, s, exchange with D_2O, SO_2NH_2), 7.42 (2H, d, *J* 8.2), 7.75-7.79 (4H, m), 8.63 (1H, t, *J* 6) ; δ_C (100 MHz, DMSO-d_6) 42.7, 47.1, 52.1 112.2, 118.8, 126.5, 128.3, 131.9, 143.5, 144.5, 153.1, 168.3, 170.8. *m/z* (ESI positive) 378.1 $[M+H]^+$

Synthesis of methyl 4-((2-oxo-2-((4-sulfamoylphenethyl) amino) ethyl) amino) benzoate (32)

Obtained according to the synthetic procedure for methyl 4-(4-sulfamoylbenzamido) benzoate (**6**) using of (4-(methoxycarbonyl)phenyl) glycine (**M**) and 4-(2-aminoethyl)benzenesulfonamide (**15**). 58% yield; δ_H (400 MHz, DMSO-d_6) 2.81 (2H, t, *J* 7), 3.37 (2H, m), 3.73 (2H, d, *J* 6), 3.79 (3H, s), 6.59 (2H, d, *J* 8.8), 6.82 (1H, t, *J* 7, exchange with D_2O, N*H*), 7.32 (2H, s, exchange with D_2O, SO_2NH_2), 7.39 (2H, d, *J* 8.5), 7.73-7.76 (4H, m), 8.10 (1H, t, *J* 6, exchange with D_2O, N*H*); δ_C (100 MHz, DMSO-d_6) 35.7, 41.1, 46.9, 52.1, 112.3, 117.5, 126.6, 130.0, 131.7, 143.0, 144.5, 153.4, 167.2, 170.2. *m/z* (ESI positive) 392.08 $[M+H]^+$

Synthesis of methyl methyl 4-((2-oxo-2-((5-(4-sulfamoylphenoxy)pentyl)amino)ethyl)amino)benzoate (33)

Obtained according to the synthetic procedure for methyl 4-(4-sulfamoylbenzamido) benzoate (**6**) using of (4-(methoxycarbonyl)phenyl) glycine (**M**) and 4-((5-aminopentyl)oxy)benzene sulfonamide (**O**). 70% yield; δ_H (400 MHz, DMSO-d_6) 1.36-1.53 (4H, m), 1.73 (2H, pent, *J* 6.8), 3.14 (2H, q, *J* 6.8), 3.74 (2H, d, *J* 6), 3.76 (3H, s), 4.02 (2H, t, *J* 6.8), 6.60 (2H, d, *J* 8.8), 6.83 (1H, t, *J* 6.8, exchange with D_2O, N*H*), 7.08 (2H, d, *J* 8.9), 7.73-7.77 (4H, m), 8.00 (1H, t, *J* 6); δ_C (100 MHz, DMSO-d_6) 23.13, 28.46, 29.01, 38.76, 46.59, 52.60, 68.28, 111.79, 113.25, 115.02, 118.37, 128.08, 131.45, 136.16, 152.56, 161.51, 168.06, 170.19. *m/z* (ESI positive) 434.13 $[M+H]^+$

Synthesis of 4-((2-oxo-2-((4-sulfamoylbenzyl) amino) ethyl) amino) benzoic acid (34)

Obtained according to the synthetic procedure for 4-(4-sulfamoylbenzamido) benzoic acid (**7**) using methyl 4-((2-oxo-2-((4-sulfamoylbenzyl) amino) ethyl) amino) benzoate (**31**). 80% yield; δ_H (400

MHz, DMSO-*d*$_6$) 3.84 (2H, m), 4.40 (2H, d, *J* 5.9), 6.62 (2H, d, *J* 8.8), 6.81 (1H, brs, exchange with D_2O, N*H*), 7.33 (2H, s, exchange with D_2O, SO_2NH_2), 7.43 (2H, d, *J* 8.3), 7.73 (2H, d, *J* 8.8), 7.77 (2H, d, *J* 8.3), 8.62 (1H, t, *J* 5.9, exchange with D_2O, N*H*), 12.18 (1H, brs, exchange with D_2O, COO*H*); δ_C (100 MHz, DMSO-*d*$_6$) 42.7, 47.1, 112.2, 118.8, 126.5, 128.3, 131.9, 143.5, 144.5, 153.1, 168.3, 170.8. *m/z* (ESI positive) 464.1 $[M+H]^+$

Synthesis of 4-((2-oxo-2-((4-sulfamoylphenethyl) amino) ethyl) amino) benzoic acid (35)

Obtained according to the synthetic procedure for 4-(4-sulfamoylbenzamido) benzoic acid (**7**) using methyl 4-((2-oxo-2-((4-sulfamoylphenethyl) amino) ethyl) amino) benzoate (**32**). 55% yield; δ_H (400 MHz, DMSO-*d*$_6$) 2.82 (2H, t, *J* 7.2), 3.39 (2H, m), 3.72 (2H, d, *J* 6), 6.57 (2H, d, *J* 8.7), 6.72 (1H, t, *J* 7.2, exchange with D_2O, N*H*), 7.32 (2H, s, exchange with D_2O, SO_2NH_2), 7.40 (2H, d, *J* 8.4), 7.72 (2H, d, *J* 8.7), 7.75 (2H, d, *J* 8.4), 8.09 (1H, t, *J* 6, exchange with D_2O, N*H*), 12.10 (1H, s, exchange with D_2O, COO*H*); δ_C (100 MHz, DMSO-*d*$_6$) 35.9, 40.8, 47.2, 112.3, 118.9, 126.7, 130.2, 132.1, 143.0, 144.7, 153.2, 168.6, 170.6. *m/z* (ESI positive) 477.9 $[M+H]^+$

Synthesis of 4-((2-oxo-2-((5-(4-sulfamoylphenoxy) pentyl) amino) ethyl) amino) benzoic acid (36)

Obtained according to the synthetic procedure for 4-(4-sulfamoylbenzamido) benzoic acid (**7**) using methyl 4-((2-oxo-2-((5-(4-sulfamoylphenyl) pentyl) amino) ethyl) amino) benzoate (**33**). 80% yield; δ_H (400 MHz, DMSO-*d*$_6$) 1.35-1.53 (4H, m), 1.74 (2H, pent, *J* 7), 3.14 (2H, q, *J* 6.6), 3.72-3.79 (2H, m), 4.02 (2H, m), 6.60 (2H, m), 6.74 (1H, t, *J* 6, exchange with D_2O, N*H*), 7.08 (2H, m), 7.22 (2H, m, exchange with D_2O, SO_2NH_2), 7.70-7.78 (4H, m), 8.00 (1H, m, exchange with D_2O, N*H*), 12.13 (1H, brs, exchange with D_2O, COOH); δ_C (100 MHz, DMSO-*d*$_6$) 23.1, 28.5, 29.0, 38.7, 46.6, 68.3, 111.8, 113.2, 115.0, 118.4, 128.1, 131.5, 136.2, 152.5, 161.5, 168.0, 170.2. *m/z* (ESI positive) 436.11 $[M+H]^+$

Synthesis of *N*-phenyl-4-((3-(4-sulfamoylphenyl)ureido)methyl)benzamide (11a)

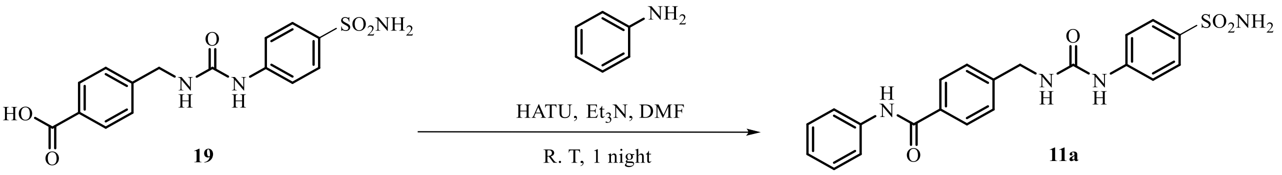

Scheme S2. Synthesis of compound 11a

Obtained according to the synthetic procedure for methyl 4-(4-sulfamoylbenzamido) benzoate (**6**) using 4-((3-(3-sulfamoylphenyl) ureido) methyl) benzoic acid (**19**) and aniline. 36% yield. δ_H (400 MHz, DMSO-d_6): 4.38 (2H, d, *J* 6.0), 6.85 (1H, t, *J* 6.1), 7.08 (1H, t, *J* 7.5), 7.13 (2H, s), 7.33 (1H, t, *J* 8.2), 7.44 (2H, d, *J* 8.2), 7.55 (2H, d, *J* 8.9), 7.67 (1H, d, *J* 8.5), 7.75 (1H, d, *J* 8.0), 7.91 (2H, d, *J* 8.0), 9.02 (1H, s, N*H*), 10.16 (1H, s, N*H*). δ_C (100 MHz, DMSO-d_6) 43.6, 118.0, 121.4, 124.6, 127.8, 128.0, 128.8, 129.6, 134.5, 137.2, 140.2, 144.6, 145.0, 155.9, 166.3; *m/z* (ESI positive) 425.06 $[M+H]^+$. Elemental analysis calculated (%): C 59.42, H 4.75, N 13.20, O 15.08, S 7.55; found: C 59.56, H 4.81, N 13.27.

Synthesis of *N*-(2-aminophenyl)-4-((3-phenylureido)methyl)benzamide (11b)

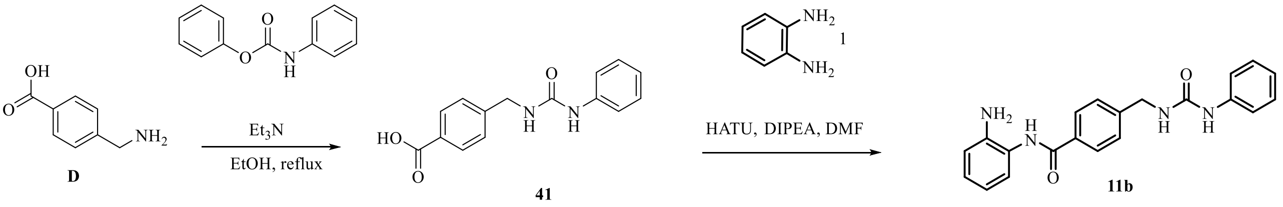

Scheme S3. Synthesis of compound 11b

Synthesis of 4-((3-phenylureido)methyl)benzoic acid (41)

Obtained according to the synthetic procedure for compound **19** using 4-(aminomethyl)benzoic acid (**D**) and phenyl phenylcarbamate. 45 % yield; δ_H (400 MHz, DMSO-d_6) 4.42 (2H, d, *J* 6), 6.74 (1H, t, *J* 5.8, exchange with D_2O, N*H*), 6.92 (1H, t, *J* 7.3, exchange with D_2O, N*H*), 7.22 (2H, t, *J* 7.8),

7.44 (4H, d, *J* 8.6), 7.89 (2H, d, *J* 8.3), 8.63 (1H, s), 12.77 (1H, br s, COO*H*); δ_C (100 MHz, DMSO-d_6) 42.5, 127.1, 128.4, 129.5, 131.1, 141.2, 153.8, 156.9, 157.7, 166.6; *m/z* (ESI negative) 269.12 [M-H]$^-$.

Synthesis of *N*-(2-aminophenyl)-4-((3-phenylureido)methyl)benzamide (11b)

Obtained according to the synthetic procedurefor ***N***-(2-aminophenyl)-4-sulfamoylbenzamide (**4**) using of 4-((3-phenylureido)methyl)benzoic acid (**40**) and *o*-amino aniline (**1**). 55% yield. δ_H (400 MHz, DMSO-d_6): 4.37 (2H, d, *J* 6.0), 4.86 (2H, brs, exchange with D_2O, NH_2), 6.66 (1H, t, *J* 6.0), 6.77 (1H, d, *J* 8.2), 6.88 (1H, t, *J* 7.5), 6.94 (1H, t, *J* 8.0), 7.16 (2H, d, *J* 8.0), 7.20 (2H, t, *J* 8.2), 7.40 (3H, m), 7.93 (2H, d, *J* 8.3), 8.58 (1H, s, exchange with D_2O, N*H*), 9.59 (1H, s, exchange with D_2O, N*H*). δ_C (100 MHz, DMSO-d$_6$) 43.6, 117.2, 117.3, 118.8, 122.2, 124.4, 127.9, 128.9, 129.7, 134.1, 141.5, 144.2, 145.1, 156.3, 166.2; *m/z* (ESI positive) 361.05 [M+H] $^+$. Elemental analysis calculated (%): C 69.98, H 5.59, N 15.55, O 8.88; found: C 67.10, H 5.71, N 15.49.

Synthesis of *N*-(2-aminophenyl)-4-((2-oxo-2-(phenethylamino)ethyl)amino)benzamide (40)

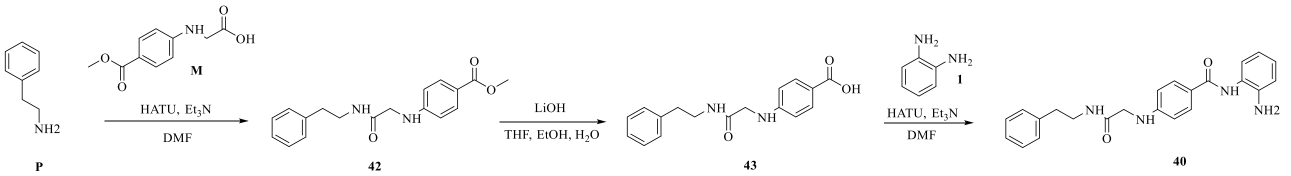

Scheme S4. Synthesis of compound 40

Methyl 4-((2-oxo-2-(phenethylamino)ethyl)amino)benzoate (42)

Following the general procedure of the synthesis of compounds **31-33**. 70% yield ; δ_H (400 MHz, DMSO-d_6) 2.74 (2H, t, *J* 7.2), 3.36 (2H, m), 3.72 (2H, d, *J* 6), 3.79 (3H, s), 6.58 (2H, d, *J* 8.8), 6.80 (1H, t, *J* 6, exchange with D_2O, N*H*), 7.19-7.31 (5H, m), 7.74 (2H, d, *J* 8.8), 8.03 (1H, t, *J* 5.7, exchange with D_2O, N*H*); δ_C (100 MHz, DMSO-d_6) 36.0, 41.0, 47.0, 52.2, 112.3, 117.5, 127.0, 129.2, 129.5, 131.8, 140.3, 153.4, 167.2, 170.2

4-((2-Oxo-2-(phenethylamino)ethyl)amino)benzoic acid (43)

Following the same synthesis procedure of compounds **34-36**. 63% yield; δ_H (400 MHz, DMSO-d_6) 2.74 (2H, t, *J* 7.2), 3.33 (2H, q, *J* 7.2), 3.71 (2H, d, *J* 6), 6.56 (2H, d, *J* 8.8), 6.71 (1H, t, *J* 7.2, exchange with D_2O, N*H*), 7.19-7.24 (3H, m), 7.28-7.32 (2H, m), 7.71 (2H, d, *J* 8.8), 8.02 (1H, t, *J* 6, exchange with D_2O, N*H*), 12.07 (1H, brs, exchange with D_2O, COO*H*); δ_C (100 MHz, DMSO-d_6) 36.1, 41.0, 47.1, 112.2, 118.9, 126.9, 129.2, 129.5, 131.9, 140.1, 153.2, 168.4, 170.3.

***N*-(2-Aminophenyl)-4-((2-oxo-2-(phenethylamino)ethyl)amino)benzamide (40)**

Following the general procedure of the synthesis of compounds **37-39**. 60% yield; δ_H (400 MHz, DMSO-d_6) 2.75 (2H, t, *J* 7.2), 3.37 (2H, m), 3.73 (2H, d, *J* 6), 4.85 (2H, s, exchange with D_2O, NH_2), 6.57 (1H, t, *J* 6.1, N*H*), 6.59-6.65 (3H, m), 6.80 (1H, dd, *J* 1.6, 8), 6.98 (1H, ddd, *J* 1.6, 8), 7.17-7.24 (4H, m), 7.30-7.33 (2H, m), 7.81 (2H, d, *J* 8.8), 8.03 (1H, t, *J* 5.2, exchange with D_2O, N*H*), 9.35 (1H, s, exchange with D_2O, N*H*); δ_C (100 MHz, DMSO-d_6) 36.1, 41.1, 47.2, 112.1, 117.1, 117.3, 122.7, 125.1, 126.9, 127.0, 127.3, 129.2, 129.5, 130.1, 140.3, 143.9, 152.0, 166.0, 170.4; *m/z* (ESI positive) 389 $[M+H]^+$. Elemental analysis calculated (%): C 71.11; H, 6.23; N, 14.42; found: C 71.15, H 6.25, N 14.38.

^{1}H- ^{13}C- and ^{19}F-NMR Spectra of compounds

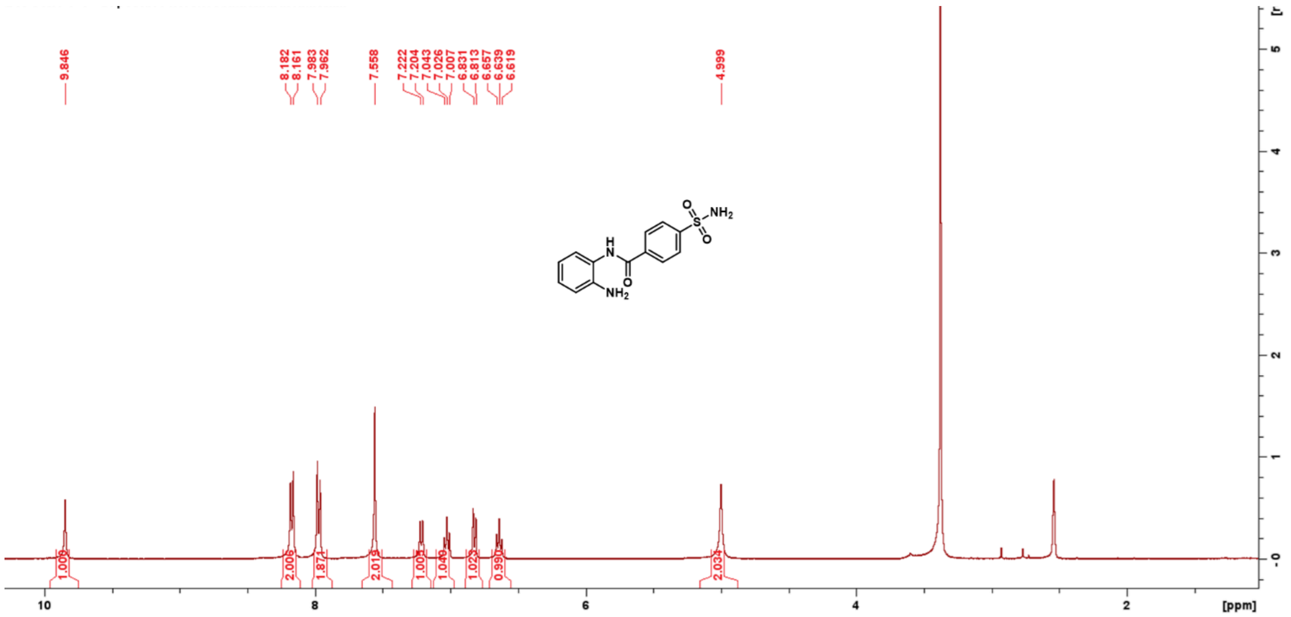

^{1}H NMR spectrum of compound **4** (400 MHz, DMSO-d$_6$)

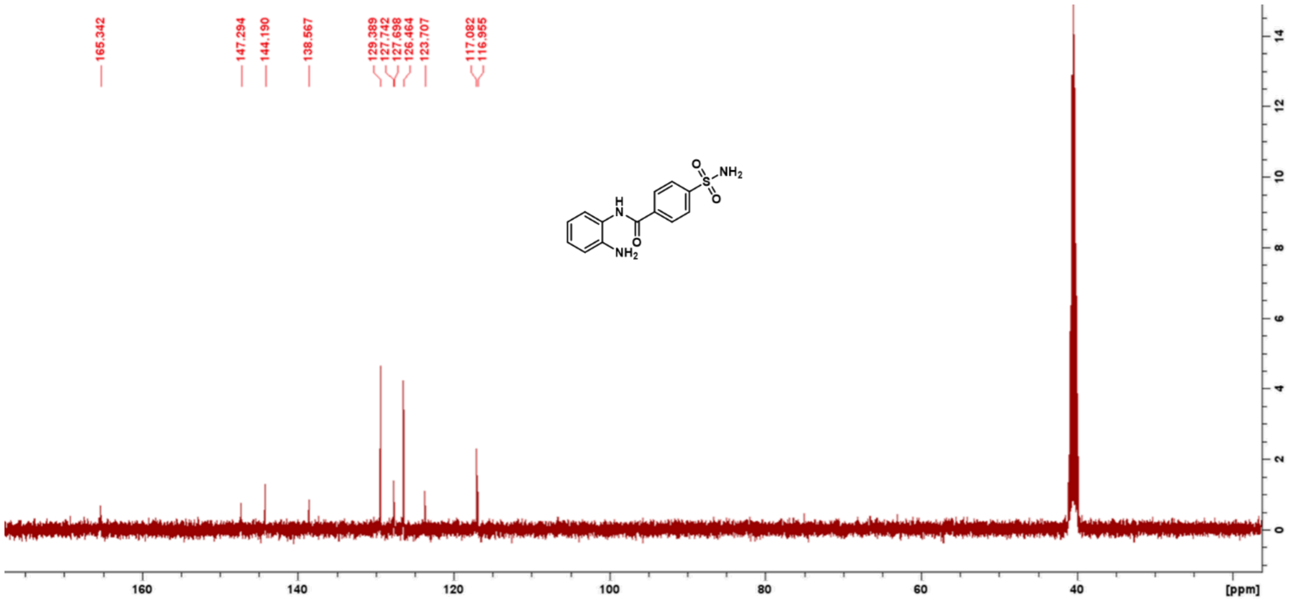

^{13}C NMR spectrum of compound **4** (400 MHz, DMSO-d$_6$)

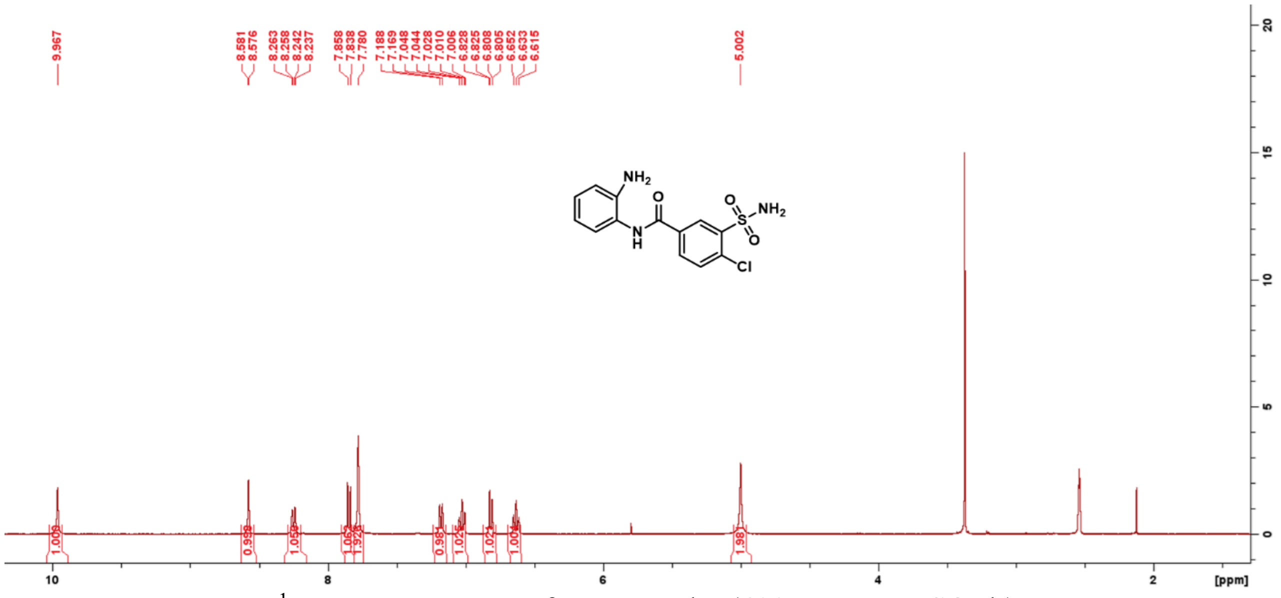

^{1}H NMR spectrum of compound **5** (400 MHz, DMSO-d$_6$)

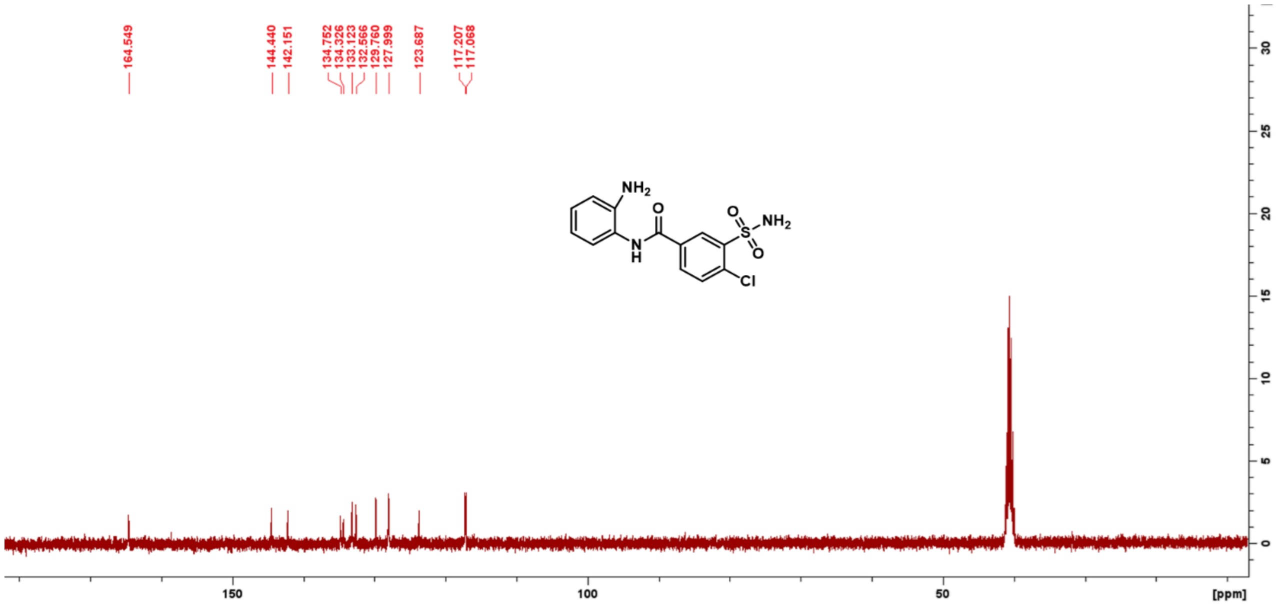

^{13}C NMR spectrum of compound **5** (400 MHz, DMSO-d$_6$)

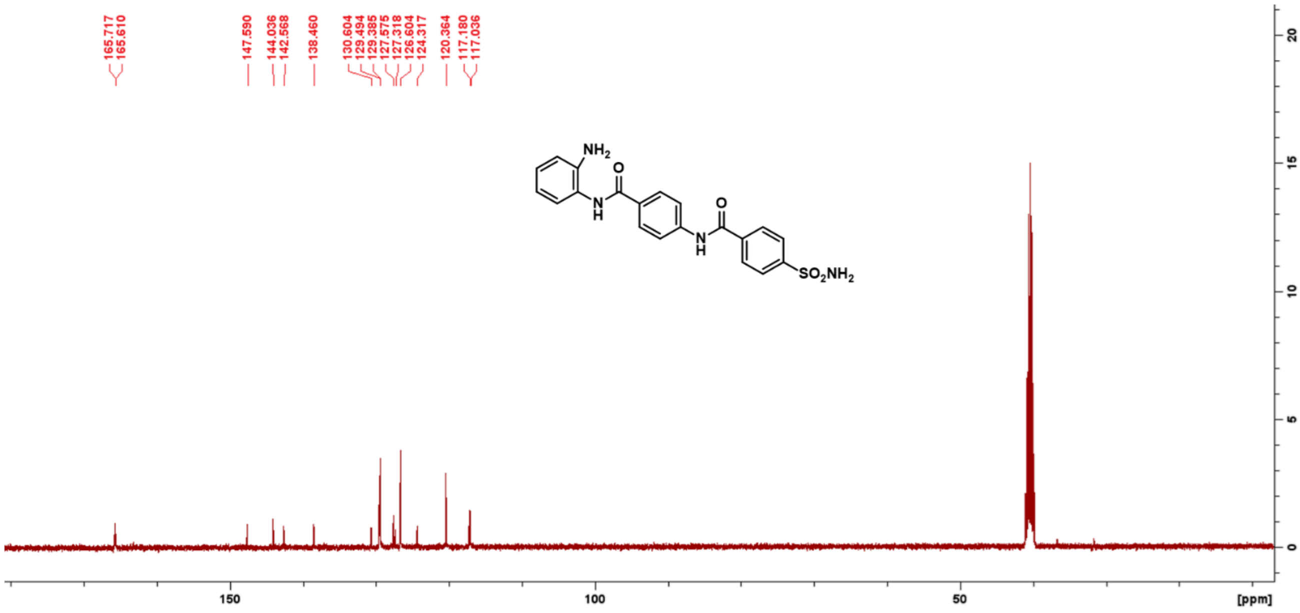

^{13}C NMR spectrum of compound **8** (400 MHz, DMSO-d_6)

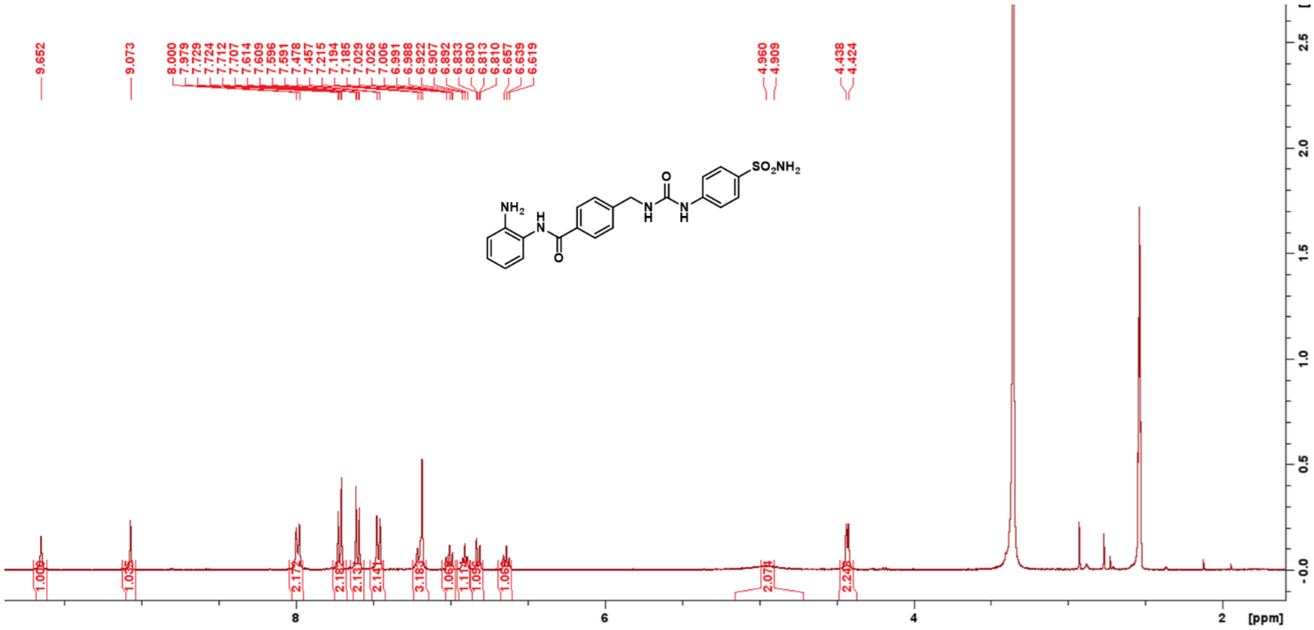

^{1}H NMR spectrum of compound **11** (400 MHz, DMSO-d$_6$

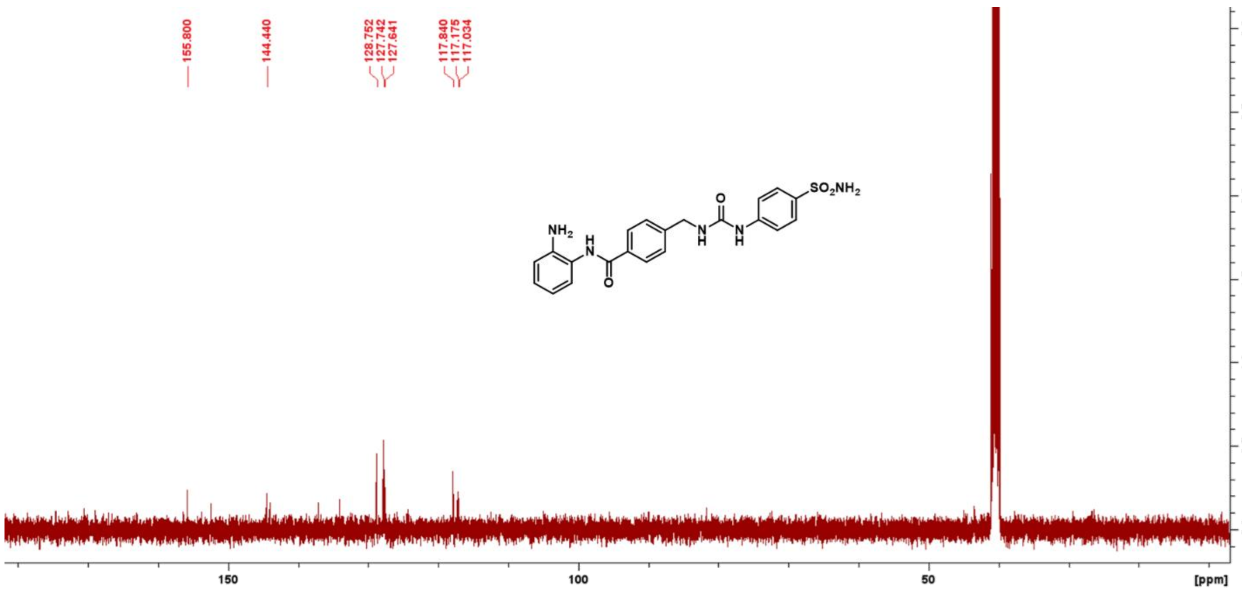

^{13}C NMR spectrum of compound **11** (400 MHz, DMSO-d$_6$)

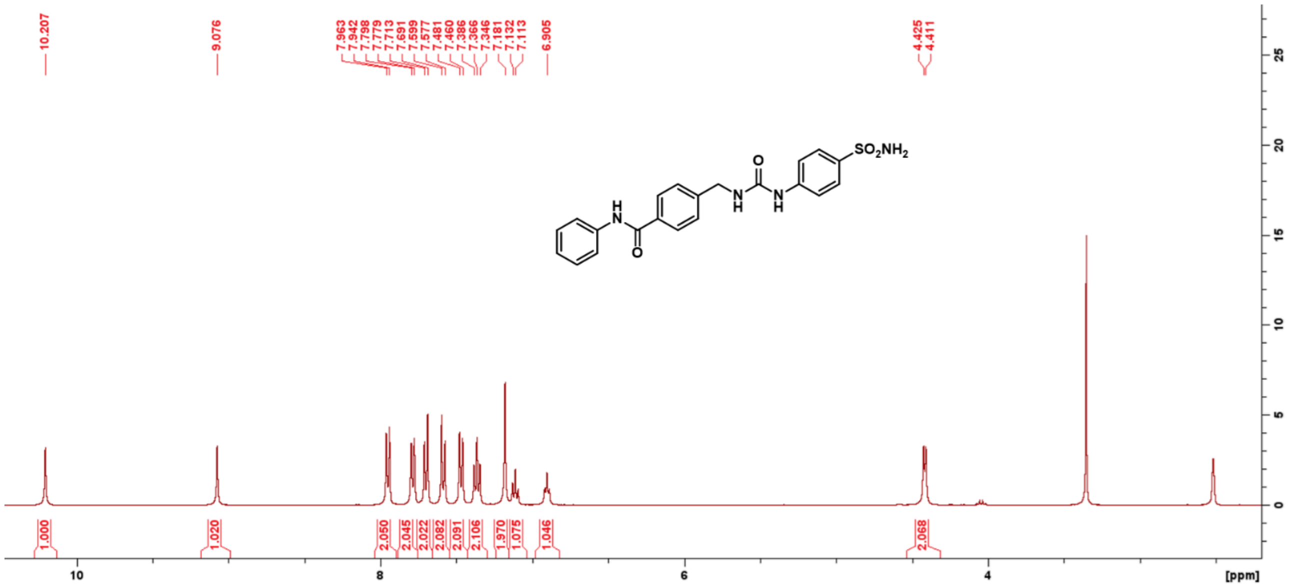

^{1}H NMR spectrum of compound **11a** (400 MHz, DMSO-d6)

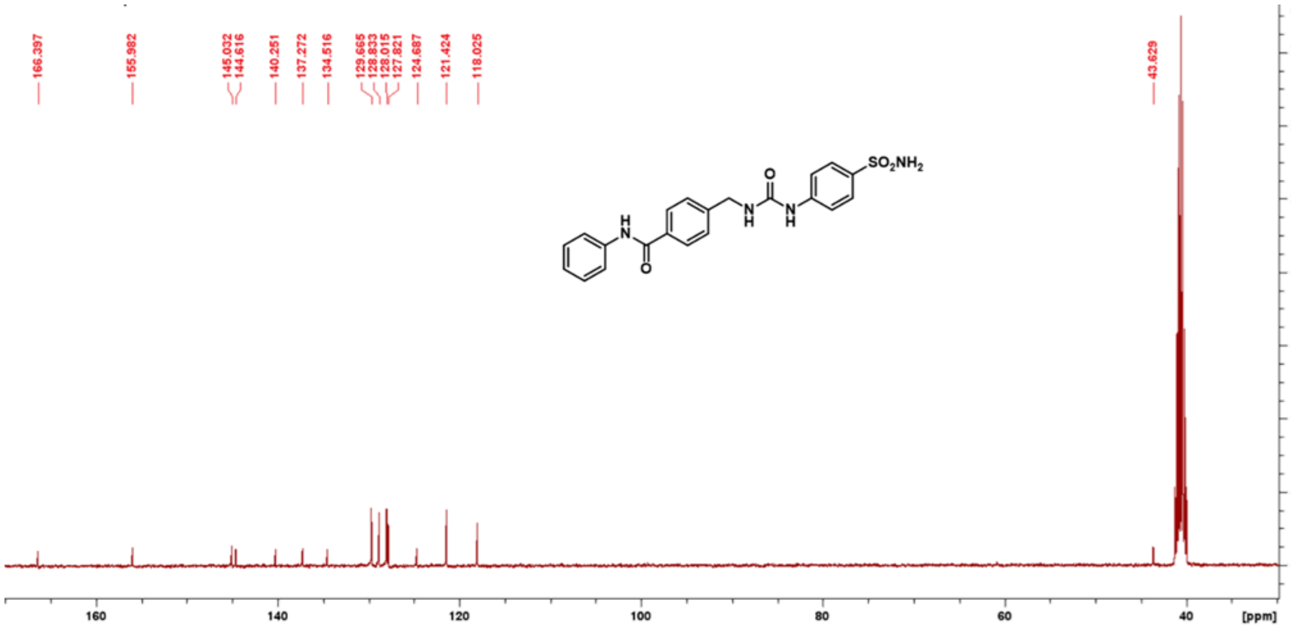

^{13}C NMR spectrum of compound **11a** (400 MHz, DMSO-d$_6$)

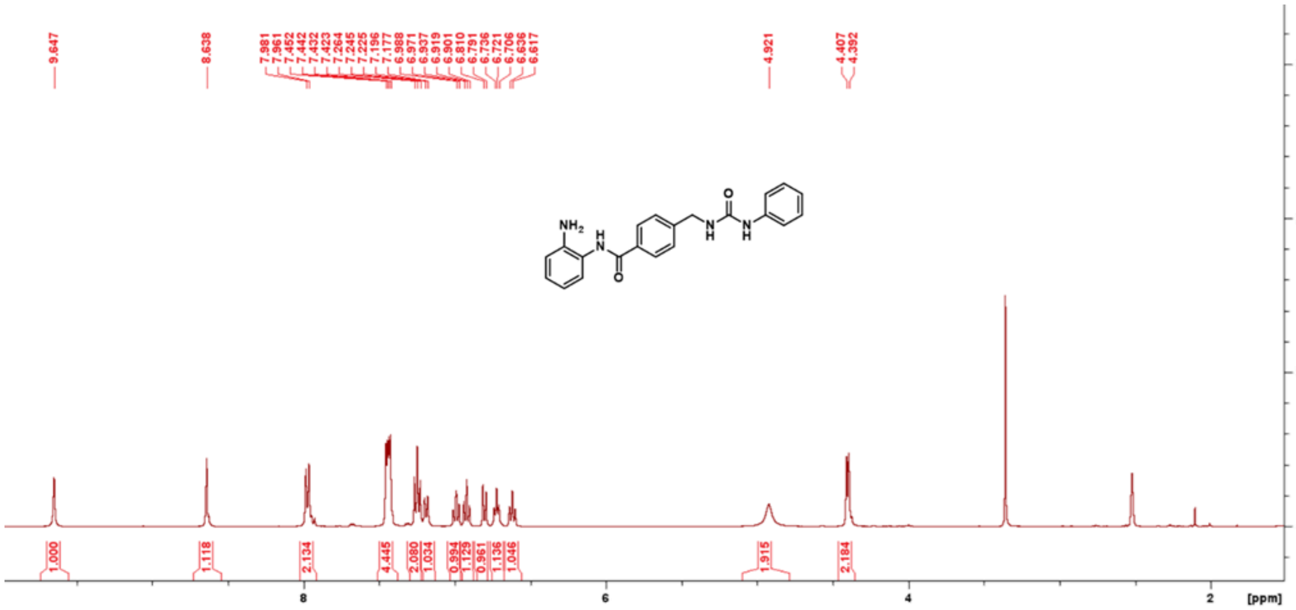

^{1}H NMR spectrum of compound **11b** (400 MHz, DMSO-d6)

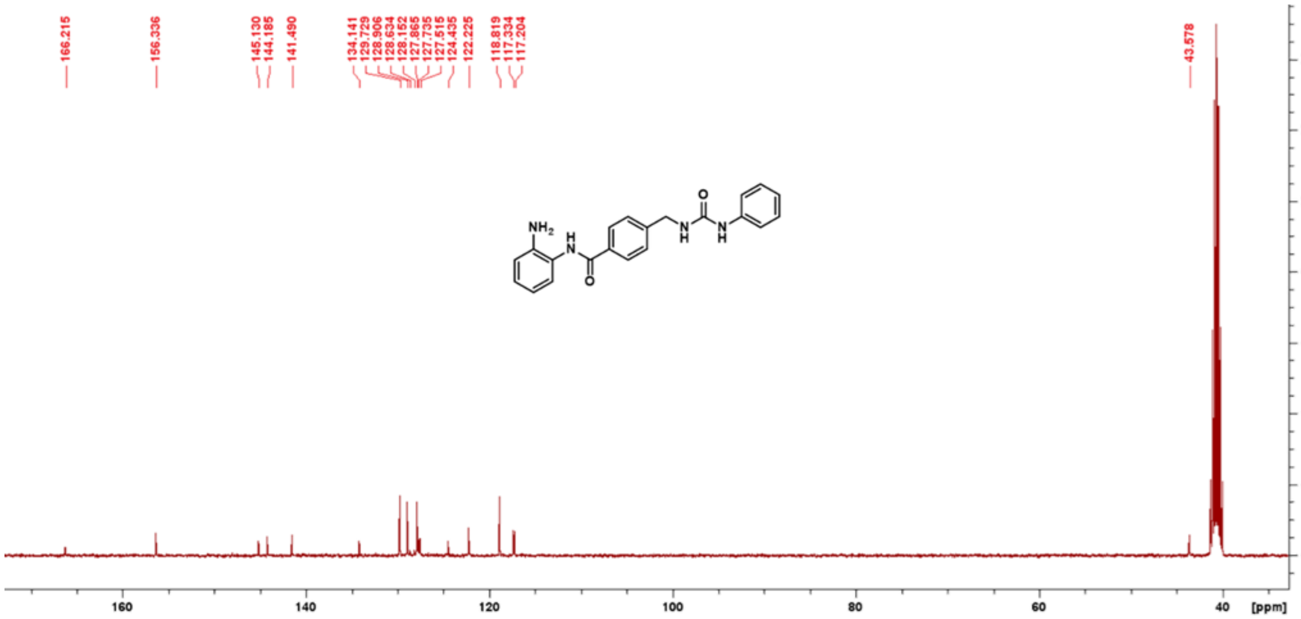

^{13}C NMR spectrum of compound **11b** (400 MHz, DMSO-d6)

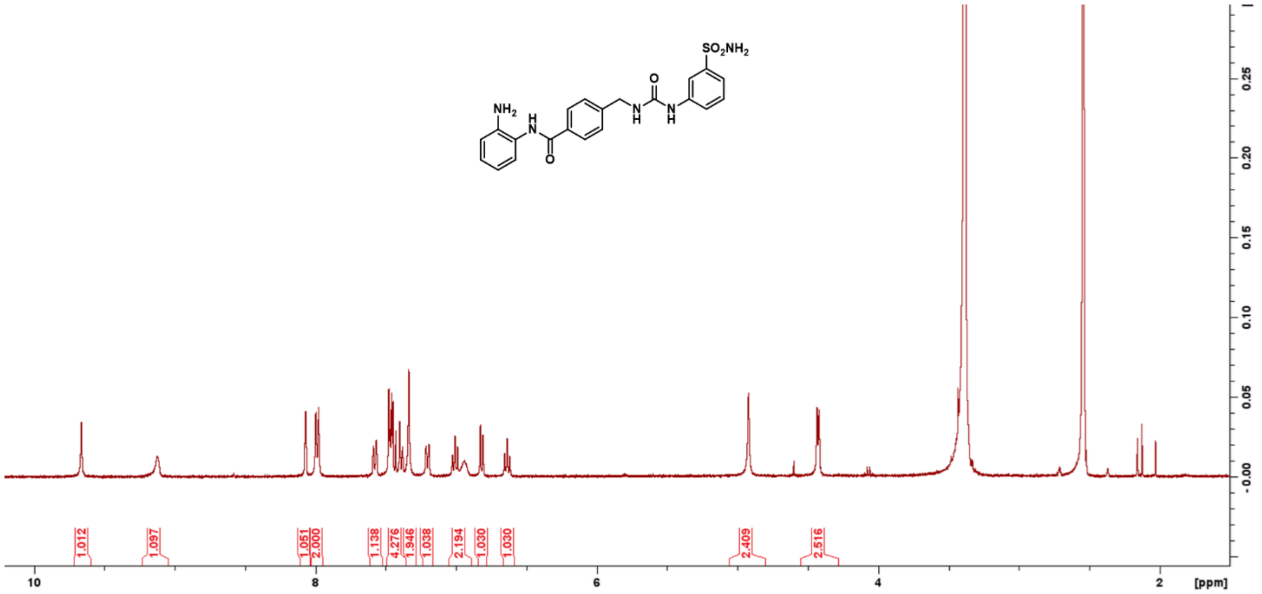

^{1}H NMR spectrum of compound **12** (400 MHz, DMSO-d$_6$)

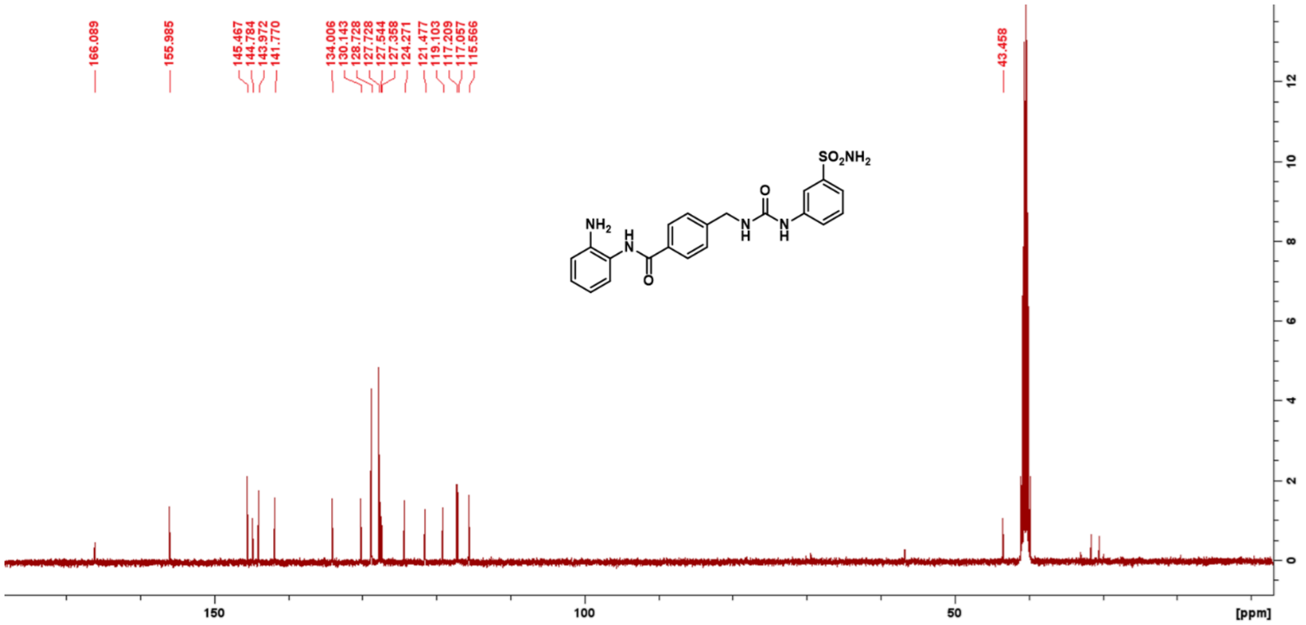

^{13}C NMR spectrum of compound **12** (400 MHz, DMSO-d$_6$)

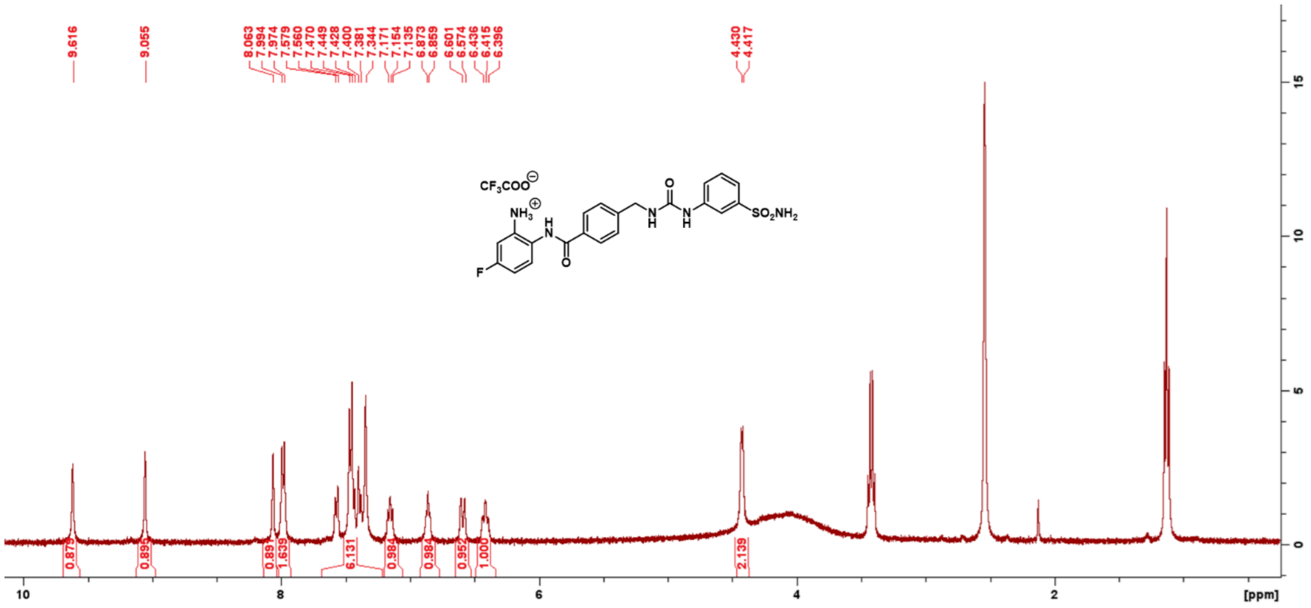

^{1}H NMR spectrum of compound **14** (400 MHz, DMSO-d6)

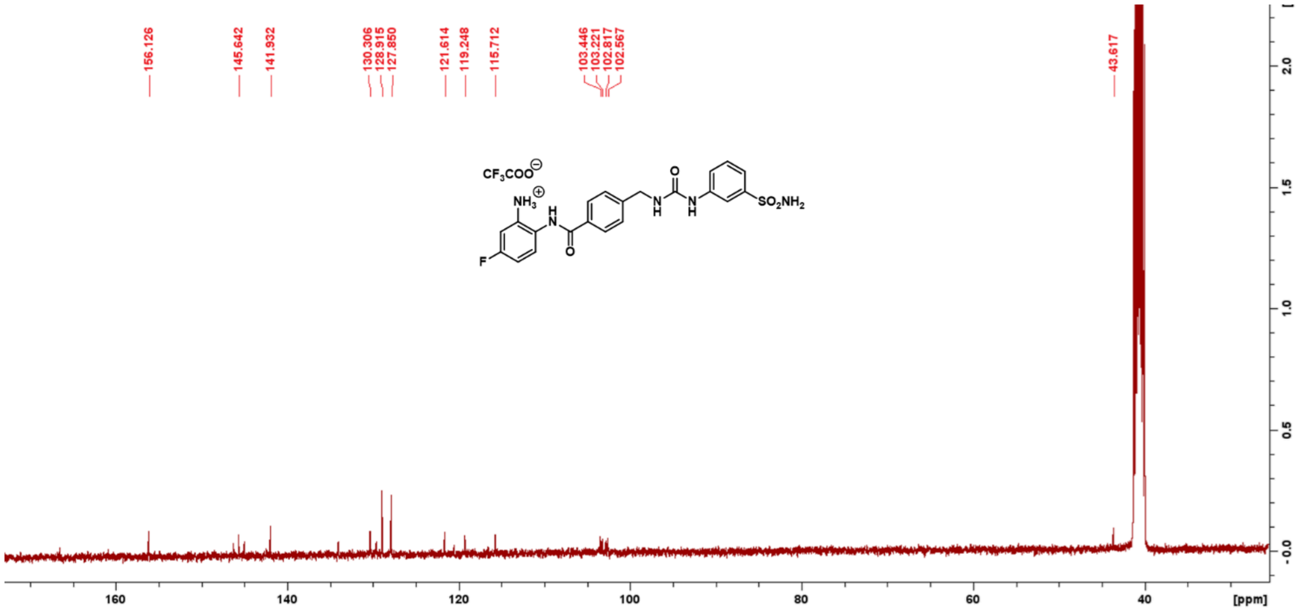

^{13}C NMR spectrum of compound **14** (400 MHz, DMSO-d6)

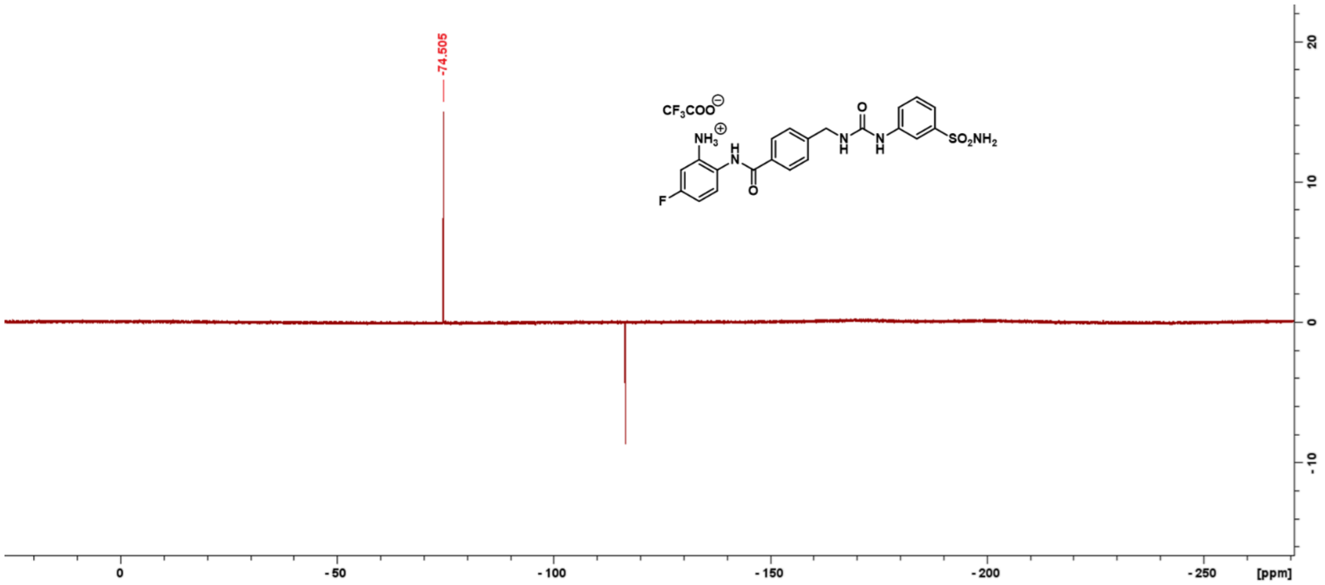

^{19}F NMR spectrum of compound **14** (400 MHz, DMSO-d_6)

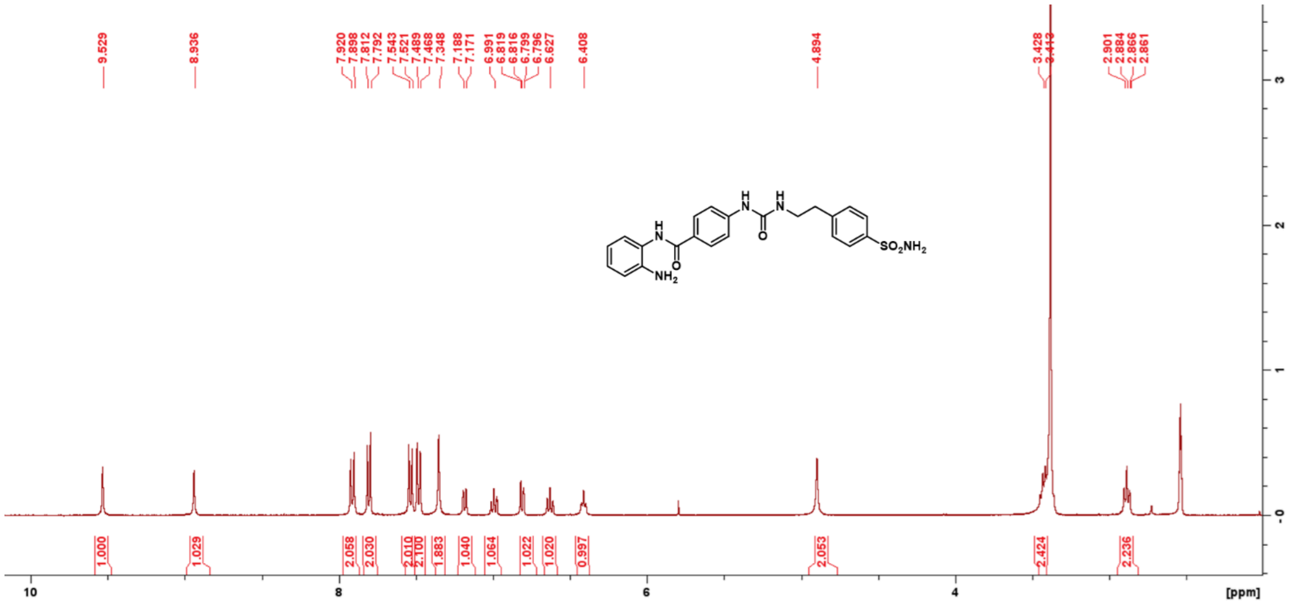

^{1}H NMR spectrum of compound **18** (400 MHz, DMSO-d_6)

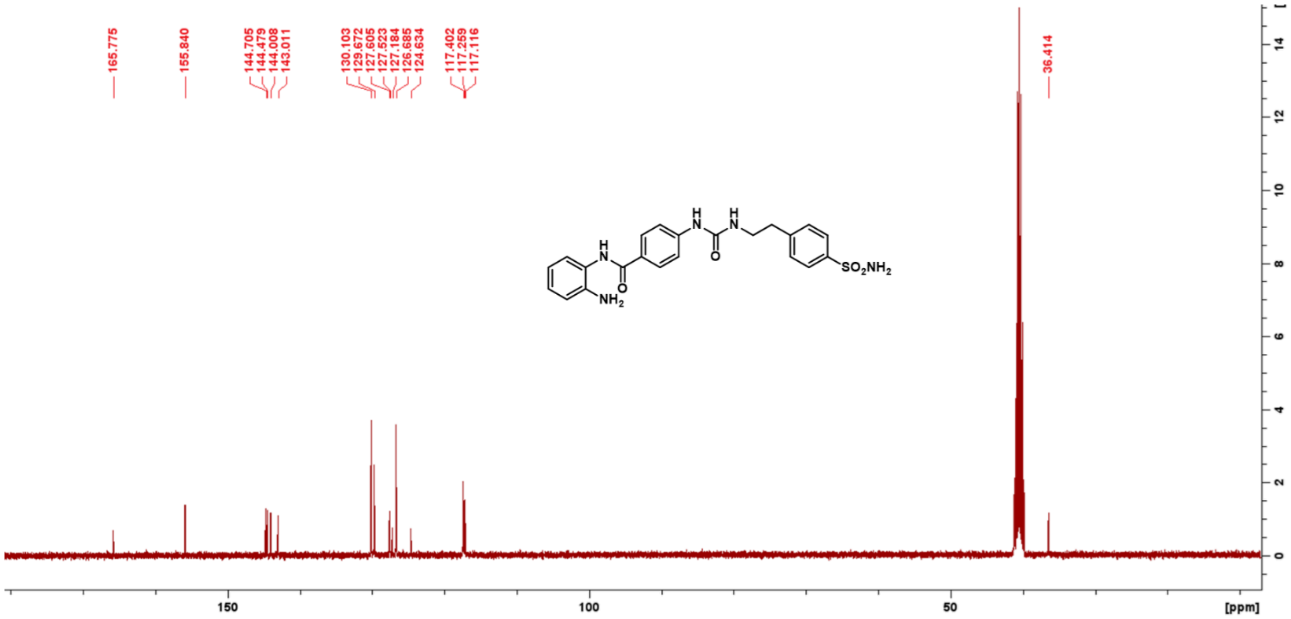

^{13}C NMR spectrum of compound **18** (400 MHz, DMSO-d_6)

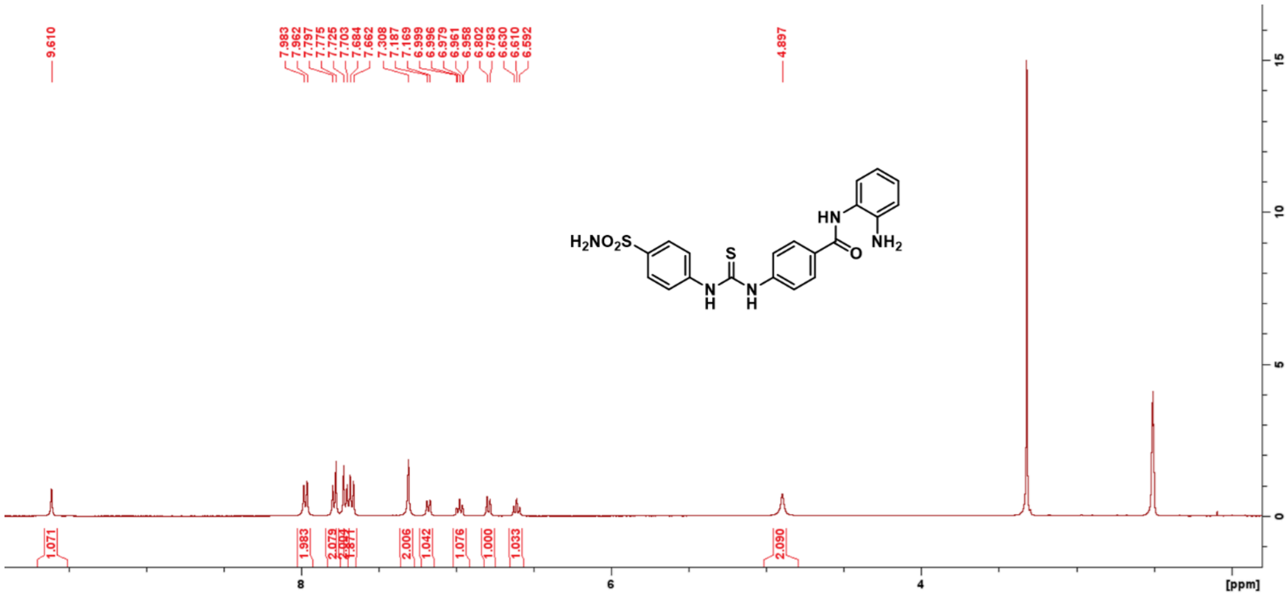

^{1}H NMR spectrum of compound **21** (400 MHz, DMSO-d6)

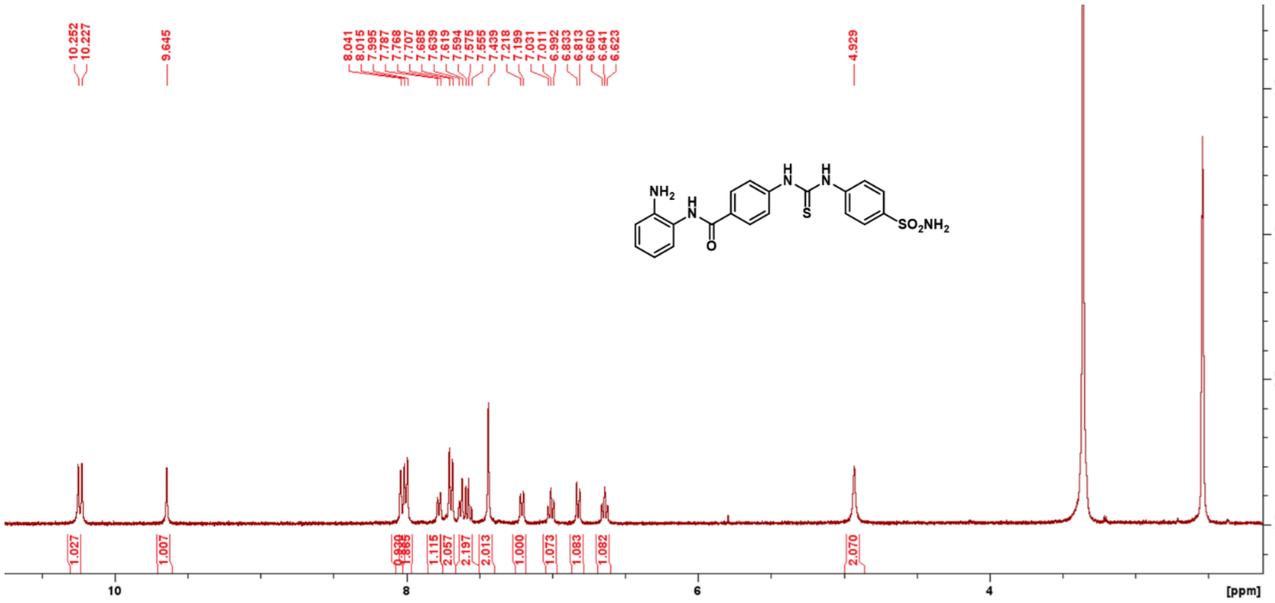

^{1}H NMR spectrum of compound **22** (400 MHz, DMSO-d$_6$)

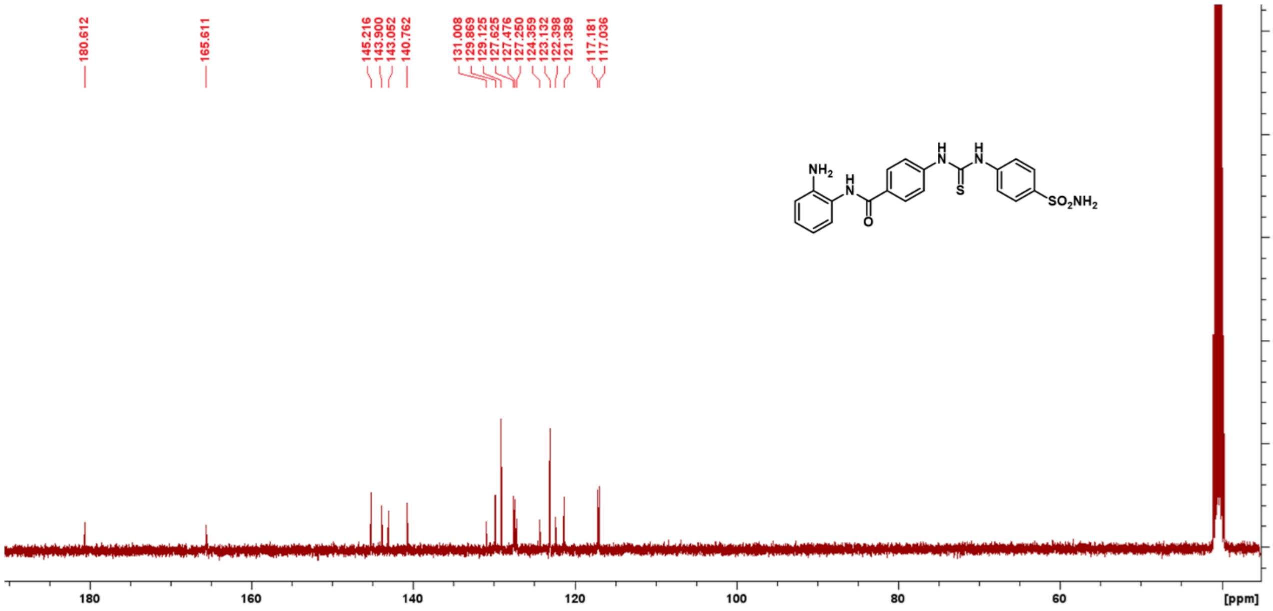

^{13}C NMR spectrum of compound **22** (400 MHz, DMSO-d$_6$)

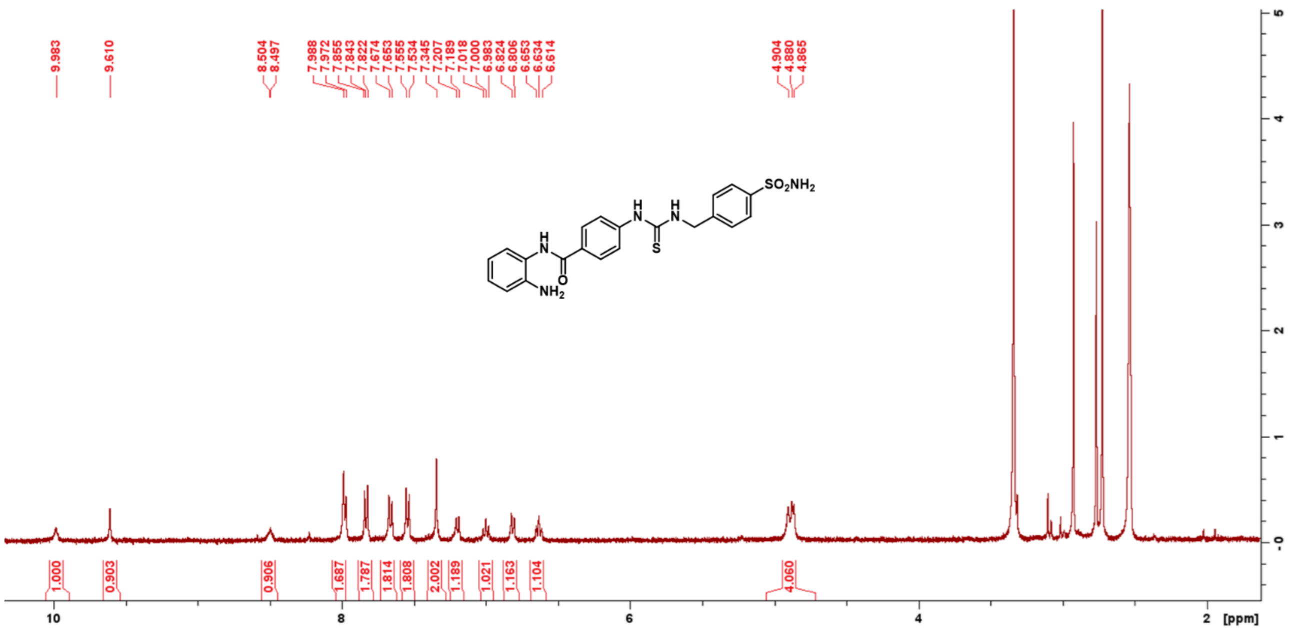

^{1}H NMR spectrum of compound **29** (400 MHz, DMSO-d$_6$)

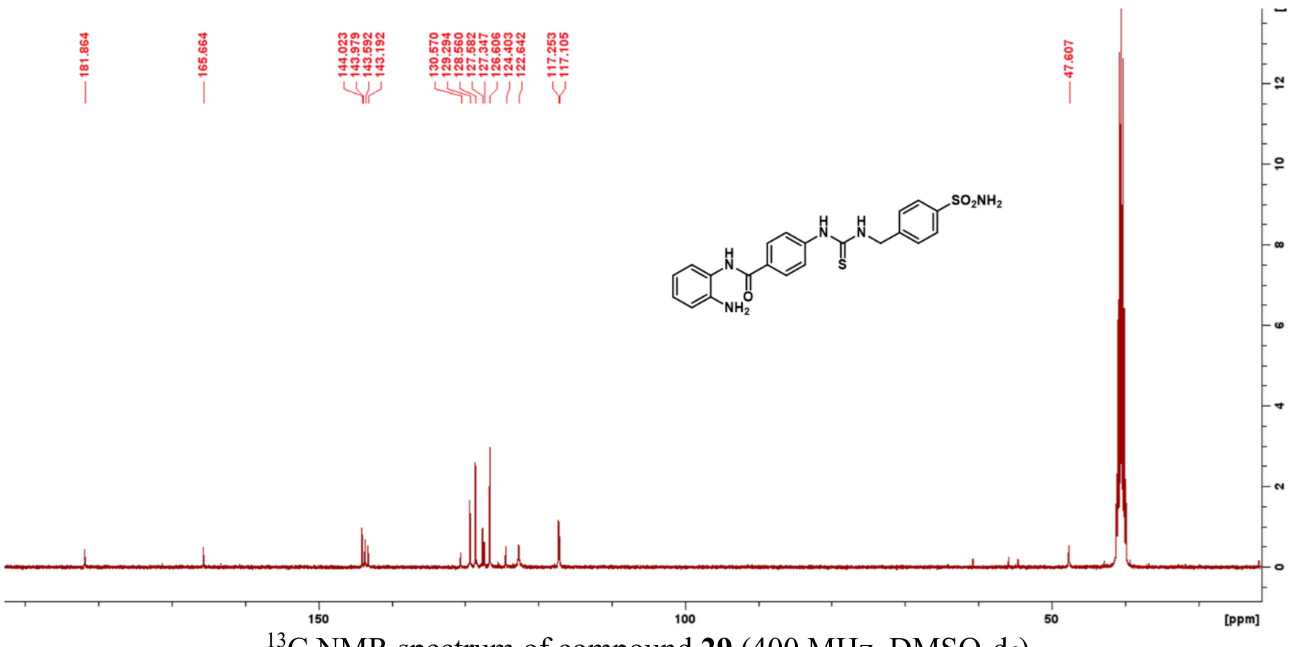

^{13}C NMR spectrum of compound **29** (400 MHz, DMSO-d$_6$)

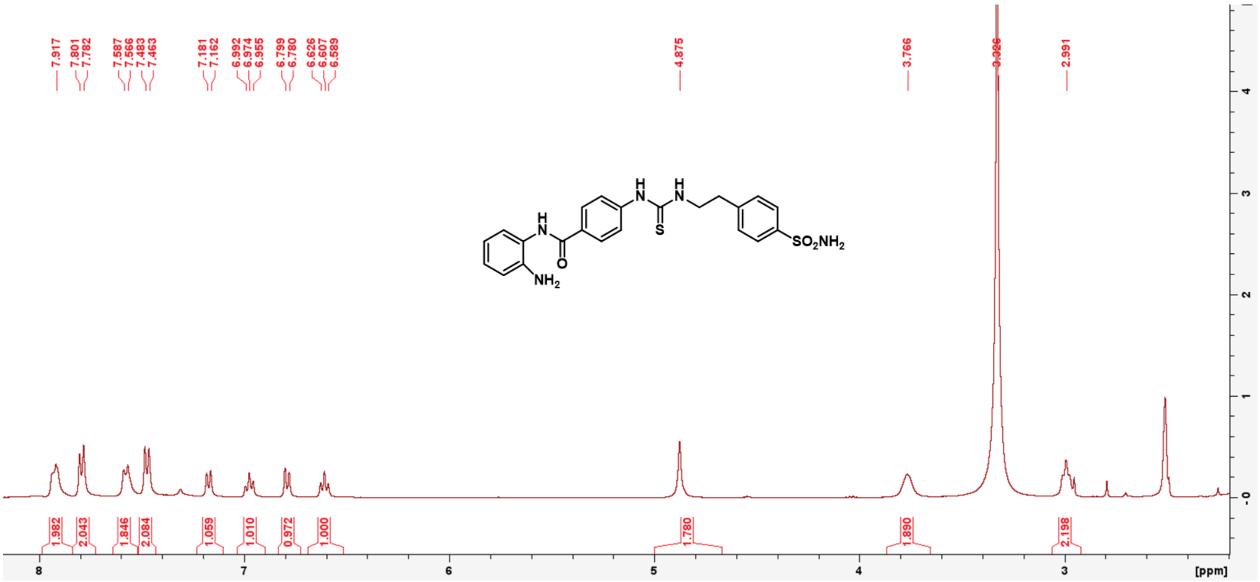

^{1}H NMR spectrum of compound **30** (400 MHz, DMSO-d6)

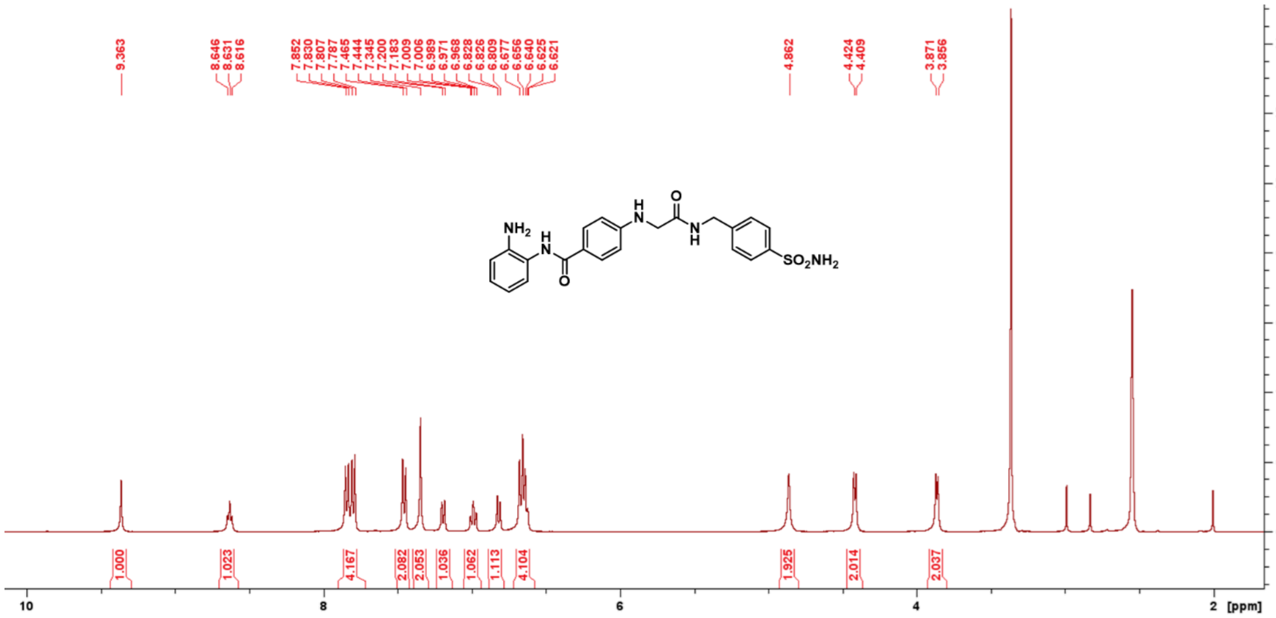

^{1}H NMR spectrum of compound **37** (400 MHz, DMSO-d$_6$)

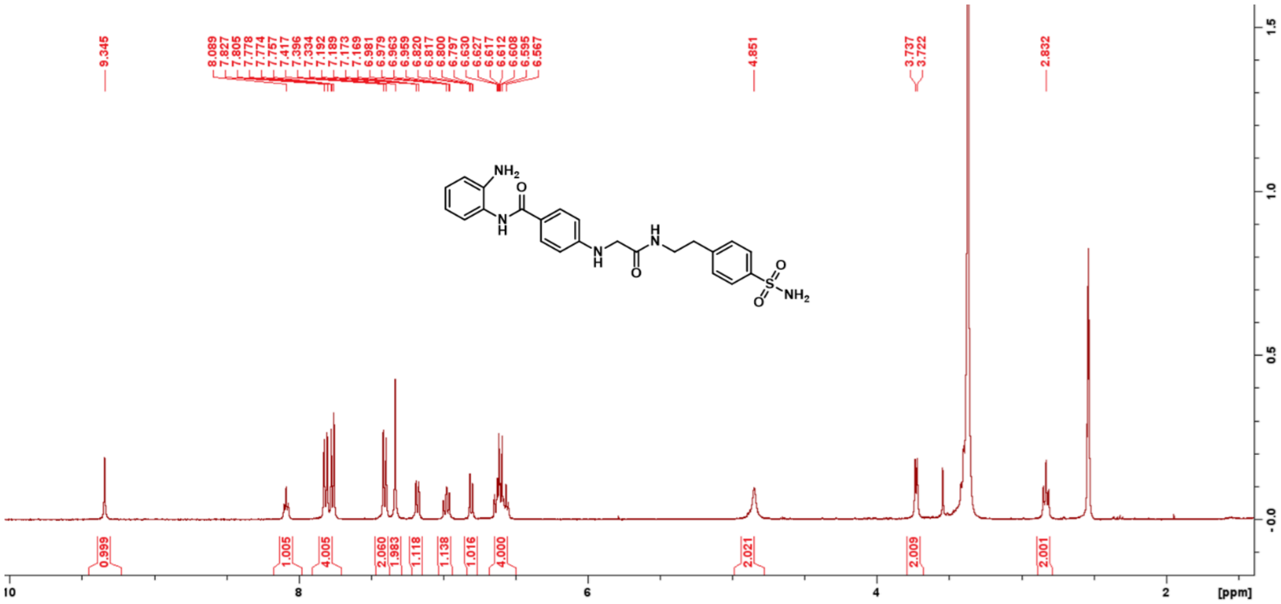

^{1}H NMR spectrum of compound **38** (400 MHz, DMSO-d6)

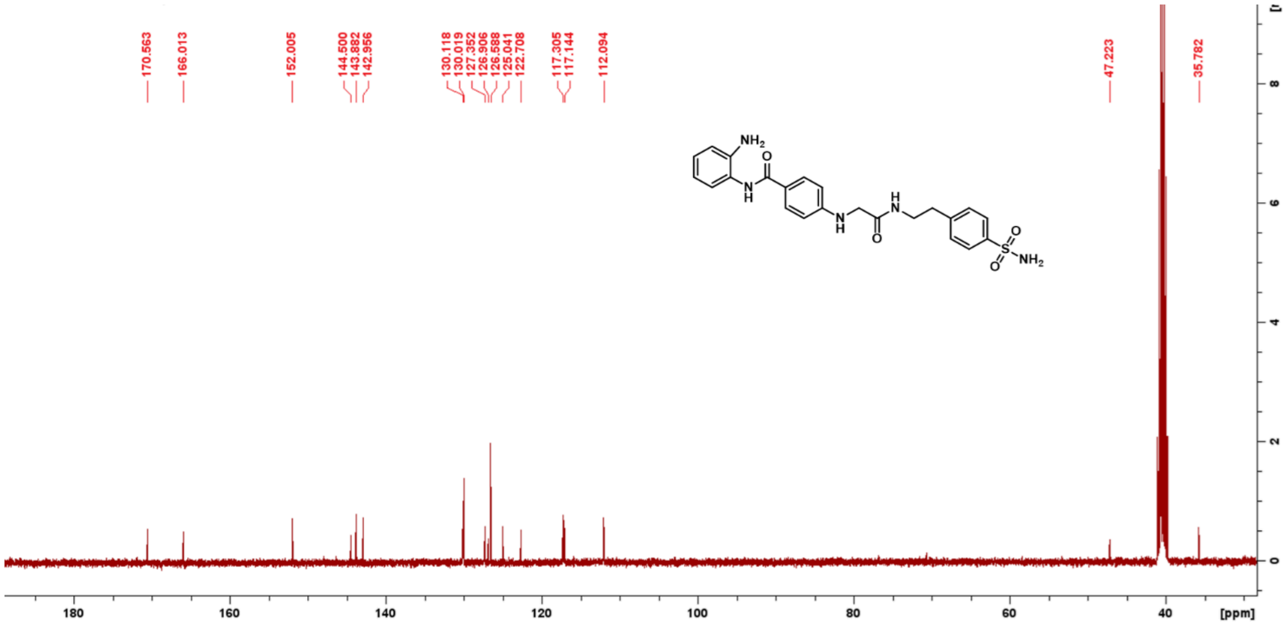

^{13}C NMR spectrum of compound **38** (400 MHz, DMSO-d6)

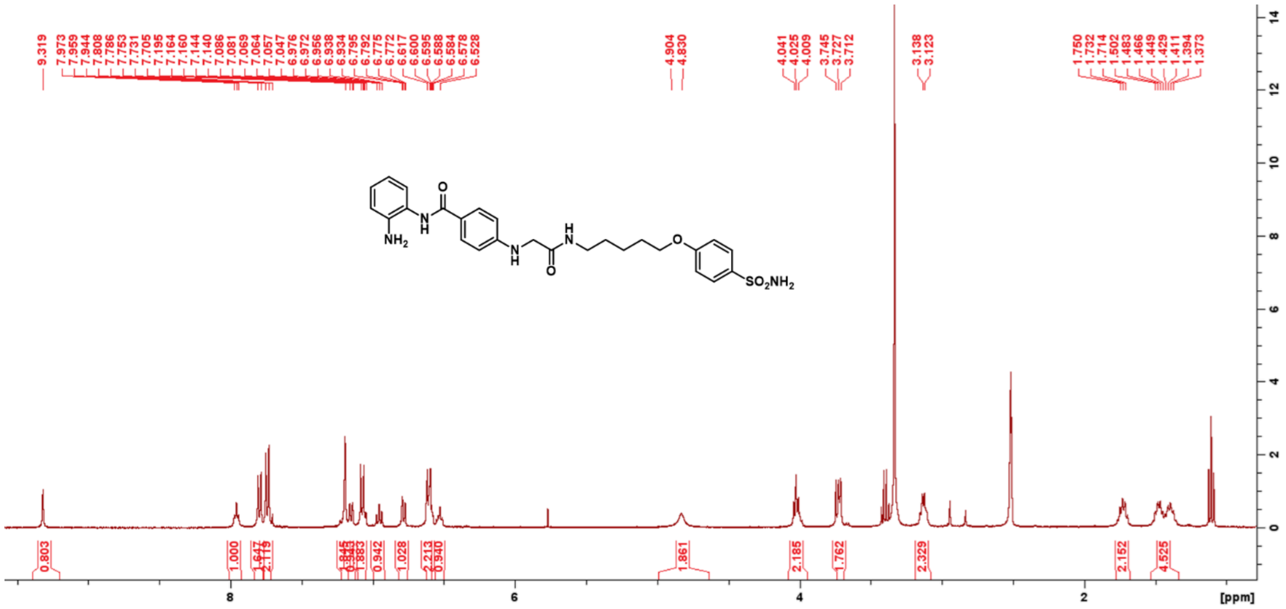

^{1}H NMR spectrum of compound **39** (400 MHz, DMSO-d6)

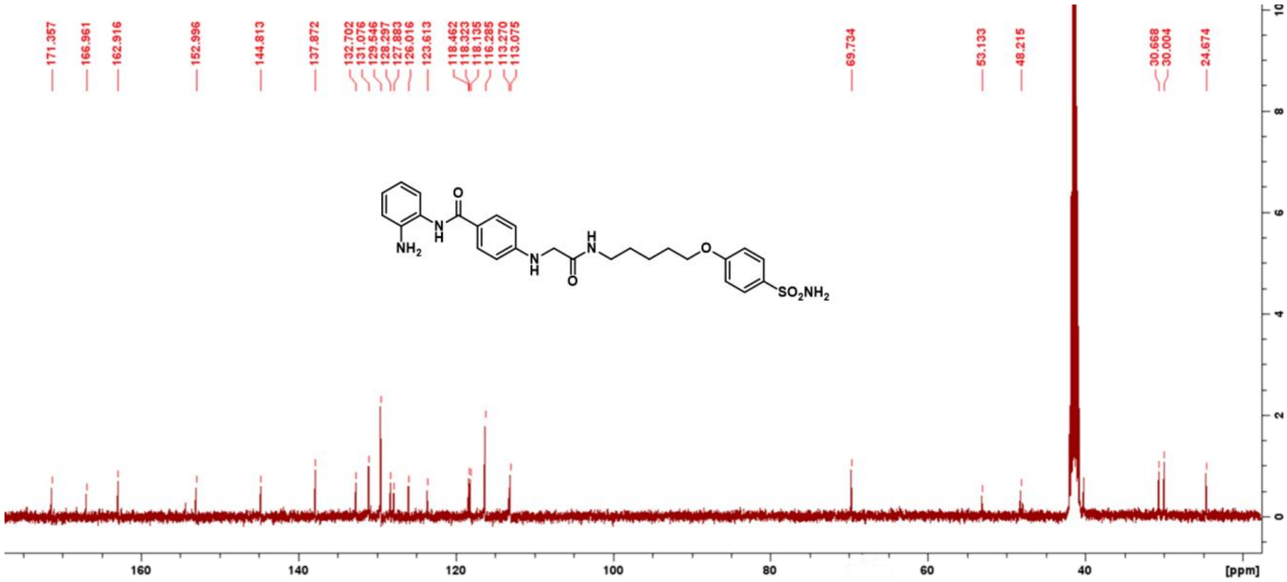

^{13}C NMR spectrum of compound **39** (400 MHz, DMSO-d6)

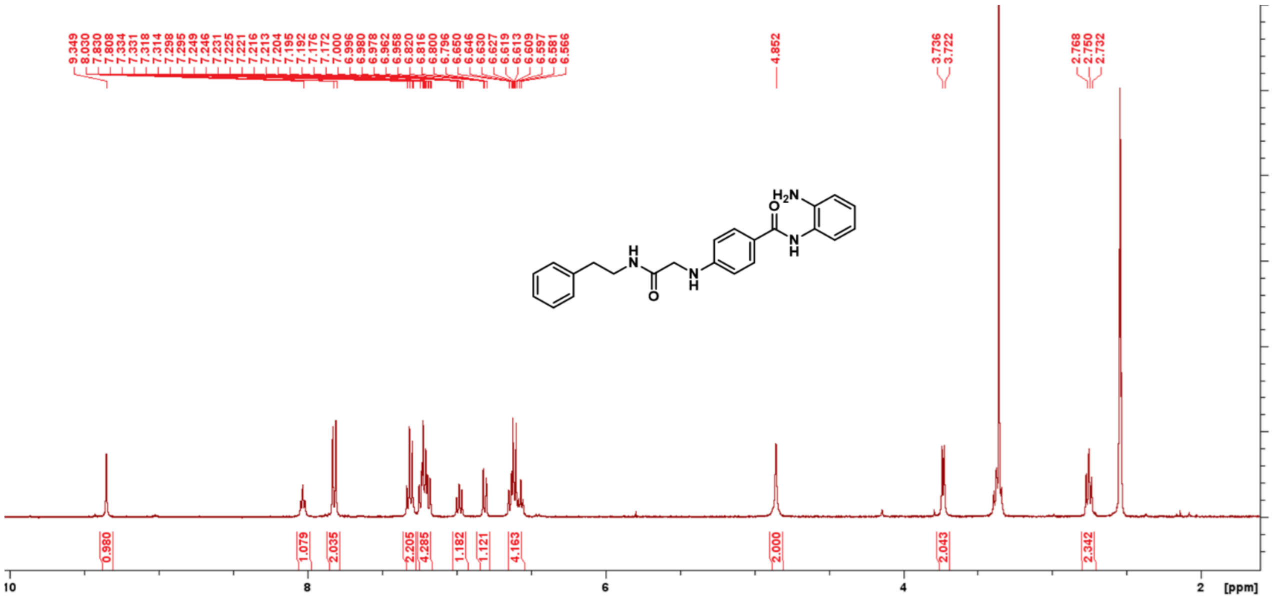

^{1}H NMR spectrum of compound **40** (400 MHz, DMSO-d$_6$)

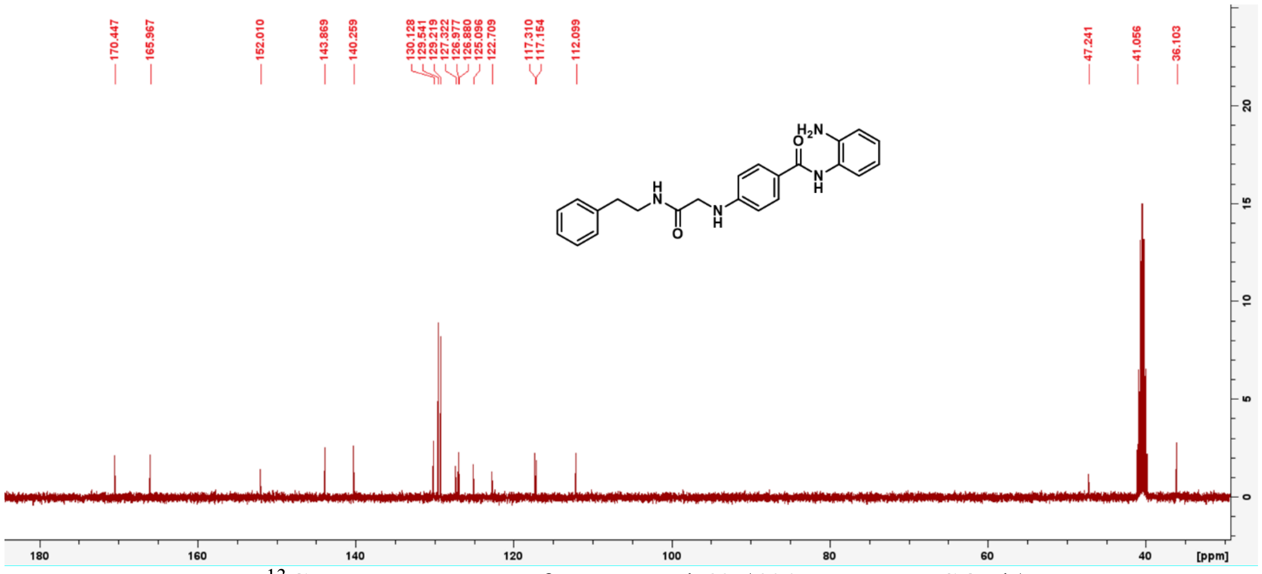

^{13}C NMR spectrum of compound **40** (400 MHz, DMSO-d$_6$)

Table S1: Summary of Data Collection and Atomic Model Refinement Statistics for hCA II

	**hCA II + 38**	**hCA II + 37**
PDB ID	7QRK	7QSE
Wavelength (Å)	1.0000	1.0000
Space Group	P21	P21
Unit cell (a, b, c, α, β, γ) (Å,°)	42.27, 41.23, 72.06, 89.99, 104.31, 90.04	42.32, 41.24, 72.08, 90.00, 104.35, 90.00
Limiting resolution (Å)	41.23-1.43 (1.45-1.43)	41.24-1.43 (1.45-1.43)
Unique reflections	44771 (2154)	43568 (2006)
Rmerge (%)	4.0 (41.0)	2.9 (26.1)
Rmeas (%)	4.8 (50.7)	3.5 (32.1)
Redundancy	6.1 (5.2)	6.4 (5.6)
Completeness overall (%)	99.8 (98.2)	97.2 (92.4)
<I/σ(I)>	21.5 (3.2)	32.9 (5.7)
CC (1/2)	99.9 (98.2)	100 (93.0)
**Refinement statistics**		
Resolution range (Å)	41.23-1.43	41.24-1.43
Rfactor (%)	15.15	14.70
Rfree(%)	16.96	16.42
r.m.s.d. bonds(Å)	0.0141	0.0147
r.m.s.d. angles (°)	1.9058	1.9990
**Ramachandran statistics** (%)		
Most favored	97.3	97.3
additionally allowed	2.7	2.7
outlier regions	0.0	0.0
**Average B factor (Å^2)**		
All atoms	15.339	14.679
inhibitors	41.735	30.551
solvent	25.708	25.546

Figure S1. Electron density of inhibitors **38** (**A**) and **37** (**B**) bound to zinc (grey) in hCA II active site. $2F_o$-F_c maps and contoured to the 1.0 σ level.

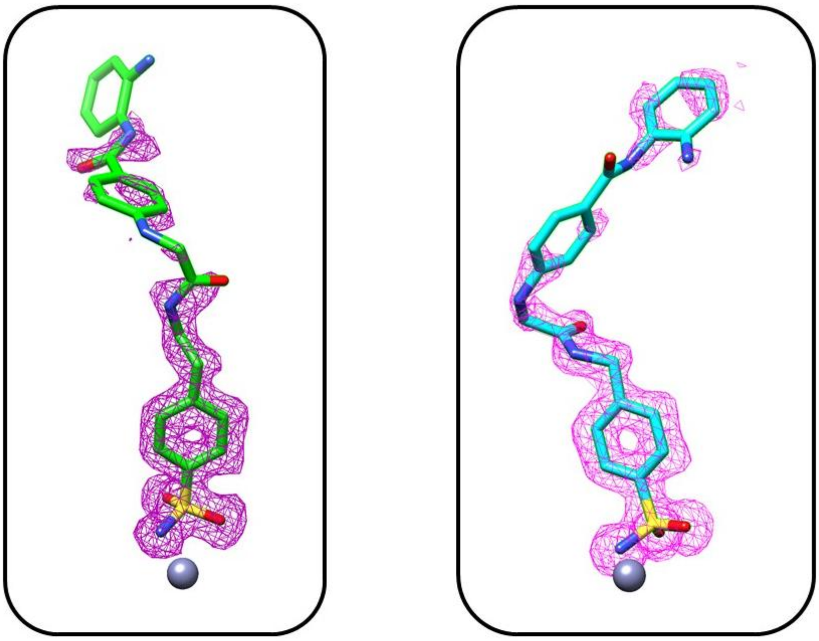

A B

Figure S2. SAHA and TSA bound conformations in different HDACs. The PDB codes are given and associated by color to each ligand conformation

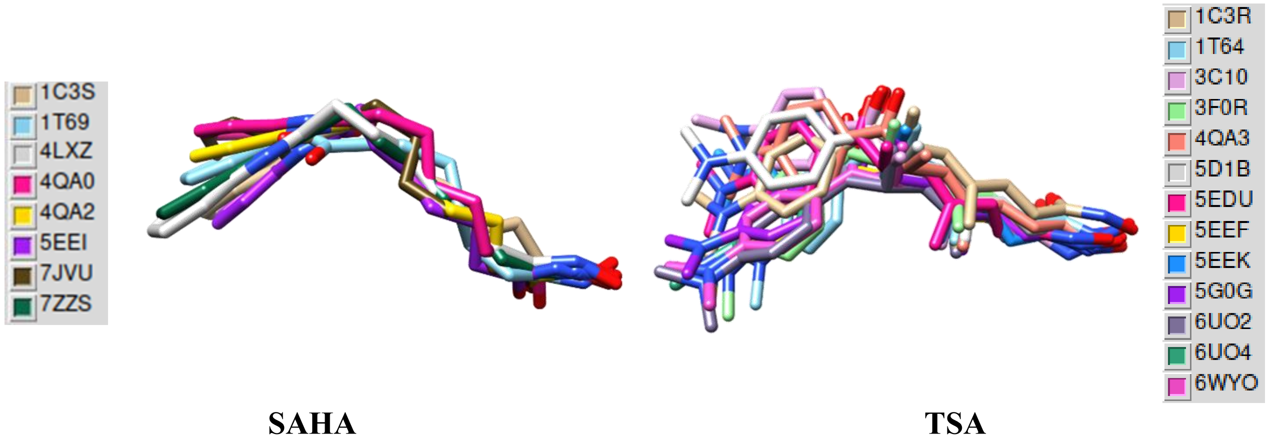

Table S2.

Program[#]	Scoring Functions/Setting/Force Field[*]	ECRD[†]	RCRD[‡]
glamdock [7]	glamdock_new_energy	1.07	0.75
plants [8]	plp95	0.95	1.02
ledock [9]	ledock	0.95	1.07
glamdock	glamdock	1.29	0.87
glamdock	glamdock_screening	1.36	0.89
Plants	chemplp	1.01	1.25
glamdock	glamdock_fast_screening	1.51	0.84
igemdock [10]	igemdock	0.42	1.97
smina [11]	ad4_scoring	1.42	1.06
psovina [12]	psovina	0.64	1.88
Glamdock	glamdock_hq_old_energy	1.53	1.00
Plants	plp	0.94	1.63
qvina2 [13]	qvina2	1.02	1.57
Smina	vinardo	1.37	1.54
Glamdock	glamdock_hq_new_energy	1.66	1.32
vina [14]	vina	1.48	1.56
Smina	vina	1.50	1.55
vinaSH [15]	vinaSH	1.49	1.57
vinaXB [16]	vinaXB	1.51	1.55
qvinaw [17]	qvinaw	1.51	1.56
Glamdock	glamdock_quaternion	1.49	1.60
imolsdock [18]	GAFF	0.48	2.61
mpsovina [19]	mpsovina	1.65	1.71

lmolsdock	MMFF94	0.48	4.29
Vina	vinardo	9.22	1.24
idock [20]	idock	9.20	1.83
betadock [21]	betadock	8.17	16.49

Molecular docking program name

* Scoring Function or Force Field or particular ready setting for a given program.

† Experimental Conformation Re-Docking: RMSD values obtained using as starting conformation the ligand extracted from the experimental PDB

‡ Random Conformation Re-Docking: RMSD values obtained using as starting conformation a random generated conformation of the ligand

Table S3. Inhibition data of hCA I, II, IV and IX with compound **40** and the standard sulfonamide inhibitor acetazolamide (**AAZ**) by a stopped flow CO_2 hydrase assay. [22]

**Compound**	K_I **(nM)***			
	**hCA I**	**hCA II**	**hCA IX**	**hCA XII**
**40**	>10000	>10000	>10000	>10000
**AAZ**	250.0	12.1	25.6	5.7

* Mean from 3 different assays, by a stopped-flow technique (errors were in the range of ± 5-10 % of the reported values).

Table S4. Inhibition data of HDACs 1, 3, 4, 6 and 8 with compound **40**.

**Compound**	IC_{50} (μM)^{a,b,c}				
	**HDAC1**	**HDAC3**	**HDAC4**	**HDAC6**	**HDAC8**
**40**	0.167	0.677	ND	ND	6.07
**TRC A**	0.018	0.024	ND	0.004	0.619
**TMP 269**	ND	ND	0.239	ND	ND

^{a}ND= no inhibition at 100 μM, ^{b} compounds tested in singlet 10-dose mode with 3-fold serial dilution starting from a 200 μM solution.^{c} Mean from 3 different assays

Figure S3. Flow cytometry analysis of hCA IX basal expression in HCT-8 and HCT-116 colon cancer, MDA-MB-231 and BT-474 breast cancer, A375, 501Mel, and Sk-Mel-28 melanoma cell lines.

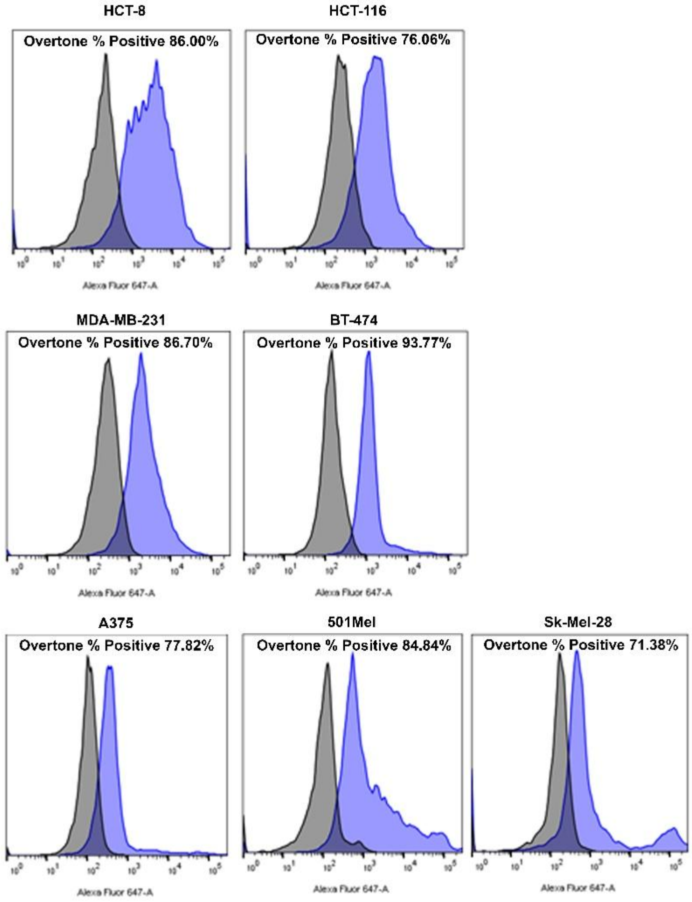

Figure S4. IC_{50} values of **11** on colon carcinoma cell lines (**A**), mammary carcinoma cell lines (**B**), and melanoma cell lines (**C**).

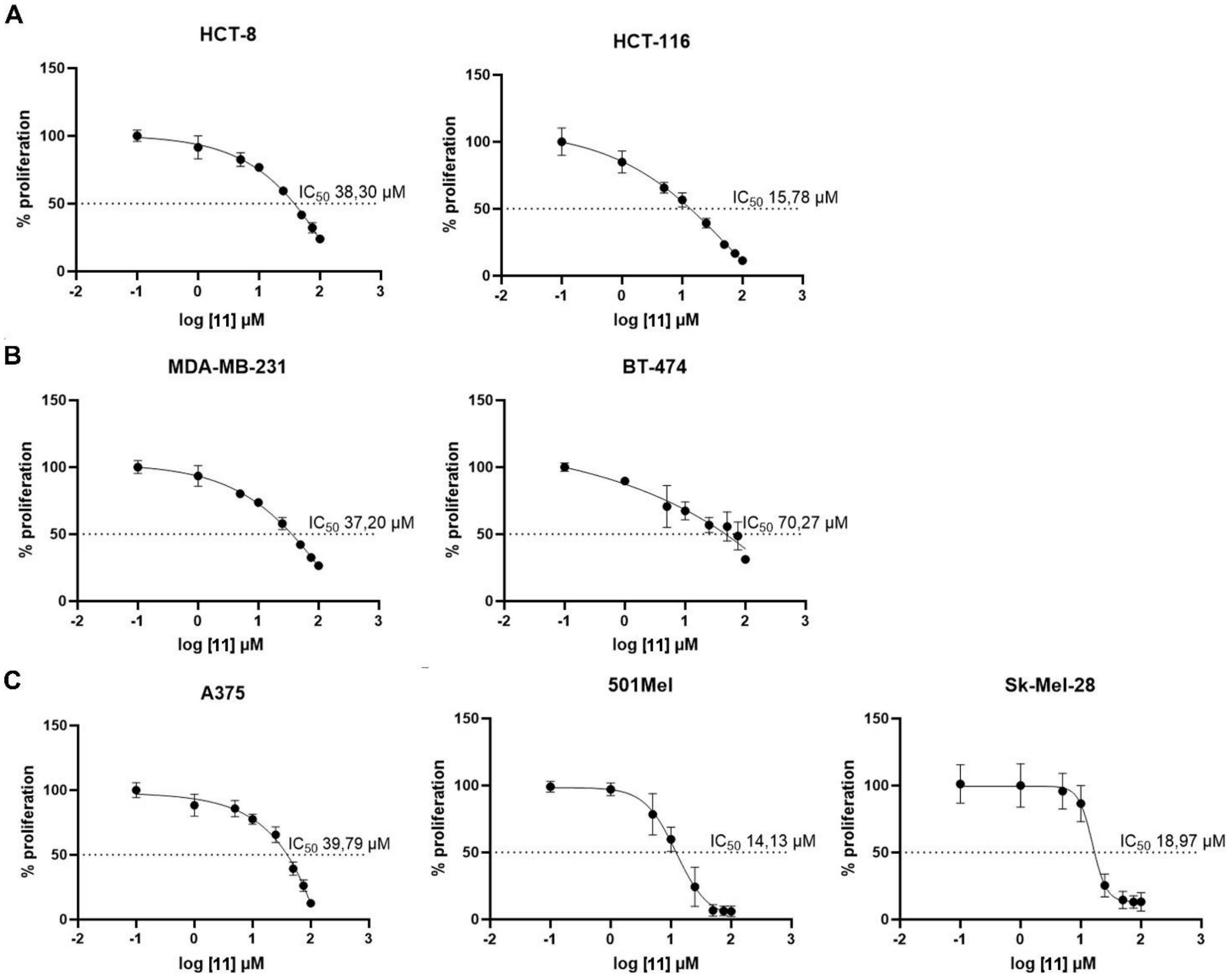

Figure S5. IC_{50} values of **14** on colon carcinoma cell lines (**A**), mammary carcinoma cell lines (**B**), and melanoma cell lines (**C**).

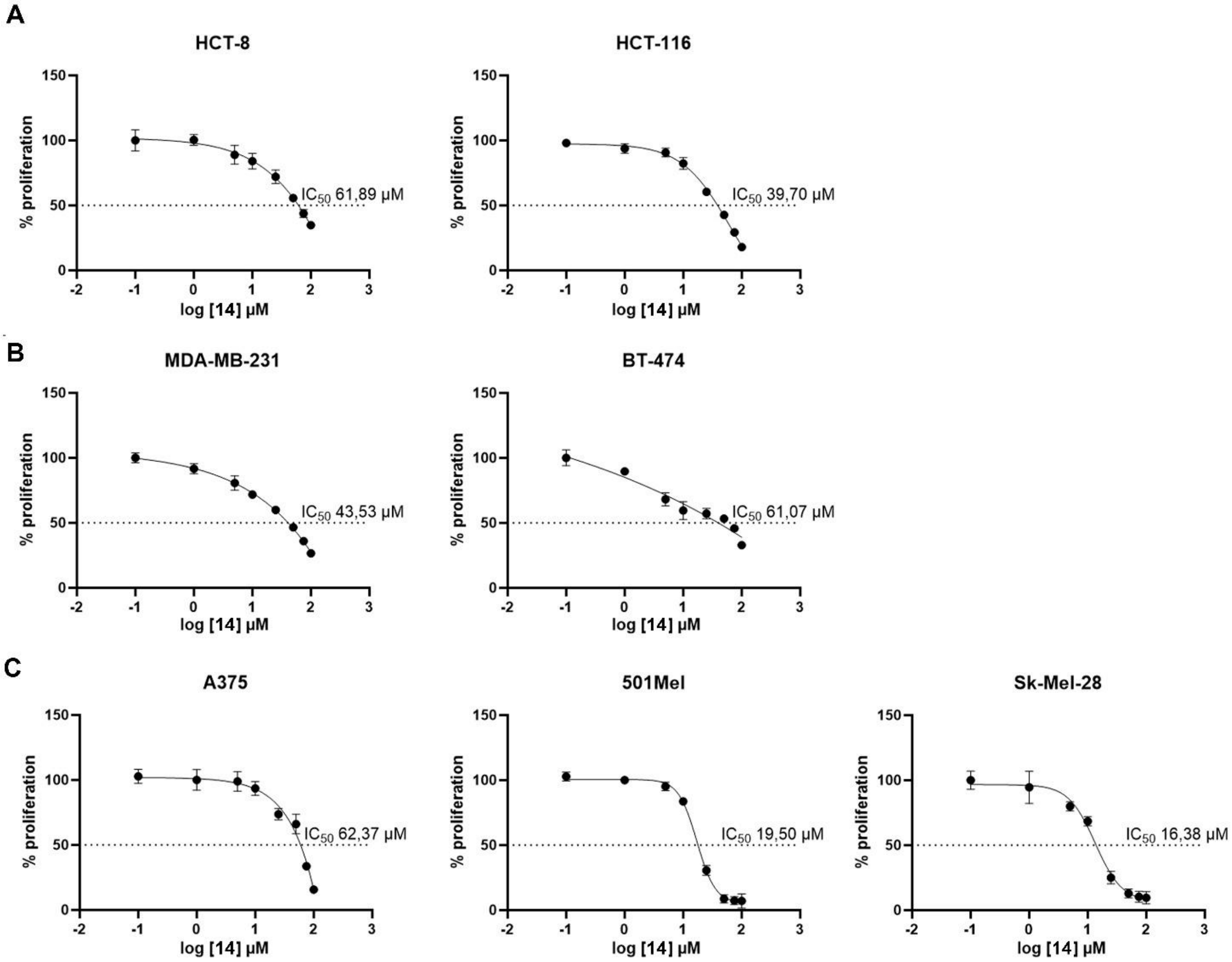

Figure S6. Representative plots with relative IC_{50} values of SAHA in HCT-8 and HCT-116 colon carcinoma cell lines, MDA-MB-231 and BT-474 mammary carcinoma cell lines, A375, 501Mel, and Sk-Mel-28 melanoma cell lines, and normal endothelial colony-forming cells (ECFC).

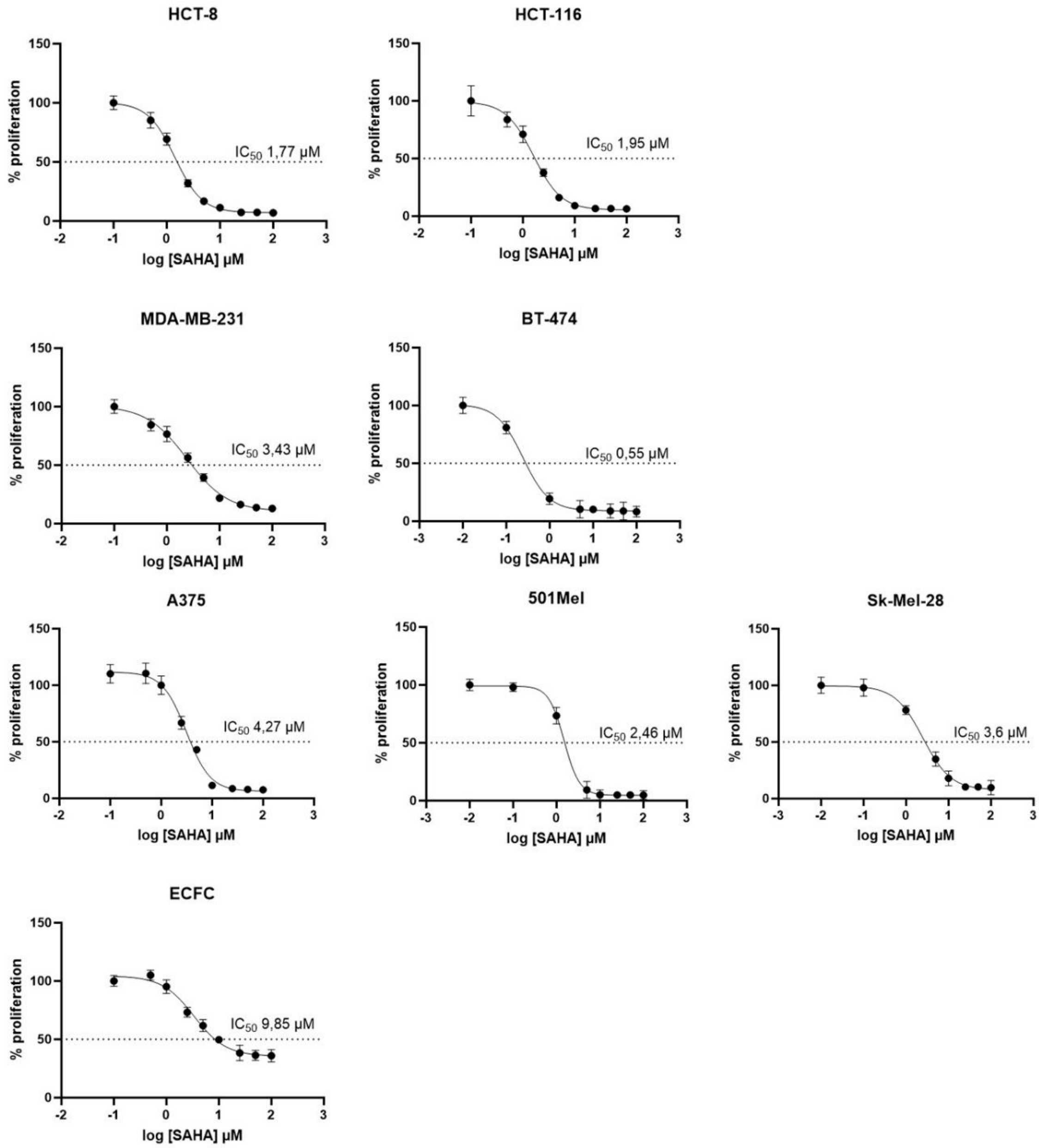

Figure S7. Representative dot plots (FL1-H vs FL3-H) of HCT116 cells treated for 72h with compound **11** at 5, 10 and 25 µM. Quadrants indicate different cell populations based on Annexin V/PI staining: viable cells (--; Q3), early apoptotic (+-,Q4), late apoptotic or necrotic (++, Q2) and necrotic (-+, Q1). Experiments were performed in triplicate ± SD (standard deviation). p-values were obtained using one-way ANOVA *p<0.05, *vs* control (CTR), GraphPad Prism 10.1.

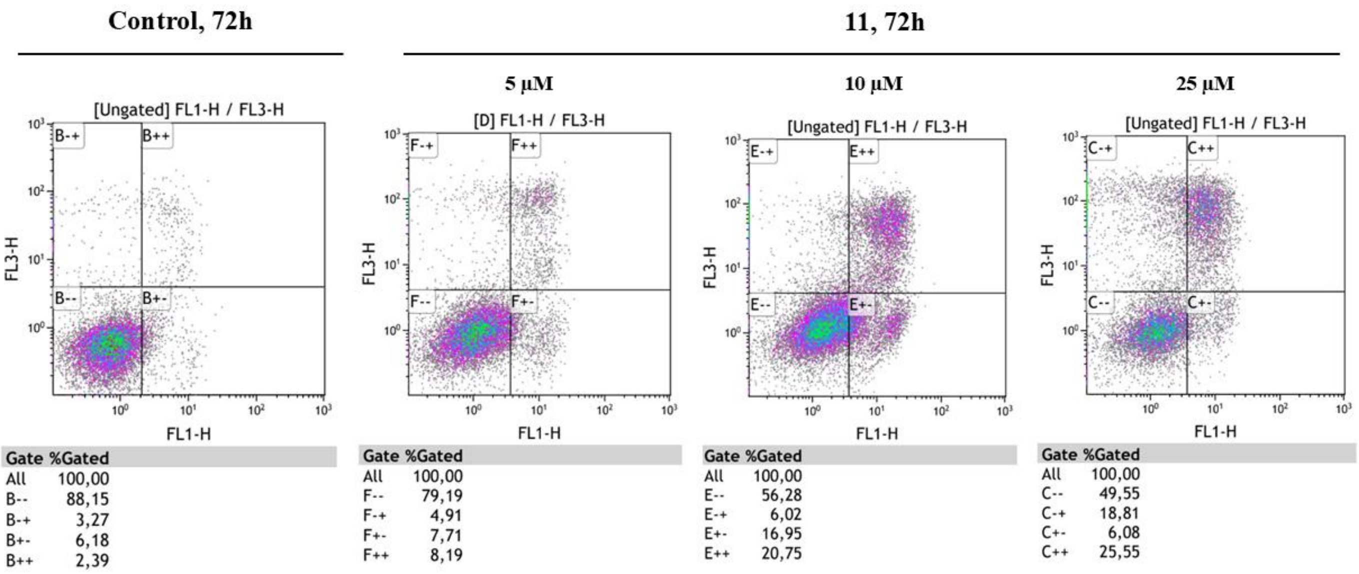

Figure S8. Representative dot plots (FL1-H vs FL3-H) of HCT-116 cells treated for 72h with compound **14** at 5, 10 and 25 µM. Quadrants indicate different cell populations based on Annexin V/PI staining: viable cells (--; Q3), early apoptotic (+-,Q4), late apoptotic or necrotic (++, Q2) and necrotic (-+, Q1). Experiments were performed in triplicate ± SD (standard deviation). p-values were obtained using one-way ANOVA *p<0.05, *vs* control (CTR), GraphPad Prism 10.1.

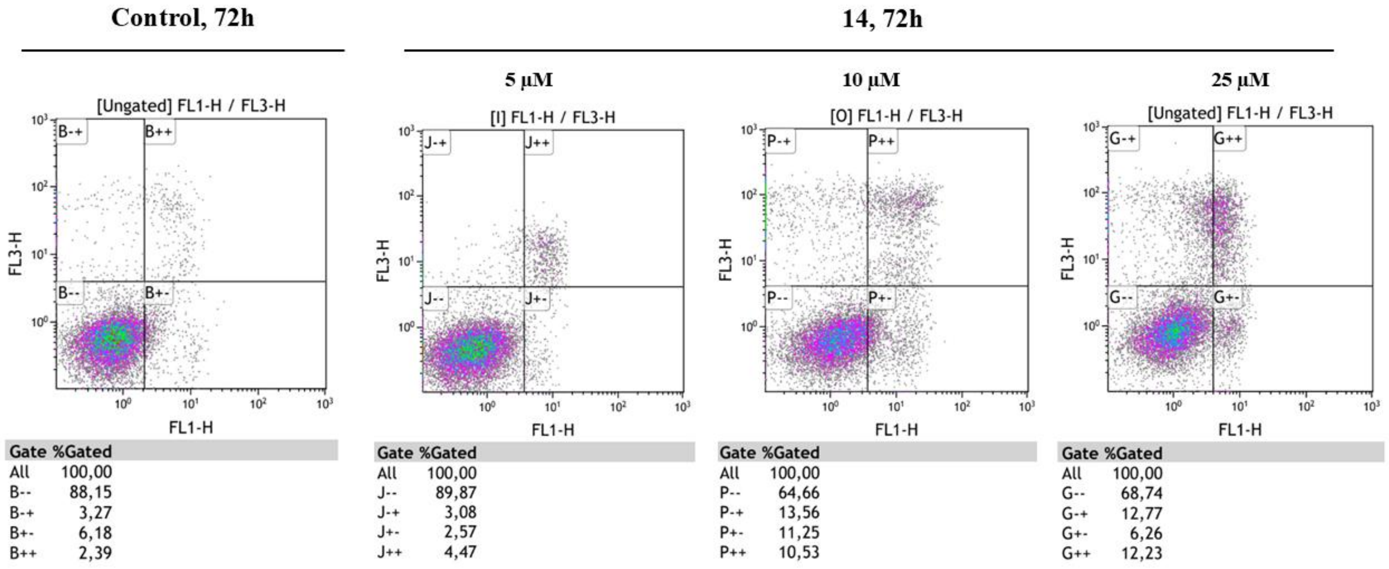

References

1) Giovannuzzi, S.; D'Ambrosio, M.; Luceri, C.; Osman, S. M.; Pallecchi, M.; Bartolucci, G.; Nocentini, A.; Supuran, C. T. Aromatic Sulfonamides including a Sulfonic Acid Tail: New Membrane Impermeant Carbonic Anhydrase Inhibitors for Targeting Selectively the Cancer-Associated Isoforms. *Int J Mol Sci*. **2021**, *23*, 461.

2) Bonardi, A.; Nocentini, A.; Giovannuzzi, S.; Paoletti, N.; Ammara, A.; Bua, S.; Abutaleb, N. S.; Abdelsattar, A. S.; Capasso, C.; Gratteri, P.; Flaherty, D. P.; Seleem, M. N.; Supuran, C. T. Development of Penicillin-Based Carbonic Anhydrase Inhibitors Targeting Multidrug-Resistant Neisseria gonorrhoeae. *J Med Chem* **2024**, *11*, 9613-9627.

3) Grandane, A.; Belyakov, S.; Trapencieris, P.; Zalubovskis, R. Facile synthesis of coumarin bioisosteres—1,2-benzoxathiine 2,2-dioxides. *Tetrahedron* **2012**, *68*, 5541-5546.

4) Suebsuwong, C.; Pinkas, D. M.; Ray, S. S.; Bufton, J. C.; Dai, B.; Bullock, A. N.; Degterev, A.; Cuny, G. D. Activation loop targeting strategy for design of receptor-interacting protein kinase 2 (RIPK2) inhibitors. *Bioorg Med Chem Lett* **2018**, *4*, 577-583.

5) Bozdag, M.; Alafeefy, A. M.; Altamimi, A. M.; Carta, F.; Supuran, C. T.; Vullo, D. Synthesis of new 3-(2-mercapto-4-oxo-4H-quinazolin-3-yl)-benzenesulfonamides with strong inhibition properties against the tumor associated carbonic anhydrases IX and XII. *Bioorg Med Chem* **2017**, *10*, 2782-2788.

6) Wong, R.; Dolman, S. J. Isothiocyanates from tosyl chloride mediated decomposition of in situ generated dithiocarbamic acid salts. *J Org Chem* **2007**, *10*, 3969-3971.

7) Tietze, S.; Apostolakis, J.; GlamDock: development and validation of a new docking tool on several thousand protein-ligand complexes. *J Chem Inf Model*, **2007**, *47*, 1657-1672.

8) Korb, O.; Stutzle, T.; Exner, T. E. Empirical scoring functions for advanced protein-ligand docking with PLANTS. *J Chem Inf Model*, **2009**, *49*, 84-96.

9) Liu, N.; Xu, Z. B. Using LeDock as a docking tool for computational drug design. *Iop C Ser Earth Env*, **2019**, *218*, 0121443.

10) Hsu, K. C.; Chen, Y. F.; Lin, S. R.; Yang, J. M. iGEMDOCK: a graphical environment of enhancing GEMDOCK using pharmacological interactions and post-screening analysis. *BMC Bioinformatics*, **2011**, *12*, S33.

11) Quiroga, R.; Villarreal, M. A. Vinardo: A Scoring Function Based on Autodock Vina Improves Scoring, Docking, and Virtual Screening. *PLoS One*, **2016**, *11*, e0155183.

12) Ng, M. C.; Fong, S.; Siu, S. W. PSOVina: The hybrid particle swarm optimization algorithm for protein-ligand docking. *J Bioinform Comput Biol*, **2015**, *13*, 1541007.

13) Alhossary, A.; Handoko, S. D.; Mu, Y.; Kwoh, C. K. Fast, accurate, and reliable molecular docking with QuickVina 2. *Bioinformatics*, **2015**, *31*, 2214-2216.

14) Eberhardt, J.; Santos-Martins, D.; Tillack, A. F.; Forli, S. AutoDock Vina 1.2.0: New Docking Methods, Expanded Force Field, and Python Bindings. *J Chem Inf Model*, **2021**, *61*, 3891-3898.

15) Koebel, M. R.; Cooper, A.; Schmadeke, G.; Jeon, S.; Narayan, M.; Sirimulla, S. S...O and S...N Sulfur Bonding Interactions in Protein-Ligand Complexes: Empirical Considerations and Scoring Function. *J Chem Inf Model*, **2016**, *56*, 2298-2309.

16) Koebel, M. R.; Schmadeke, G.; Posner, R. G.; Sirimulla, S. AutoDock VinaXB: implementation of XBSF, new empirical halogen bond scoring function, into AutoDock Vina. *J Cheminform*, **2016**, *8*, 27.

17) Hassan, N. M.; Alhossary, A. A.; Mu, Y.; Kwoh, C. K. Protein-Ligand Blind Docking Using QuickVina-W With Inter-Process Spatio-Temporal Integration. *Sci Rep*, **2017**, *7*, 15451.

18) Paul, D. S.; Gautham, N. iMOLSDOCK: Induced-fit docking using mutually orthogonal Latin squares (MOLS). *J Mol Graph Model*, **2017**, *74*, 89-99.

19) Li, C.; Li, J.; Sun, J.; Mao, L.; Palade, V.; Ahmad, B. Parallel multi-swarm cooperative particle swarm optimization for protein-ligand docking and virtual screening. *BMC Bioinformatics*, **2022**, *23*, 201.

20) Masters, L.; Eagon, S.; Heying, M. Evaluation of consensus scoring methods for AutoDock Vina, smina and idock. *J Mol Graph Model* **2020**, *96*, 107532.

21) Kim, D. S.; Kim, C. M.; Won, C. I.; Kim, J. K.; Ryu, J.; Cho, Y.; Lee, C.; Bhak, J. BetaDock: shape-priority docking method based on beta-complex. *J Biomol Struct Dyn*, **2011**, *29*, 219-242.

22) Khalifah, R.G. The carbon dioxide hydration activity of carbonic anhydrase. I. Stop-flow kinetic studies on the native human isoenzymes B and C. *J Biol Chem*, **1971**, *246*, 2561-2573.
